# Syntheses of 3,4- and 1,4-dihydroquinazolines from 2-aminobenzylamine

**DOI:** 10.3762/bjoc.13.145

**Published:** 2017-07-27

**Authors:** Jimena E Díaz, Silvia Ranieri, Nadia Gruber, Liliana R Orelli

**Affiliations:** 1 Universidad de Buenos Aires. CONICET. Departamento de Química Orgánica. Facultad de Farmacia y Bioquímica. Junín 956, (1113) Buenos Aires, Argentina; 2 Department of Industrial Chemistry “Toso Montanari”, University of Bologna, Viale Risorgimento 4, 40136 Bologna, Italy

**Keywords:** dihydroquinazolines, microwaves, *N*-acylations, *N*-alkylations, PPE

## Abstract

A straightforward strategy for the synthesis of dihydroquinazolines is presented, which allows for the preparation of 3,4- and 1,4-dihydroquinazolines with different substitution patterns from 2-aminobenzylamine (2-ABA) as common precursor. The required functionalization of both amino groups present in 2-ABA was achieved by different routes involving selective *N-*acylation and cesium carbonate-mediated *N-*alkylation reactions, avoiding protection/deprotection steps. The heterocycles were efficiently synthesized in short reaction times by microwave-assisted ring closure of the corresponding aminoamides promoted by ethyl polyphosphate (PPE).

## Introduction

Nitrogen heterocycles are part of many drugs and represent structures with wide therapeutic potential. Therefore, much effort has been devoted to the development of efficient, general and expeditious methods for their synthesis [1–2]. Cyclic amidines are compounds of interest due to their multiple pharmacological properties, some of which derive from their interaction with a specific receptor [3–4]. Six-membered heterocylic amidines (1,4,5,6-tetrahydropyrimidines) display biological activity as anthelmintics used in medical practice (pyrantel, morantel and oxantel) [5–6], nicotinic agonists [7], antidepressants [8] and selective inhibitors of M1 acetylcholine receptors [9–10], among others. Their benzannulated analogues, dihydroquinazolines, also represent heterocyclic cores of pharmacological interest. Some derivatives containing this motif have shown antimicrobial [11] and antifungal properties [12]. Their activity as selective T-type calcium channel blockers [13–15] and as inhibitors of β-secretase (an important target for Alzheimer’s disease) has been reported [16]. They have also been studied as inhibitors of trypanothione reductase [17], an essential enzyme of the parasite *Trypanosoma brucei,* and as neuroprotective agents [18].

Different strategies have been used for the preparation of dihydroquinazolines. Some of them involve cyclic precursors such as quinazolinones, quinazolines or tetrahydroquinazolines, which can be reduced or oxidized to yield dihydroquinazolines [17,19–25]. Heterocyclization of acyclic precursors has also been applied for the synthesis of these heterocycles. Many methods use 2-aminobenzylamine (2-ABA) that reacts with amidines, carboxylic acids, orthoformates, or aldehydes in oxidative conditions to yield *N-*unsubstituted dihydroquinazolines [11,16,26–35]. A different approach is based on the heterocyclization of functionalized 2-aminobenzylamines [36–37] such as the cyclization of *N-*(2-aminobenzyl)amides, which in general requires drastic conditions or long reaction times [17–18,21–22,29,38–40]. Miscellaneous methods include the nucleophilic addition of secondary amines to carbodiimides followed by an intramolecular conjugate addition to an α,β*-*unsaturated ester [12–15] or copper-catalyzed annulation of *N-*arylamidines [41], among others [42]. Most of the already mentioned methods have been applied to the synthesis of 3,4-dihydroquinazolines. Among them, only a few were used for the preparation of 1,4-dihydroquinazolines, a heterocyclic framework quite unexplored.

In addition to the heterocyclization itself, the preparation of the acyclic precursors for the synthesis of dihydroquinazolines remains challenging as in some cases it includes a selective *N*-functionalization of the amino groups of 2-ABA. For example, the selective acylation of the aliphatic amino group was achieved by the treatment of the diamine with benzoic acid in the presence of Zr(azobenzene-4,4´-dicarboxylate) [43], with *tert-*butylperoxybenzoate [44] and with isopropenyl acetate [45], or by a DCC-mediated coupling with a carboxylic acid [38]. To our knowledge, selective acylations of *N*^1^-alkyl-2-ABAs have not been reported yet.

Alkylations of the aliphatic amino group of 2-ABA using triflates, halogen or CCl_3_ derivatives have also been reported. However, these reactions are frequently associated with long reaction times, poor yields and low selectivity [46–47]. The selective monofunctionalization of 2-ABA was achieved by using protective groups such as 9-BBN [48] or Boc [39], which require subsequent removal. Other alternative synthetic strategies use substrates like 2-nitrobenzoyl chloride, 2-nitrobenzaldehyde, 2-aminobenzamides, 2-aminobenzophenone, 2-nitrobenzyl bromide, among others, which require an additional reduction step [17–18,29,49–51].

Ethyl polyphosphate (PPE) and trimethylsilyl polyphosphate (PPSE) are mild irreversible dehydrating agents of the Lewis acid type. They have found application for synthetically useful transformations like conversion of amides into nitriles [52–53] or the Beckmann rearrangement [52,54]. They have also been widely employed in heterocyclic synthesis, including microwave-assisted reactions. The recognized wide functional group tolerance, stability and low environmental impact are additional advantages of these reagents. Cyclocondensation is a fundamental tool in heterocycles synthesis, and microwave (MW) heating has found interesting applications in this area [55–56], overcoming limitations arising from low reactivity and side reactions typical of conventional heating [57].

We had previously reported the MW-assisted synthesis of 1,4,5,6-tetrahydropyrimidines and their homologues by cyclodehydration reactions promoted by polyphosphoric acid (PPA) esters PPE and PPSE [58–60]. This strategy was successfully applied to the synthesis of 1-aryl-2-imino-1-azacycloalkanes [61], 2-oxazolines, 5,6-dihydro-4*H*-1,3-oxazines and 4,5,6,7-tetrahydro-1,3-oxazepines [62].

**Figure 1:**
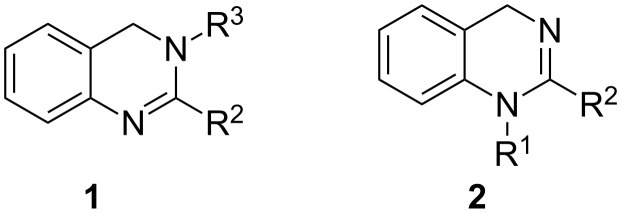
3,4-Dihydroquinazolines **1** and 1,4-dihydroquinazolines **2**.

In continuation of this work we herein present an efficient strategy for the synthesis of dihydroquinazolines starting from the easily available 2-ABA as common precursor. The route allows for the preparation of 3,4-dihydroquinazolines **1** and 1,4-dihydroquinazolines **2** with different substitution patterns (Figure 1).

## Results and Discussion

**Scheme 1:**
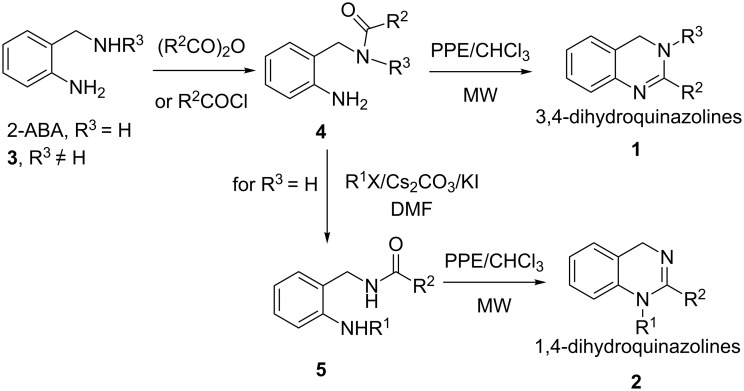
Synthetic pathways for the preparation of 3,4-dihydroquinazolines **1** and 1,4-dihydroquinazolines **2**.

The general synthetic pathways leading to the desired dihydroquinazolines **1** and **2** are depicted in Scheme 1.

**Scheme 2:**
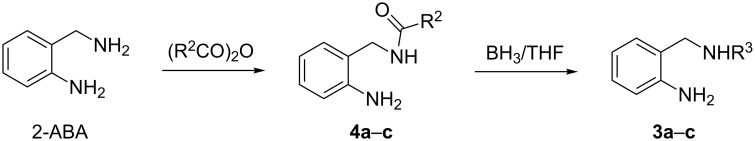
Synthesis of compounds **3a**–**c**.

*N*-Alkyl-substituted 2-aminobenzylamines **3a**–**c** were readily synthesized by reduction of the amides **4a**–**c** employing a THF solution of borane (yields 82–93%; see Supporting Information File 1 and Scheme 2).

The synthesis of *N*-(2-aminobenzyl)amides **4a–k** was achieved by selective *N*-acylation of 2-ABA or its *N*-substituted derivatives **3a–c**, whose preparation is depicted in Scheme 2. In spite of the different nucleophilicity of both amino groups, the predictable formation of a hydrogen bond between the aromatic amino group (hydrogen bond donor) and the aliphatic NHR (hydrogen bond acceptor) could increase the nucleophilicity of the former and decrease the reactivity of the latter, favoring the chance for *N*,*N*´-diacylation. To avoid this, the reaction was performed employing a diluted solution of the amine in a biphasic system (CHCl_3_/aqueous NaOH), low temperature (0 °C) and the use of anhydrides as acylating agents, if commercially available. The results are shown in Table 1.

**Table 1:** Selective *N*-acylation of 2-ABA and its derivatives.

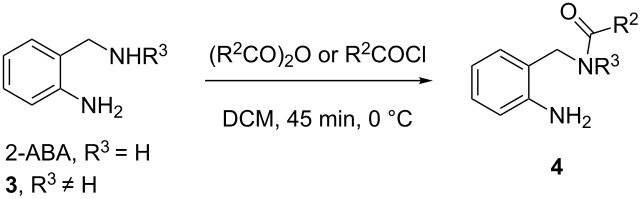					

Entry	**4**	R^3^	R^2^	Acylation agent	Yield (%)^a^

1	**a**	H	CH_3_	anhydride	90
2	**b**	H	CH_2_CH_3_	anhydride	93
3	**c**	H	CH(CH_3_)_2_	anhydride	94
4	**d**	H	C(CH_3_)_3_	anhydride	93
5	**e**	H	C_6_H_5_	anhydride	94
6	**f**	H	2-CH_3_C_6_H_4_	acyl chloride	83
7	**g**	H	2-FC_6_H_4_	acyl chloride	70
8	**h**	CH_2_CH_3_	CH_3_	anhydride	81
9	**i**	CH_2_CH_3_	CH_2_CH_3_	anhydride	80
10	**j**	CH_2_CH_2_CH_3_	CH_2_CH_3_	anhydride	72
11	**k**	CH_2_CH(CH_3_)_2_	CH_2_CH_3_	anhydride	79

^a^Yields correspond to pure compounds.

For R^3^ = H, products **4a–e** were obtained in excellent yields when 2-ABA was treated with acid anhydrides as the acylating agents (Table 1, entries 1–5). Replacing anhydrides by acyl chlorides in the reaction the yields were slightly lower, and in some cases small amounts of the *N,N’*-diacylated products were also obtained (Table 1, entries 6 and 7). Under the same reaction conditions, the *N-*substituted derivatives **3** (R^3^ ≠ H) were selectively acylated with very good yields (Table 1, entries 8–11). Thus, precursors **4** were efficiently prepared by a sequence of *N-*acylation–reduction–*N-*acylation.

The acyclic precursors **5**, necessary for the synthesis of dihydroquinazolines **2**, were prepared by *N*-alkylation of the arylamino group present in compounds **4** using alkyl halides. In a previous work [63], we developed a methodology for the selective *N*-alkylation of anilines with ω*-*halonitriles in the presence of cesium carbonate and potassium iodide. There the use of the cesium base prevented *N*-dialkylation and therefore it was not necessary to use an excess of the amine. This led us to test the same reaction conditions for the synthesis of compounds **5**. In this case, the presence of the amide functionality was regarded as an additional problem, as Cs_2_CO_3_-mediated alkylation of cyclic amides had already been reported in the literature [64]. In spite of this, compounds **5** were obtained in good to high yields (Table 2) employing the same experimental conditions [63]. The reaction times and temperatures were individually adjusted taking into account the boiling points of the alkylating agents and the reactivity of each substrate.

**Table 2:** Synthesis of compounds **5**.

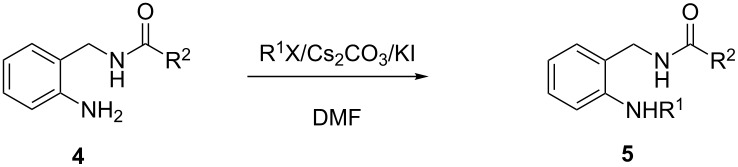							

Entry	**5**	R^2^	R^1^	X	Time (h)	Temperature (°C)	Yield (%)^a^

1	**a**	CH_3_	CH_2_CH_2_CH_3_	I	5	75	81
2	**b**	CH_3_	CH_2_CH=CH_2_	Br	4.5	65	68
3	**c**	CH_3_	CH_2_C_6_H_5_	Cl	5	90	64
4	**d**	CH_3_	CH(CH_3_)_2_	I	6	85	60
5	**e**	CH(CH_3_)_2_	CH_2_CH_2_CH_3_	I	6.5	85	67
6	**f**	C(CH_3_)_3_	CH_2_CH_2_CH_3_	I	6	85	69
7	**g**	C(CH_3_)_3_	CH_2_C_6_H_5_	Cl	3	90	60
8	**h**	C(CH_3_)_3_	CH(CH_3_)_2_	I	7	85	75
9	**i**	C_6_H_5_	CH_2_CH_2_CH_3_	I	7	85	55
10	**j**	2-CH_3_C_6_H_4_	CH_2_CH_2_CH_3_	I	10	85	71

^a^Yields correspond to pure compounds.

As expected, the reaction was selective toward the *N*-monoalkylation of the arylamino group in the presence of the amide. Satisfactory results were also obtained using the more hindered isopropyl iodide (Table 2, entries 4 and 8).

Next, the conversion of *N*-(2-aminobenzyl)acetamide (**4a**) into 2-methyl-3,4-dihydroquinazoline (**1a**) was chosen for the optimization of the reaction conditions for the synthesis of compounds **1**. No background reaction was observed when heating a solution of compound **4a** in chloroform for 10 minutes at 130 °C under MW irradiation in a closed vessel. Using a solution of PPSE in DCM as cyclodehydrating agent, after 30 minutes of reaction under reflux using conventional heating compound **1a** was obtained in 86% yield. Analogous results were obtained using either PPE/CHCl_3_ or PPSE/CH_2_Cl_2_ under microwave irradiation at 110 ºC for 4 minutes. Due to the easier preparation and the inexpensive reagents required for its synthesis [65], PPE was chosen as cyclodehydrating agent for the preparation of compounds **1**. Results and optimized experimental conditions of the MW-assisted ring-closure reaction are depicted in Table 3. Due to the different reactivity of each substrate, irradiation times and temperatures were individually adjusted.

**Table 3:** Synthesis of 3,4-dihydroquinazolines **1**.

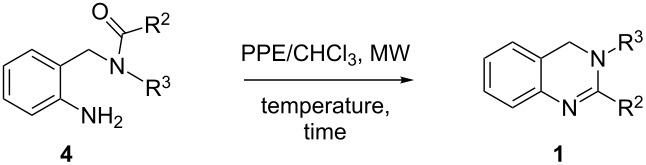						

Entry	Product **1**	R^3^	R^2^	Time (min)	Temperature (°C)	Yield (%)^a^

1	**a**	H	CH_3_	4	110	86
2	**b**	H	CH_2_CH_3_	4	110	84
3	**c**	H	CH(CH_3_)_2_	9	120	76
4	**d**	H	C(CH_3_)_3_	12	130	73
5	**e**	H	C_6_H_5_	12	130	71
6	**f**	H	2-CH_3_C_6_H_4_	15	130	79
7	**g**	H	2-FC_6_H_4_	15	130	60
8	**h**	CH_2_CH_3_	CH_3_	4.5	100	98
9	**i**	CH_2_CH_3_	CH_2_CH_3_	8	115	79
10	**j**	CH_2_CH_2_CH_3_	CH_2_CH_3_	8	115	86
11	**k**	CH_2_CH(CH_3_)_2_	CH_2_CH_3_	8	115	80

^a^Yields correspond to pure compounds.

For the synthesis of compounds **1** the reaction conditions mainly depended on the steric and electronic features of the amido group present in the substrates. Acetamides and propionamides **4a**,**b**,**h**–**k** reacted readily under mild reaction conditions (Table 3, entries 1, 2, and 8–11), while for isobutyramide **4c** a higher temperature was necessary (Table 3, entry 3). Finally, the more sterically hindered pivalamide **4d** and the less reactive benzamides **4e**–**g** required harsher reaction conditions to achieve complete conversion of the substrates and the products were afforded in generally lower yields (Table 3, entries 4–7).

**Scheme 3:**
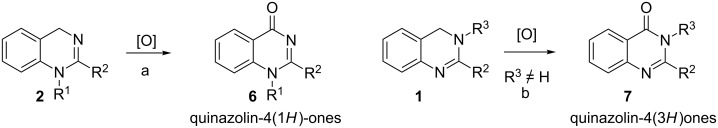
Benzylic oxidation of 1,4-dihydroquinazolines (a) and 3,4-dihydroquinazolines (b).

In order to widen the scope of the method, we examined next the applicability of the PPE/MW system to the synthesis of 1,4-dihydroquinazolines **2**. Treatment of precursor **5a** with PPE/CHCl_3_ under microwave irradiation led to 1,4-dihydroquinazoline **2a**, accompanied by a low percentage of a collateral product. This compound was identified as 2-methyl-1-propylquinazolin-4(1*H*)-one (**6a**) (Scheme 3, (a), R^1^ = Pr, R^2^ = Me), arising from spontaneous benzylic oxidation of **2a**. An analogous reaction had been previously reported for 3,4-dihydroquinazolines upon exposure to air (Scheme 3, (b)) [19,24,40,66].

The fact that quinazolin-4(1*H*)-ones **6**, which are fully conjugated push–pull molecules, are more resonance stabilized than their isomeric counterparts quinazolin-4(3*H*)-ones **7**, would explain the differential behavior of *N-*substituted 3,4- and 1,4-dihydroquinazolines toward spontaneous oxidation.

By the same procedure used for **2a**, a series of novel 1,4-dihydroquinazolines were synthesized. The results and optimized reaction conditions are reported in Table 4. Due to the different reactivity of each substrate, irradiation times and temperatures were individually adjusted.

**Table 4:** Synthesis of 1,4-dihydroquinazolines **2**.

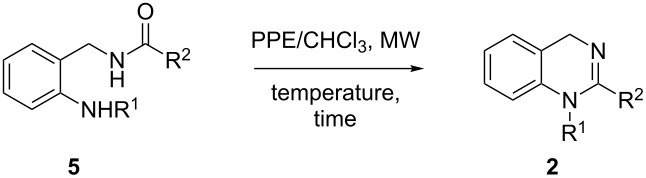						

Entry	Product **2**	R^2^	R^1^	Time (min)	Temperature (°C)	Yield (%)^a^

1	**a**	CH_3_	CH_2_CH_2_CH_3_	11	80	67
2	**b**	CH_3_	CH_2_CH=CH_2_	11	90	75
3	**c**	CH_3_	CH_2_C_6_H_5_	11	90	72
4	**d**	CH_3_	CH(CH_3_)_2_	12	130	91
5	**e**	CH(CH_3_)_2_	CH_2_CH_2_CH_3_	21	110	79
6	**f**	C(CH_3_)_3_	CH_2_CH_2_CH_3_	20	130	68
7	**g**	C(CH_3_)_3_	CH_2_C_6_H_5_	25	130	–
8	**h**	C(CH_3_)_3_	CH(CH_3_)_2_	25	130	–
9	**i**	C_6_H_5_	CH_2_CH_2_CH_3_	13	90	25^b^
10	**j**	2-CH_3_C_6_H_4_	CH_2_CH_2_CH_3_	5	100	39^b^

^a^Yields correspond to pure compounds. ^b^The reported yield is approximate and was estimated by integration of suitable ^1^H NMR signals (see Supporting Information File 1).

Generally the yields for compounds **2** were good to excellent and the experimental conditions and reaction outcome varied depending on the electronic and steric features of the substituents R^1^ and R^2^ present in precursors **5**. The cyclodehydration of acetamides **5a**–**c** proceeded under mild reaction conditions (Table 4, entries 1–3), while acetamide **5d** required a higher temperature probably due to the steric hindrance produced by the *N*-isopropyl moiety (Table 4, entry 4). A higher temperature and a longer reaction time were necessary in order to achieve cyclodehydration of isobutyramide **5e**, and even harsher conditions were required for pivalamide **5f** (Table 4, entries 5 and 6). In the case of precursors **5g**,**h**, instead of the expected 1-substituted-2-*tert*-butyl-1,4-dihydroquinazolines, cyclodehydration led to 2-*tert*-butyl-3,4-dihydroquinazoline (**1d,** Table 4, entries 7 and 8), with loss of one *N*-substituent. A possible explanation is that the reaction conditions required in those cases due to steric hindrance bring about a competitive elimination reaction. Finally, as already mentioned, 1,4-dihydroquinazolines **2** are prone to spontaneous oxidation leading to 4(1*H*)-quinazolinones (Scheme 3a) and dihydroquinazolines **2i**,**j** showed a comparatively higher lability toward this side reaction. For this reason, the cyclodehydration of the substrates **5i**,**j** was carried out at lower temperatures in order to avoid benzylic oxidation. In spite of this, partial oxidation during workup and purification could not be avoided accounting for the lower yields obtained for the 2-aryl derivatives **2i**,**j** (Table 4, entries 9 and 10). The higher sensitivity of compounds **2i**,**j** towards oxidation may be a consequence of the enhanced resonance stabilization of the resulting 2-arylquinazolin-4(1*H*)-ones **6i**,**j** (Scheme 3). The fact that the yield of the *o*-methylphenyl-substituted derivative **2j**, in which this stabilization is reduced due to steric effects, was higher than for the phenyl-substituted derivative reinforces this hypothesis.

## Conclusion

We have developed novel, general and straightforward strategies for the synthesis of 3,4-dihydroquinazolines with different substitution patterns, starting from 2-ABA as common precursor. An extension of the method allowed for the preparation of *N-*alkyl-1,4-dihydroquinazolines, a heterocyclic nucleus almost unexplored. To our knowledge, this is the first general method available for synthesis of these compounds. The products were synthesized by microwave-assisted ring closure of different aminoamides promoted by PPE. The yields and reaction times involved in the heterocyclization step compare favorably with the reported methodologies. The preparation of the required aminoamides involves *N-*acylation and *N-*alkylation reactions, which were optimized to achieve selective monofunctionalization thus avoiding additional protection/deprotection steps. A judicious combination of these steps into different sequences, allowed for the preparation of both 3,4- and 1,4-dihydroquinazolines with the desired *N-*substitution starting from a single precursor.

The protocols are operationally simple and involve easily available and inexpensive reagents. Due to their widespread use in organic synthesis, the selective *N-*functionalization reactions described herein may find application in many different molecular contexts.

## Supporting Information

File 1 Experimental procedures and characterization of new compounds.
