# Supplementary material for: Syntheses of 3,4- and 1,4-dihydroquinazolines from 2-aminobenzylamine
Source: Beilstein J Org Chem. 2017 Jul 27;13:1470–7. doi: 10.3762/bjoc.13.145 (PMC5550820; doi:10.3762/bjoc.13.145)
Supplement: File 1 — Experimental procedures and characterization of new compounds. [file Beilstein_J_Org_Chem-13-1470-s001.pdf]

Supporting Information

for

Syntheses of 3,4- and 1,4-dihydroquinazolines from 2-aminobenzylamine

Jimena E. Díaz[1], Silvia Ranieri[1,2], Nadia Gruber[1] and Liliana R. Orelli[1*]

Address: [1]Universidad de Buenos Aires. CONICET. Departamento de Química Orgánica. Facultad de Farmacia y Bioquímica. Junín 956, (1113) Buenos Aires, Argentina and [2]Department of Industrial Chemistry "Toso Montanari", University of Bologna, Viale Risorgimento 4, 40136 Bologna, Italy

Email: Liliana R. Orelli - lorelli@ffyb.uba.ar

*Corresponding author

Experimental procedures and characterization of new compounds

Table of Contents:

1. General information	S2
2. Representative procedures for synthesis	S2
3. Characterization data for compounds **1–6**	S3
4. Copies of ^{1}H and ^{13}C NMR Spectra of compounds **1–6**	S12
5. References	S56

1. General Information

Melting points were determined with a Büchi capillary apparatus and are uncorrected. ^{1}H and ^{13}C NMR spectra were recorded on a Bruker Bio Spin Avance III 600 MHz spectrometer, a Bruker Avance II 500 MHz spectrometer or a Bruker MSL 300 MHz spectrometer, using deuteriochloroform as the solvent. Chemical shifts are reported in ppm (δ) relative to TMS as an internal standard. D_2O was employed to confirm exchangeable protons (ex). Splitting multiplicities are reported as singlet (s), broad signal (bs), doublet (d), double doublet (dd), doublet of doublets of doublets (ddd), triplet (t), triplet of doublets (td), quartet (q), pentet (p), sextet (sext), heptet (h), nonet (non) and multiplet (m). HRMS (ESI) were performed with a Bruker MicroTOF-Q II spectrometer. Reagents, solvents and starting materials were purchased from standard sources and purified according to literature procedures.

2. Representative procedures for synthesis

General Procedure for the synthesis of 3,4-dihydroquinazolines 1 and 1,4-dihydroquinazolines 2: A mixture of the corresponding compound **4** or **5** (0.5 mmol) and a chloroform solution of PPE [1] (2 mL) was reacted in a microwave reactor (Monowave 300, Anton Paar) at the indicated temperature and time. After reaching room temperature, the resulting solution was extracted with water (5 × 3 mL). The aqueous phases containing the protonated compounds **1** or **2** were pooled, filtered and made alkaline with 10% NaOH in an ice bath, and the mixture was extracted with dichloromethane (3 × 30 mL). The organic layer was washed with water (5 mL), dried over sodium sulfate and filtered. The solvent was removed in vacuo. The crude products were purified by column chromatography (silica gel 60, DCM/methanol/isopropylamine).

General Procedure for the synthesis of 2-((alkylamino)methyl)anilines 3: A solution of compound **4** (1 mmol) in tetrahydrofuran (30 mL) was treated with freshly generated borane [2]. The solution was refluxed for 1 h, cooled and treated with methanol. The solvent was then removed in vacuo. The residue was refluxed with 10% hydrochloric acid (30 mL) and filtered. The filtrate, containing compounds **3** as hydrochlorides, was made alkaline with 10% aqueous NaOH and subsequently extracted with dichloromethane (2 × 20 mL). The organic phases were pooled, washed with water (5 mL), dried over sodium sulfate and filtered. The solvent was removed in

vacuo. The crude products were purified by column chromatography (silica gel 60, DCM/methanol/isopropylamine).

General Procedure for the synthesis of *N-(*2-aminobenzyl)amides 4: The corresponding acylating agent (1 mmol) dissolved in dichloromethane (5 mL) was added dropwise to a flask containing a dichloromethane solution of 2-ABA or the suitable compound **3** (1 mmol in 30 mL) and an aqueous solution of NaOH 10% (10 mL) in an ice bath. The mixture was vigorously shaken during 45 minutes, after which the organic layer was separated. The aqueous solution was extracted with dichloromethane (2 × 10 mL). The organic layers were pooled, washed with water (5 mL), dried over anhydrous sodium sulfate and filtered. The solvent was removed in vacuo, and the residue purified by column chromatography (silica gel 60, hexane/ethyl acetate).

General Procedure for the synthesis of *N*-(2-(alkylamino)benzyl)amides 5: A solution of the corresponding alkyl halide (3.125 mmol) in dimethylformamide (1 mL) was added during 1.5 h to a mixture of the corresponding compound **4** (2.5 mmol), Cs_2CO_3 (2.5 mmol) and KI (5 mmol) in dimethylformamide (2.5 mL). The mixture was stirred at the indicated temperature and time. After completion of the reaction, as evidenced by TLC, the mixture was treated with ethyl ether (50 mL) and water (10 mL). The aqueous phase was separated and extracted with ethyl ether (30 mL). The combined organic layers were dried over anhydrous sodium sulfate and filtered. The solvent was evaporated in vacuo and the crude products were purified by column chromatography (silica gel 60, DCM/ethyl acetate).

3. Characterization data for compounds **1–5**

Compounds **1e** [3,4], **3a** [5], **3b** [5], **4a** [5-7] and **4e** [8,9] were described in the literature.

Compound **1a** was described in the literature [10]. Its spectral data are as follows.

2-Methyl-3,4-dihydroquinazoline (1a): Yellow oil (86% yield). ^{1}H NMR (500 MHz, $CDCl_3$): δ 2.00 (s, 3H), 4.60 (s, 2H), 5.74 (bs, ex, 1H), 6.85-6.87 (m, 2H), 6.94 (td, *J*= 7.3, 1.1 Hz, 1H), 7.08-7.11 (m, 1H). ^{13}C NMR (125 MHz, $CDCl_3$): δ 22.1, 44.5, 119.5, 119.9, 123.8, 125.5, 127.8, 140.7, 155.4. HRMS (ESI) [M+H]+ m/z calcd for $C_9H_{11}N_2$: 147.0917. Found: 147.0922.

2-Ethyl-3,4-dihydroquinazoline (1b): White solid (84% yield), mp: 92-94°C. ^{1}H NMR (500 MHz, $CDCl_3$): δ 1.22 (t, *J*= 7.6 Hz, 3H), 2.30 (c, *J*= 7.6 Hz, 2H), 4.25 (bs, ex, 1H), 4.63 (s, 2H), 6.87-6.89 (m, 2H), 6.97 (td, *J*= 7.3, 1.1 Hz, 1H), 7.10-7.14 (m, 1H). ^{13}C NMR (125 MHz, $CDCl_3$): δ 11.3, 29.2, 44.9, 119.7, 123.9, 125.6, 127.9, 140.3, 159.3. HRMS (ESI) [M+H]+ m/z calcd for $C_{10}H_{13}N_2$: 161.1073. Found: 161.1069.

2-Isopropyl-3,4-dihydroquinazoline (1c): White solid (76% yield), mp: 66-68°C. ^{1}H NMR (500 MHz, $CDCl_3$): δ 1.23 (d, *J*= 7.1 Hz, 6H), 2.51 (h, *J*= 7.1 Hz, 1H), 4.07 (bs, ex, 1H), 4.63 (s, 2H), 6.87-6.90 (m, 2H), 6.97 (td, *J*= 7.4, 1.1 Hz, 1H), 7.11-7.14 (m, 1H). ^{13}C NMR (125 MHz, $CDCl_3$): δ 20.3, 34.9, 45.1, 119.4, 119.9, 123.8, 125.6, 127.9, 144.7, 160.5. HRMS (ESI) [M+H]+ m/z calcd for $C_{11}H_{15}N_2$: 175.1230. Found: 175.1228.

2-*tert*-Butyl-3,4-dihydroquinazoline (1d): Yellow solid (73% yield), mp: 117-119°C. ^{1}H NMR (500 MHz, $CDCl_3$): δ 1.26 (s, 9H), 4.60 (s, 2H), 5.11 (bs, ex, 1H), 6.85 (d, *J*= 7.6 Hz, 1H), 6.91-6.92 (m, 1H), 6.96 (td, *J*= 7.4, 1.1 Hz, 1H), 7.10-7.14 (m, 1H). ^{13}C NMR (125 MHz, $CDCl_3$): δ 27.8, 37.0, 44.3, 118.3, 124.9, 125.4, 128.1, 145.0, 166.2. HRMS (ESI) [M+H]+ m/z calcd for $C_{12}H_{17}N_2$: 189.1386. Found: 189.1392.

2-Phenyl-3,4-dihydroquinazoline (1e): White solid (71% yield), mp: 133-135°C. ^{1}H NMR (500 MHz, $CDCl_3$): δ 3.24 (bs, ex, 1H), 4.79 (s, 2H), 6.96 (d, *J*= 7.6 Hz, 1H), 7.04 (td, *J*= 7.3, 1.1 Hz, 1H), 7.09-7.10 (m, 1H), 7.18-7.21 (m, 1H), 7.41-7.48 (m, 3H), 7.80-7.82 (m, 2H). ^{13}C NMR (125 MHz, $CDCl_3$): δ 44.7, 120.1, 121.5, 124.6, 125.5, 126.5, 128.1, 128.6, 130.6, 135.1, 141.0, 154.9.

2-(2-Methylphenyl)-3,4-dihydroquinazoline (1f): Yellow oil (79% yield). ^{1}H NMR (600 MHz, $CDCl_3$): δ 2.45 (s, 3H), 4.75 (s, 2H), 6.94 (d, *J*= 7.3, 1H), 6.98 (bs, 1H), 7.04 (t, *J*= 7.3 Hz, 1H), 7.15-7.22 (m, 3H), 7.29 (t, *J*= 7.3 Hz, 1H), 7.38 (d, *J*= 7.3 Hz, 1H). ^{13}C NMR (150 MHz, $CDCl_3$): δ 19.3, 45.0, 119.7, 124.5, 125.6, 125.9, 127.97, 128.0, 129.5, 130.7, 136.0, 136.1, 140.7, 156.4, 167.7. HRMS (ESI) [M+H]+ m/z calcd for $C_{15}H_{15}N_2$: 223.1230. Found: 223.1225.

2-(2-Fluorophenyl)-3,4-dihydroquinazoline (1g): Yellow oil (60% yield). ^{1}H NMR (500 MHz, $CDCl_3$): δ 4.75 (s, 2H), 5.68 (bs, ex, 1H) 6.92 (dd, J= 7.3, 0.7 Hz, 1H), 7.00-7.10 (m, 3H), 7.15-7.21 (m, 2H), 7.36-7.40 (m, 1H), 8.01 (td, J= 7.9, 1.8 Hz, 1H). ^{13}C NMR (125 MHz, $CDCl_3$): δ 44.8, 116.0 (d, *J*= 23.5 Hz), 120.0, 121.3, 122.4 (d, *J*= 10.8 Hz), 124.5, 124.5 (d, *J*= 2.9 Hz), 125.4, 127.9, 130.8 (d, *J*= 1.9 Hz), 131.8 (d, *J*= 8.8 Hz), 140.7, 160.4 (d, *J*= 247.5 Hz), 167.1. HRMS (ESI) [M+H]+ m/z calcd for $C_{14}H_{12}FN_2$: 227.0979. Found: 227.0977.

2-Methyl-3-ethyl-3,4-dihydroquinazoline (1h): Yellow oil (98% yield). ^{1}H NMR (500 MHz, $CDCl_3$): δ 1.17 (t, *J*= 7.2 Hz, 3H), 2.11 (s, 3H), 3.25 (c, *J*= 7.2 Hz, 2H), 4.45 (s, 2H), 6.81 (d, J= 7.3 Hz, 1H), 6.91 (td, *J*= 7.3, 1.1 Hz, 1H), 6.97-6.99 (m, 1H), 7.07-7.10 (m, 1H). ^{13}C NMR (125 MHz, $CDCl_3$): δ 11.9, 21.3, 45.4, 48.0, 120.7, 122.8, 123.7, 124.7, 128.0, 142.2, 156.6. HRMS (ESI) [M+H]+ m/z calcd for $C_{11}H_{15}N_2$: 175.1230. Found: 175.1230.

2,3-Diethyl-3,4-dihydroquinazoline (1i): Yellow oil (79% yield). ^{1}H NMR (500 MHz, $CDCl_3$): δ 1.22-1.27 (m, 6H), 2.43 (c, J= 7.6 Hz, 2H), 3.32 (c, *J*= 7.2 Hz, 2H), 4.50 (s, 2H), 6.85 (d, J= 7.6 Hz, 1H), 6.95 (td, *J*= 7.3, 1.1 Hz, 1H), 7.06 (d, J= 7.3 Hz, 1H), 7.12-7.15 (m, 1H). ^{13}C NMR (125 MHz, $CDCl_3$): δ 12.2, 12.5, 27.4, 45.0, 48.3, 120.8, 123.2, 123.8, 124.7, 128.2, 142.5, 160.9. HRMS (ESI) [M+H]+ m/z calcd for $C_{12}H_{17}N_2$: 189.1386. Found: 189.1382.

2-Ethyl-3-propyl-3,4-dihydroquinazoline (1j): Yellow oil (86% yield). ^{1}H NMR (500 MHz, $CDCl_3$): δ 0.95 (t, *J*= 7.3 Hz, 3H), 1.24 (t, *J*= 7.6 Hz, 3H), 1.62-1.70 (m, 2H), 2.41 (c, *J*= 7.6 Hz, 2H), 3.19 (t, *J*= 7.7 Hz, 2H), 4.49 (s, 2H), 6.84 (d, *J*= 7.4 Hz, 1H), 6.94 (td, *J*= 7.4, 1.1 Hz, 1H), 7.04-7.05 (m, 1H), 7.12-7.15 (m, 1H). ^{13}C NMR (125 MHz, $CDCl_3$): δ 11.1, 12.1, 20.5, 27.6, 48.8, 52.1, 120.9, 123.2, 123.7, 124.7, 128.1, 142.7, 161.1. HRMS (ESI) [M+H]+ m/z calcd for $C_{13}H_{19}N_2$: 203.1543. Found: 203.1550.

2-Ethyl-3-isobutyl-3,4-dihydroquinazoline (1k): Yellow oil (80% yield). ^{1}H NMR (500 MHz, $CDCl_3$): δ 0.95 (d, *J*= 6.6 Hz, 6H), 1.23 (t, *J*= 7.6 Hz, 3H), 1.99-2.07 (m, 1H), 2.42 (c, *J*= 7.6 Hz, 2H), 4.45 (s, 2H), 6.83 (dd, *J*= 7.4, 0.7 Hz, 1H), 6.94 (td, *J*= 7.4, 1.2 Hz, 1H), 7.04-7.06 (m, 1H), 7.12-7.15 (m, 1H). ^{13}C NMR (125 MHz, $CDCl_3$): δ 11.9, 19.8, 26.3, 27.6, 49.2, 57.7, 120.9, 123.1, 123.7, 124.6, 128.1, 142.6, 161.4. HRMS (ESI) [M+H]+ m/z calcd for $C_{14}H_{21}N_2$: 217.1699. Found: 217.1699.

1-Propyl-2-methyl-1,4-dihydroquinazoline (2a): Yellow solid (67% yield), mp: 38-40°C. ^{1}H NMR (500 MHz, $CDCl_3$): δ 0.96 (t, *J*= 7.4 Hz, 3H), 1.65-1.72 (m, 2H), 2.16 (s, 3H), 3.62 (t, *J*= 7.6 Hz, 2H), 4.47 (s, 2H), 6.75 (d, *J*= 8.0 Hz, 1H), 6.93-6.97 (m, 2H), 7.13-7.16 (m, 1H). ^{13}C NMR (125 MHz, $CDCl_3$): δ 11.0, 21.2, 22.1, 46.7, 48.4, 111.2, 121.8, 122.6, 125.8, 127.0, 138.5, 154.5. HRMS (ESI) [M+H]+ m/z calcd for $C_{12}H_{17}N_2$: 189.1386. Found: 189.1381.

1-Allyl-2-methyl-1,4-dihydroquinazoline (2b): Yellow oil (75% yield). ^{1}H NMR (500 MHz, $CDCl_3$): δ 2.17 (s, 3H), 4.32 (dt, *J*= 4.1, 2.1 Hz, 2H), 4.54 (s, 2H), 5.15-5.20 (m, 1H), 5.23-5.27 (m, 1H), 5.89 (ddt, *J*= 17.3, 10.6, 4.1 Hz, 1H), 6.72 (d, *J*= 8.2 Hz, 1H), 6.94-6.99 (m, 2H), 7.10-7.14 (m, 1H). ^{13}C NMR (125 MHz, $CDCl_3$): δ 21.7, 47.8, 48.3,

112.0, 116.8, 121.2, 122.8, 125.7, 127.1, 132.7, 138.7, 154.5. HRMS (ESI) [M+H]+ m/z calcd for $C_{12}H_{15}N_2$: 187.1230. Found: 187.1235.

1-Benzyl-2-methyl-1,4-dihydroquinazoline (2c): Yellow oil (72% yield). ^{1}H NMR (500 MHz, $CDCl_3$): δ 2.15 (s, 3H), 4.54 (s, 2H), 4.96 (s, 2H), 6.59 (d, *J*= 8.2 Hz, 1H), 6.95-7.04 (m, 3H), 7.20-7.22 (m, 2H), 7.25-7.27 (m, 1H), 7.32-7.35 (m, 2H). ^{13}C NMR (125 MHz, $CDCl_3$): δ 21.9, 48.1, 49.1, 112.2, 121.2, 123.0, 125.6, 125.8, 127.18, 127.22, 128.9, 136.8, 138.7, 154.7. HRMS (ESI) [M+H]+ m/z calcd for $C_{16}H_{17}N_2$: 237.1386. Found: 237.1388.

1-Isopropyl-2-methyl-1,4-dihydroquinazoline (2d): Yellow oil (91% yield). ^{1}H NMR (500 MHz, $CDCl_3$): δ 1.57 (d, *J*= 7.1 Hz, 6H), 2.26 (s, 3H), 4.31 (h, *J*= 7.1 Hz, 1H), 4.27 (s, 2H), 6.97-7.02 (m, 3H), 7.14-7.18 (m, 1H). ^{13}C NMR (125 MHz, $CDCl_3$): δ 21.5, 22.0, 48.6, 49.2, 114.0, 121.1, 122.5, 125.9, 126.5, 139.1, 156.5. HRMS (ESI) [M+H]+ m/z calcd for $C_{12}H_{17}N_2$: 189.1386. Found: 189.1384.

1-Propyl-2-isopropyl-1,4-dihydroquinazoline (2e): Yellow oil (79% yield). ^{1}H NMR (500 MHz, $CDCl_3$): δ 0.95 (t, *J*= 7.4 Hz, 3H), 1.17 (d, *J*= 6.6 Hz, 6H), 1.65-1.73 (m, 2H), 2.82 (h, *J*= 6.6 Hz, 2H), 3.70 (t, *J*= 7.3 Hz, 2H), 4.42 (s, 2H), 6.82 (d, *J*= 8.2 Hz, 1H), 6.96-7.01 (m, 2H), 7.16-7.19 (m, 1H). ^{13}C NMR (125 MHz, $CDCl_3$): δ 11.0, 21.0, 21.9, 30.3, 45.9, 48.3, 111.8, 122.6, 123.1, 125.8, 127.0, 139.6, 162.5. HRMS (ESI) [M+H]+ m/z calcd for $C_{14}H_{21}N_2$: 217.1699. Found: 217.1704.

1-Propyl-2-*tert*-butyl-1,4-dihydroquinazoline (2f): Yellow oil (68% yield). ^{1}H NMR (500 MHz, $CDCl_3$): δ 0.89 (t, *J*= 7.4 Hz, 3H), 1.33 (s, 9H), 1.56-1.63 (m, 2H), 3.96 (t, *J*= 7.0 Hz, 2H), 4.27 (s, 2H), 6.93 (d, *J*= 8.0 Hz, 1H), 6.97-7.00 (m, 1H), 7.05 (d, J= 6.6 Hz, 1H), 7.16-7.20 (m, 1H). ^{13}C NMR (125 MHz, $CDCl_3$): δ 10.8, 22.1, 29.9, 37.8, 48.0, 48.9, 113.1, 122.5, 125.3, 125.9, 126.6, 140.7, 165.0. HRMS (ESI) [M+H]+ m/z calcd for $C_{15}H_{23}N_2$: 231.1856. Found: 231.1863.

1-Propyl-2-phenyl-1,4-dihydroquinazoline (2i): The ^{1}H and ^{13}C NMR signals were obtained by difference from the spectrum of the mixture of compound **2i** and 2-phenyl-1-propylquinazolin-4(1*H*)-one **6i**. ^{1}H NMR (500 MHz, $CDCl_3$): δ 0.78 (t, *J*= 7.4 Hz, 3H), 1.51-1.58 (m, 2H), 3.66 (t, *J*= 6.9 Hz, 2H), 4.64 (s, 2H), 6.96 (d, *J*= 8.2 Hz, 1H), 7.06-7.07 (m, 2H), 7.21-7.26 (m, 1H), 7.37-7.55 (m, 5H). ^{13}C NMR (125 MHz, $CDCl_3$): δ 10.7, 21.3, 48.5, 49.0, 112.9, 123.5, 123.6, 126.0, 126.1, 127.2, 128.7, 129.6, 135.6, 138.8, 158.5.

1-Propyl-2-(2-methylphenyl)-1,4-dihydroquinazoline (2j): The ^{1}H and ^{13}C NMR signals were obtained by difference from the spectrum of the mixture of compound **2j** and 2-(2-methylphenyl)-1-propylquinazolin-4(1*H*)-one **6j**. ^{1}H NMR (500 MHz, $CDCl_3$): δ 0.76 (t, *J*= 7.3 Hz, 3H), 1.50-1.52 (m, 2H), 2.16 (s, 3H), 3.28 (bs, 1H), 3.50 (bs, 1H), 4.56-4.76 (m, 2H), 6.85 (d, *J*= 8.2 Hz, 1H), 7.02-7.04 (m, 2H), 7.16 (d, J= 7.6 Hz, 1H), 7.17-7.21 (m, 2H), 7.24-7.28 (m, 2H). ^{13}C NMR (125 MHz, $CDCl_3$): δ 10.9, 18.9, 20.9, 46.7, 48.9, 111.8, 122.8, 123.3, 125.8, 125.93, 126.00, 127.2, 128.9, 130.0, 134.5, 135.8, 138.3, 157.7.

2-Methyl-1-propylquinazolin-4(1*H*)-one (6a): Yellow oil. ^{1}H NMR (500 MHz, $CDCl_3$): δ 1.10 (t, *J*= 7.4 Hz, 3H), 1.83-1.91 (m, 2H), 2.68 (s, 3H), 4.09 (t, *J*= 8.2 Hz, 2H), 7.35 (d, *J*= 8.6 Hz, 1H), 7.42-7.45 (m, 1H), 7.71 (ddd, *J*= 8.6, 7.3, 1.6 Hz, 1H), 8.34 (dd, *J*= 7.9, 1.6 Hz, 1H). ^{13}C NMR (125 MHz, $CDCl_3$): δ 11.0, 21.6, 23.8, 48.9, 114.5, 120.4, 125.7, 128.9, 133.7, 140.3, 161.0, 168.5. HRMS (ESI) [M+H]+ m/z calcd for $C_{12}H_{15}N_2O$: 203.1179. Found: 203.1177.

2-((Ethylamino)methyl)aniline (3a): Oil (82% yield). ^{1}H NMR (500 MHz, $CDCl_3$): δ 1.13 (t, *J*= 7.1 Hz, 3H), 2.68 (c, *J*= 7.1 Hz, 2H), 3.81 (s, 2H), 6.65-6.70 (m, 2H), 7.03-7.04 (m, 1H), 7.09 (td, *J*= 7.6, 1.6 Hz, 1H). ^{13}C NMR (125 MHz, $CDCl_3$): δ 15.2, 43.6, 52.7, 115.7, 117.7, 124.2, 128.3, 129.8, 146.8.

2-((Propylamino)methyl)aniline (3b): Oil (93% yield). 1H NMR (500 MHz, $CDCl_3$): δ 0.92 (t, *J*= 7.4 Hz, 3H), 1.51-1.58 (m, 2H), 2.61 (t, *J*= 7.1 Hz, 2H), 3.82 (s, 2H), 6.65-6.70 (m, 2H), 7.04 (dd, *J*= 7.3, 0.9 Hz, 1H), 7.08-7.11 (m, 1H). ^{13}C NMR (125 MHz, $CDCl_3$): δ 11.7, 22.7, 50.8, 52.5, 115.9, 117.8, 123.3, 128.5, 130.1, 146.9.

2-((Isobutylamino)methyl)aniline (3c): Yellow oil (90% yield). ^{1}H NMR (500 MHz, $CDCl_3$): δ 0.92 (d, *J*= 6.6 Hz, 3H), 1.75 (non, *J*= 6.6 Hz, 1H), 2.45 (t, *J*= 6.6 Hz, 2H), 3.79 (s, 2H), 4.62 (bs, ex, 2H), 6.65-6.70 (m, 2H), 7.04 (dd, *J*= 7.3, 1.1 Hz, 1H), 7.08-7.11 (m, 1H). ^{13}C NMR (125 MHz, $CDCl_3$): δ 20.6, 28.3, 53.3, 57.3, 115.7, 117.6, 124.3, 128.2, 129.7, 147.0. HRMS (ESI) [M+H]+ m/z calcd for $C_{11}H_{19}N_2$: 179.1543. Found: 179.1545.

***N*-(2-Aminobenzyl)acetamide (4a):** White solid (90% yield), mp: 110-112°C. ^{1}H NMR (500 MHz, $CDCl_3$): δ 1.98 (s, 3H), 4.30 (bs, ex, 2H), 4.36 (d, *J*= 6.2 Hz, 2H), 5.84 (bs, ex, 1H), 6.65-6.69 (m, 2H), 7.02-7.03 (m, 1H), 7.11 (td, *J*= 7.7, 1.6 Hz, 1H). ^{13}C NMR (125 MHz, $CDCl_3$): δ 22.9, 40.7, 115.6, 117.6, 121.9, 129.1, 130.5, 145.4, 170.6.

***N*-(2-Aminobenzyl)propionamide (4b):** Yellow oil (93% yield).^{1}H NMR (500 MHz, $CDCl_3$): δ 1.13 (t, *J*= 7.6 Hz, 3H), 2.19 (c, *J*= 7.6 Hz, 2H), 4.30 (bs, ex, 2H), 4.34 (d, *J*= 6.2 Hz, 2H), 6.04 (bs, ex, 1H), 6.63-6.67 (m, 2H), 7.01 (dd, *J*= 7.5, 1.4 Hz, 1H), 7.07-7.11 (m, 1H). ^{13}C NMR (125 MHz, $CDCl_3$): δ 9.7, 29.5, 40.6, 115.6, 117.6, 121.9, 129.1, 130.5, 145.5, 174.2. HRMS (ESI) [M+H]+ m/z calcd for $C_{10}H_{15}N_2O$: 179.1179. Found: 179.1184.

***N*-(2-Aminobenzyl)isobutyramide (4c):** White solid (94% yield), mp: 96-98°C. ^{1}H NMR (500 MHz, $CDCl_3$): δ 1.14 (d, *J*= 6.9 Hz, 6H), 2.34 (h, *J*= 6.9 Hz, 1H), 4.23 (bs, ex, 2H), 4.35 (d, *J*= 6.2 Hz, 2H), 5.94 (bs, ex, 1H), 6.63-6.67 (m, 2H), 7.02 (dd, *J*= 7.7, 1.5 Hz, 1H), 7.09 (td, *J*= 7.7, 1.5 Hz, 1H). ^{13}C NMR (125 MHz, $CDCl_3$): δ 19.5, 35.5, 40.7, 115.5, 117.4, 121.9, 129.1, 130.5, 145.6, 177.4. HRMS (ESI) [M+H]+ m/z calcd for $C_{11}H_{17}N_2O$: 193.1335. Found: 193.1335.

***N*-(2-Aminobenzyl)pivalamide (4d):** White solid (93% yield), mp: 119-121°C. ^{1}H NMR (500 MHz, $CDCl_3$): δ 1.19 (s, 9H), 4.31 (bs, ex, 2H), 4.39 (d, *J*= 6.2 Hz, 2H), 5.94 (bs, ex, 1H), 6.64-6.68 (m, 2H), 7.05 (dd, *J*= 7.5, 1.5 Hz, 1H), 7.09-7.12 (m, 1H). ^{13}C NMR (125 MHz, $CDCl_3$): δ 27.5, 38.8, 41.0, 115.5, 117.4, 121.9, 129.1, 130.5, 145.7, 178.9. HRMS (ESI) [M+H]+ m/z calcd for $C_{12}H_{19}N_2O$: 207.1492. Found: 207.1488.

***N*-(2-Aminobenzyl)benzamide (4e):** White solid (94% yield), mp: 107-109°C. ^{1}H NMR (500 MHz, $CDCl_3$): δ 4.13 (bs, ex, 2H), 4.58 (d, *J*= 6.2 Hz, 2H), 6.55 (bs, ex, 1H), 6.68-6.72 (m, 2H), 7.11-7.14 (m, 2H), 7.39-7.42 (m, 2H), 7.47-7.50 (m, 1H), 7.75 (d, *J*= 8.0 Hz, 2H). ^{13}C NMR (125 MHz, $CDCl_3$): δ 41.3, 115.9, 117.9, 121.8, 126.9, 128.6, 129.3, 131.6, 133.9, 136.6, 145.4, 167.8.

***N*-(2-Aminobenzyl)-2-methylbenzamide (4f):** White solid (83% yield), mp: 114-116°C. ^{1}H NMR (500 MHz, $CDCl_3$): δ 2.43 (s, 3H), 4.31 (bs, ex, 2H), 4.55 (d, *J*= 6.2 Hz, 2H), 6.11 (bs, ex, 1H), 6.67-6.70 (m, 2H), 7.08 (dd, *J*= 7.8, 1.6 Hz, 1H), 7.12 (td, *J*= 7.6, 1.6, 1H), 7.16-7.21 (m, 2H), 7.29 (td, *J*= 7.6, 1.4 Hz, 1H), 7.33 (dd, *J*= 7.6, 1.4 Hz, 1H). ^{13}C NMR (125 MHz, $CDCl_3$): δ 19.8, 41.1, 115.7, 117.8, 121.7, 125.7, 126.7, 129.3, 130.0, 130.6, 131.0, 135.8, 136.2, 145.5, 170.4. HRMS (ESI) [M+H]+ m/z calcd for $C_{15}H_{17}N_2O$: 241.1335. Found: 241.1331.

***N*-(2-Aminobenzyl)-2-fluorobenzamide (4g):** Yellow solid (70% yield), mp: 95-97°C. ^{1}H NMR (500 MHz, $CDCl_3$): δ 4.25 (bs, ex, 2H), 4.61 (dd, *J*= 6.2, 1.4 Hz, 2H), 6.67-6.72 (m, 2H), 7.04-7.14 (m, 4H), 7.23-7.26 (m, 1H), 7.43-7.47 (m, 1H), 8.09 (td, *J*= 7.9, 1.8, 1H). ^{13}C NMR (125 MHz, $CDCl_3$): δ 41.2, 115.8, 116.0 (d, *J*= 24.5 Hz), 117.9, 120.6 (d, *J*= 10.9 Hz), 121.7, 124.7 (d, *J*= 3.6 Hz), 129.2, 130.6, 132.0 (d, *J*= 2.7 Hz), 133.4 (d,

J= 9.1 Hz), 145.5, 160.5 (d, J= 248.0 Hz), 163.7 (d, *J*= 3.6 Hz). HRMS (ESI) [M+H]+ m/z calcd for $C_{14}H_{14}FN_2O$: 245.1085. Found: 245.1091.

***N*-(2-Aminobenzyl)-*N*-ethylacetamide (4h):** White solid (81% yield), mp: 75-77°C. ^{1}H NMR (500 MHz, $CDCl_3$): δ 1.14 (t, *J*= 7.2 Hz, 3H), 2.14 (s, 3H), 3.26 (c, J= 7.2 Hz, 2H), 4.51 (s, 2H), 4.57 (bs, ex, 2H), 6.62-6.65 (m, 2H), 7.01-7.03 (m, 1H), 7.08-7.12 (m, 1H). ^{13}C NMR (125 MHz, $CDCl_3$): δ 13.0, 21.2, 41.4, 45.1, 115.3, 116.9, 120.0, 129.3, 131.7, 146.2, 170.7. HRMS (ESI) [M+H]+ m/z calcd for $C_{11}H_{17}N_2O$: 193.1335. Found: 193.1327.

***N*-(2-Aminobenzyl)-*N*-ethylpropionamide (4i):** Yellow solid (80% yield), mp: 75-77°C. ^{1}H NMR (500 MHz, $CDCl_3$): δ 1.14 (t, *J*= 7.1 Hz, 3H), 1.17 (t, *J*= 7.4 Hz, 3H), 2.38 (c, *J*= 7.4 Hz, 2H), 3.26 (c, *J*= 7.1 Hz, 2H), 4.52 (s, 2H), 4.59 (bs, ex, 2H), 6.62-6.65 (m, 2H), 7.02 (d, *J*= 7.5 Hz, 1H), 7.08-7.11 (m, 1H). ^{13}C NMR (125 MHz, $CDCl_3$): δ 9.7, 13.1, 26.1, 40.4, 45.3, 115.3, 116.8, 120.2, 129.2, 131.7, 146.2, 173.9. HRMS (ESI) [M+H]+ m/z calcd for $C_{12}H_{19}N_2O$: 207.1492. Found: 207.1498.

***N*-(2-Aminobenzyl)-*N*-propylpropionamide (4j):** White solid (72% yield), mp: 77-79°C. ^{1}H NMR (500 MHz, $CDCl_3$): δ 0.90 (t, *J*= 7.4 Hz, 3H), 1.16 (t, *J*= 7.4 Hz, 3H), 1.54-1.62 (m, 2H), 2.37 (c, *J*= 7.4 Hz, 2H), 3.13-3.16 (m, 2H), 4.53 (s, 2H), 4.89 (bs, ex, 2H), 6.65-6.70 (m, 2H), 7.02 (dd, *J*= 7.3, 1.1 Hz, 1H), 7.09-7.13 (m, 1H). ^{13}C NMR (125 MHz, $CDCl_3$): δ 9.7, 11.2, 21.1, 26.2, 45.5, 47.6, 115.2, 116.8, 120.1, 129.2, 131.7, 146.2, 174.1. HRMS (ESI) [M+H]+ m/z calcd for $C_{13}H_{21}N_2O$: 221.1648. Found: 221.1654.

***N*-(2-Aminobenzyl)-*N*-isobutylpropionamide (4k):** White solid (79% yield), mp: 83-85°C. ^{1}H NMR (500 MHz, $CDCl_3$): δ 0.93 (d, *J*= 6.6 Hz, 6H), 1.16 (t, *J*= 7.4 Hz, 3H), 2.04-2.12 (m, 1H), 2.38 (c, *J*= 7.4 Hz, 2H), 3.00 (d, *J*= 7.8 Hz, 2H), 4.52 (bs, ex, 2H), 4.58 (s, 2H), 6.61-6.64 (m, 2H), 6.97-6.99 (m, 1H), 7.09 (td, *J*= 7.6, 1.4 Hz, 1H). ^{13}C NMR (125 MHz, $CDCl_3$): δ 9.7, 20.1, 26.5, 26.7, 45.2, 52.5, 115.3, 116.8, 119.9, 129.1, 131.8, 146.2, 174.6. HRMS (ESI) [M+H]+ m/z calcd for $C_{14}H_{23}N_2O$: 235.1805. Found: 235.1799.

***N*-(2-(Propylamino)benzyl)acetamide (5a):** White solid (81% yield), mp: 91-93°C. ^{1}H NMR (500 MHz, $CDCl_3$): δ 1.00 (t, *J*= 7.4 Hz, 3H), 1.64-1.71 (m, 2H), 1.97 (s, 3H), 3.07 (t, *J*= 7.1 Hz, 2H), 4.35 (d, *J*= 6.2 Hz, 2H), 4.89 (bs, ex, 1H), 5.83 (bs, ex, 1H), 6.58-6.61 (m, 2H), 7.02 (dd, *J*= 7.3, 1.4 Hz, 1H), 7.17-7.21 (m, 1H). ^{13}C NMR (125 MHz, $CDCl_3$): δ 11.7, 22.3, 23.1, 41.0, 45.5, 110.1, 115.5, 121.3, 129.5, 130.4, 146.8, 170.4. HRMS (ESI) [M+H]+ m/z calcd for $C_{12}H_{19}N_2O$: 207.1492. Found: 207.1486.

***N*-(2-(Allylamino)benzyl)acetamide (5b):** White solid (68% yield). ^{1}H NMR (500 MHz, $CDCl_3$): δ 1.97 (s, 3H), 3.80 (dt, *J*= 5.3, 1.6 Hz, 2H), 4.37 (d, *J*= 6.4 Hz, 2H), 5.14-5.17 (m, 1H), 5.24-5.29 (m, 1H), 5.83 (bs, ex, 1H), 5.94 (ddt, *J*= 17.2, 10.3, 5.3 Hz, 1H), 6.60-6.64 (m, 2H), 7.04 (dd, *J*= 7.4, 1.5 Hz, 1H), 7.17-7.20 (m, 1H). ^{13}C NMR (125 MHz, $CDCl_3$): δ 23.1, 41.0, 46.0, 110.6, 115.8, 116.1, 121.7, 129.4, 130.4, 135.2, 146.3, 170.5. HRMS (ESI) [M+H]+ m/z calcd for $C_{12}H_{17}N_2O$: 205.1335. Found: 205.1335.

***N*-(2-(Benzylamino)benzyl)acetamide (5c):** White solid (64% yield), mp: 124-128°C. ^{1}H NMR (500 MHz, $CDCl_3$): δ 1.89 (s, 3H), 4.33-4.34 (m, 4H), 5.48 (bs, ex, 1H), 5.83 (bs, ex, 1H), 6.51 (d, *J*= 8.2 Hz, 1H), 6.55 (td, *J*= 7.3, 0.9 Hz, 1H), 6.98 (dd, *J*= 7.3, 1.6 Hz, 1H), 7.04-7.08 (m, 1H), 7.13-7.28 (m, 5H). ^{13}C NMR (125 MHz, $CDCl_3$): δ 23.1, 41.0, 47.5, 110.8, 116.2, 121.8, 126.9, 127.0, 128.4, 129.4, 130.4, 139.5, 146.3, 170.5. HRMS (ESI) [M+H]+ m/z calcd for $C_{16}H_{19}N_2O$: 255.1492. Found: 255.1497.

***N*-(2-(Isopropylamino)benzyl)acetamide (5d):** Yellow solid (60% yield), mp: 109-110°C. ^{1}H NMR (500 MHz, $CDCl_3$): δ 1.22 (d, *J*= 6.2 Hz, 6H), 1.97 (s, 3H), 3.64 (h, *J*= 6.2 Hz, 1H), 4.34 (d, J= 6.4 Hz, 2H), 4.84 (bs, ex, 1H), 5.75 (bs, ex, 1H), 6.58 (td, J= 7.3, 0.9 Hz, 1H), 6.63 (d, *J*= 8.3 Hz, 1H), 7.02 (dd, J= 7.3, 1.6 Hz, 1H), 7.19 (ddd, J= 8.3, 7.3, 1.6 Hz, 1H). ^{13}C NMR (125 MHz, $CDCl_3$): δ 22.6, 23.1, 41.1, 43.8, 110.8, 115.4, 121.6, 129.4, 130.6, 145.7, 170.3. HRMS (ESI) [M+H]+ m/z calcd for $C_{12}H_{19}N_2O$: 207.1492. Found: 207.1499.

***N*-(2-(Propylamino)benzyl)isobutyramide (5e):** Yellow solid (67% yield), mp: 90-92°C. ^{1}H NMR (500 MHz, $CDCl_3$): δ 1.00 (t, *J*= 7.3 Hz, 3H), 1.15 (d, *J*= 6.9 Hz, 6H), 1.63-1.70 (m, 2H), 2.32 (h, *J*= 6.9 Hz, 1H), 3.07 (t, *J*= 7.1 Hz, 2H), 4.38 (d, *J*= 6.4 Hz, 2H), 4.88 (bs, ex, 1H), 5.71 (bs, ex, 1H), 6.58-6.71 (m, 2H), 7.03 (d, *J*= 7.3 Hz, 1H), 7.19 (t, *J*= 7.8 Hz, 1H). ^{13}C NMR (125 MHz, $CDCl_3$): δ 11.7, 19.5, 22.4, 35.7, 41.0, 45.5, 109.9, 115.4, 121.3, 129.4, 130.4, 146.9, 177.2. HRMS (ESI) [M+H]+ m/z calcd for $C_{14}H_{23}N_2O$: 235.1805. Found: 235.1800.

***N*-(2-(Propylamino)benzyl)pivalamide (5f):** Yellow oil (69% yield). ^{1}H NMR (500 MHz, $CDCl_3$): δ 1.00 (t, *J*= 7.3 Hz, 3H), 1.19 (s, 9H), 1.62-1.69 (m, 2H), 3.07 (t, *J*= 7.2 Hz, 2H), 4.39 (d, *J*= 6.2 Hz, 2H), 4.85 (bs, ex, 1H), 5.85 (bs, ex, 1H), 6.58-6.61 (m, 2H), 7.05 (dd, *J*= 7.7, 1.7 Hz, 1H), 7.18-7.21 (m, 1H). ^{13}C NMR (125 MHz, $CDCl_3$): δ 11.8, 22.4, 27.5, 38.7, 41.3, 45.4, 109.8, 115.3, 121.3, 129.4, 130.5, 147.0, 178.7. HRMS (ESI) [M+H]+ m/z calcd for $C_{15}H_{25}N_2O$: 249.1961. Found: 249.1960.

***N*-(2-(Benzylamino)benzyl)pivalamide (5g):** Yellow solid (60% yield), mp: 103-107°C. ^{1}H NMR (300 MHz, $CDCl_3$): δ 1.14 (s, 9H), 4.36 (s, 2H), 4.42 (d, *J*= 6.2 Hz, 2H), 5.54 (bs, ex, 1H), 5.90 (bs, ex, 1H), 6.50 (d, *J*= 8.2 Hz, 1H), 6.59 (td, *J*= 7.4, 1.1 Hz, 1H), 7.03-7.12 (m, 2H), 7.19-7.35 (m, 5H). ^{13}C NMR (75 MHz, $CDCl_3$): δ 27.5, 38.7, 41.4, 47.4, 110.6, 116.0, 121.7, 126.8, 127.0, 128.4, 129.4, 130.5, 139.5, 146.5, 178.9. HRMS (ESI) [M+H]+ m/z calcd for $C_{19}H_{25}N_2O$: 297.1961. Found: 297.1957.

***N*-(2-(Isopropylamino)benzyl)pivalamide (5h):** Yellow oil (75% yield). ^{1}H NMR (600 MHz, $CDCl_3$): δ 1.19 (s, 9H), 1.21 (d, *J*= 6.2 Hz, 6H), 3.63 (h, *J*= 6.2 Hz, 1H), 4.38 (d, *J*= 6.2 Hz, 2H), 4.75 (bs, ex, 1H), 5.81 (bs, ex, 1H), 6.56-6.59 (m, 1H), 6.61 (d, *J*= 8.4 Hz, 1H), 7.05 (dd, *J*= 7.3, 1.5 Hz, 1H), 7.17-7.20 (m, 1H). ^{13}C NMR (150 MHz, $CDCl_3$): δ 22.6, 27.5, 38.7, 41.4, 43.6, 110.4, 115.1, 121.5, 129.4, 130.7, 145.9, 178.5. HRMS (ESI) [M+H]+ m/z calcd for $C_{15}H_{25}N_2O$: 249.1961. Found: 249.1955.

***N*-(2-(Propylamino)benzyl)benzamide (5i):** Yellow oil (55% yield). ^{1}H NMR (500 MHz, $CDCl_3$): δ 0.99 (t, *J*= 7.4 Hz, 3H), 1.64-1.72 (m, 2H), 3.09 (t, *J*= 7.1 Hz, 2H), 4.59 (d, *J*= 6.2 Hz, 2H), 4.96 (bs, ex, 1H), 6.48 (bs, ex, 1H), 6.61-6.64 (m, 2H), 7.11 (dd, *J*= 7.6, 1.6 Hz, 1H), 7.20-7.23 (m, 1H), 7.38-7.42 (m, 2H), 7.47-7.50 (m, 1H), 7.74-7.76 (m, 2H). ^{13}C NMR (125 MHz, $CDCl_3$): δ 11.7, 22.3, 41.6, 45.5, 110.1, 115.5, 121.1, 126.9, 128.5, 129.5, 130.5, 131.5, 134.0, 146.9, 167.7. HRMS (ESI) [M+H]+ m/z calcd for $C_{17}H_{21}N_2O$: 269.1648. Found: 269.1645.

***N*-(2-(Propylamino)benzyl)-2-methylbenzamide (5j):** Yellow solid (71% yield), mp: 99-101°C. ^{1}H NMR (500 MHz, $CDCl_3$): δ 1.02 (t, *J*= 7.4 Hz, 3H), 1.68-1.75 (m, 2H), 2.43 (s, 3H), 3.12 (t, *J*= 7.2 Hz, 2H), 4.56 (d, *J*= 6.2 Hz, 2H), 4.97 (bs, ex, 1H), 5.98 (bs, ex, 1H), 6.61 (td, *J*= 7.3, 0.9 Hz, 1H), 6.64 (d, *J*= 8.2 Hz, 1H), 7.08 (dd, *J*= 7.3, 1.6, 1H), 7.14-7.23 (m, 3H), 7.27-7.32 (m, 2H). ^{13}C NMR (125 MHz, $CDCl_3$): δ 11.8, 19.7, 22.5, 41.3, 45.6, 110.1, 115.6, 121.1, 125.7, 126.6, 129.6, 130.0, 130.5, 131.0, 136.0, 150.4, 146.9, 170.4. HRMS (ESI) [M+H]+ m/z calcd for $C_{18}H_{23}N_2O$: 283.1805. Found: 283.1812.

4. Copies of ^{1}H and ^{13}C NMR Spectra of compounds **1-6**

^{1}H NMR (500 MHz, $CDCl_3$) spectrum of compound **1a**

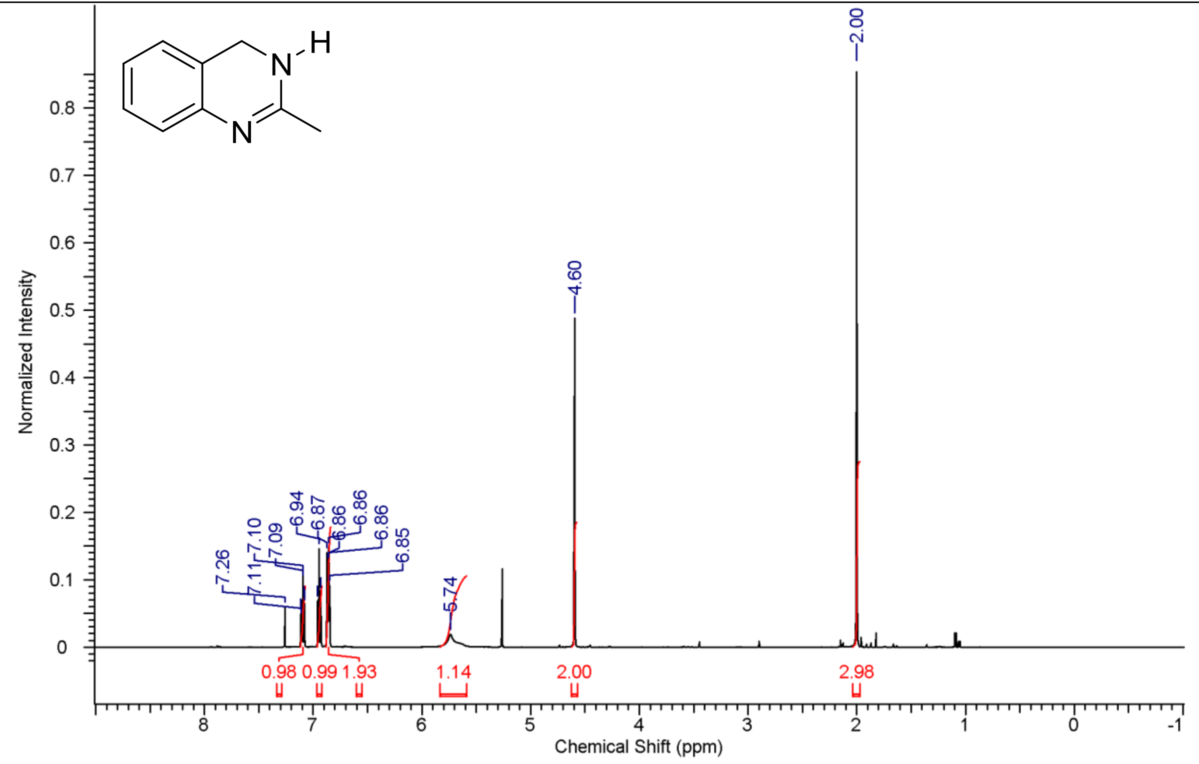

^{13}C NMR (125 MHz, $CDCl_3$) spectrum of compound **1a**

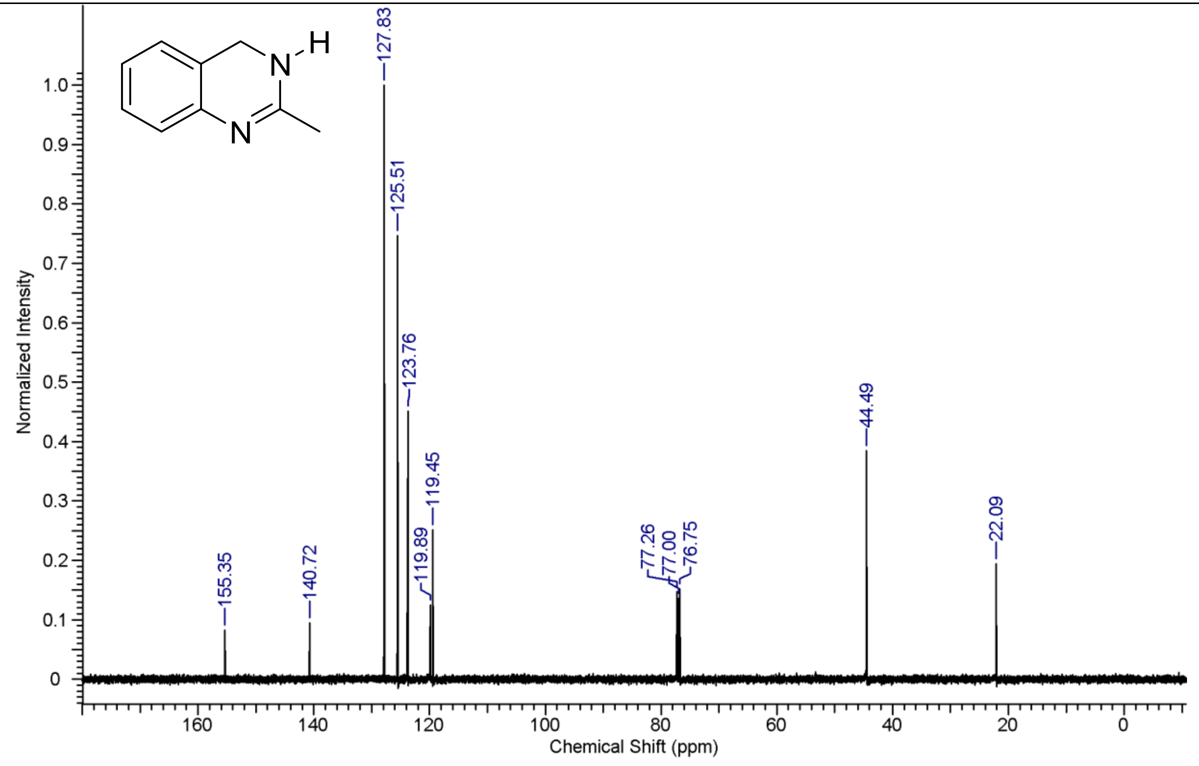

^{1}H NMR (500 MHz, $CDCl_3$) spectrum of compound **1b**

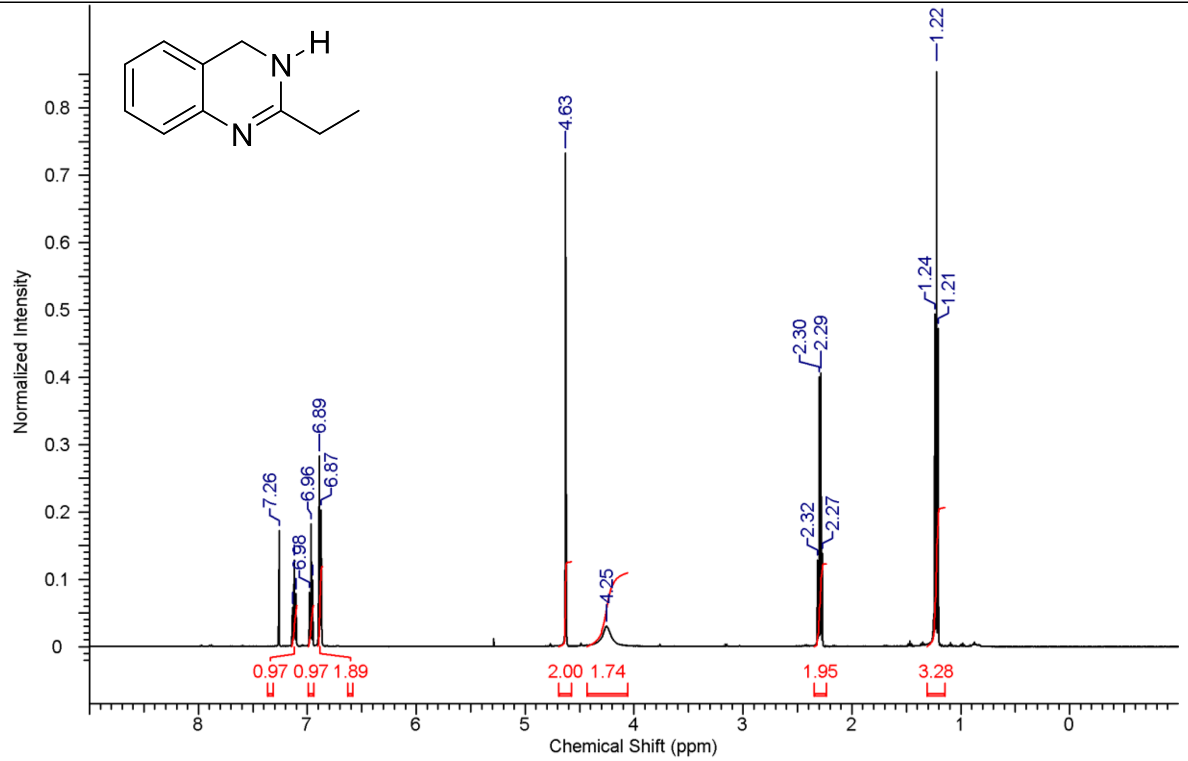

^{13}C NMR (125 MHz, $CDCl_3$) spectrum of compound **1b**

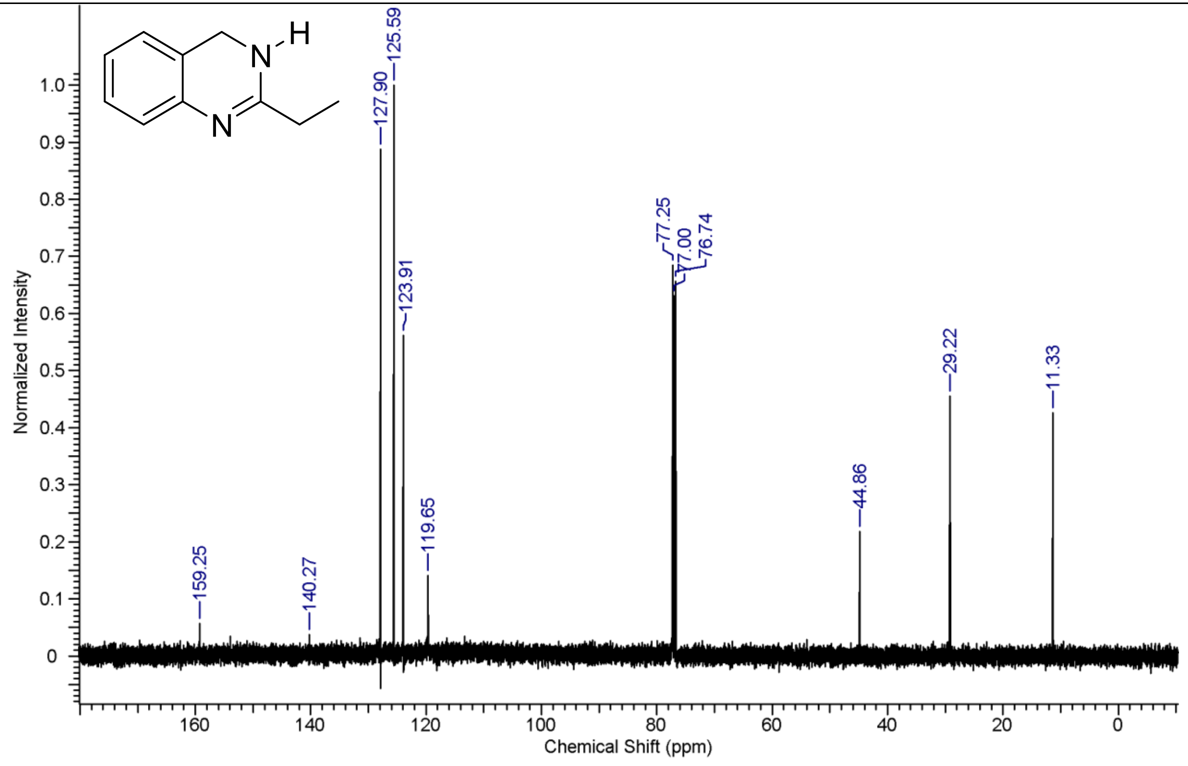

^{1}H NMR (500 MHz, $CDCl_3$) spectrum of compound **1c**

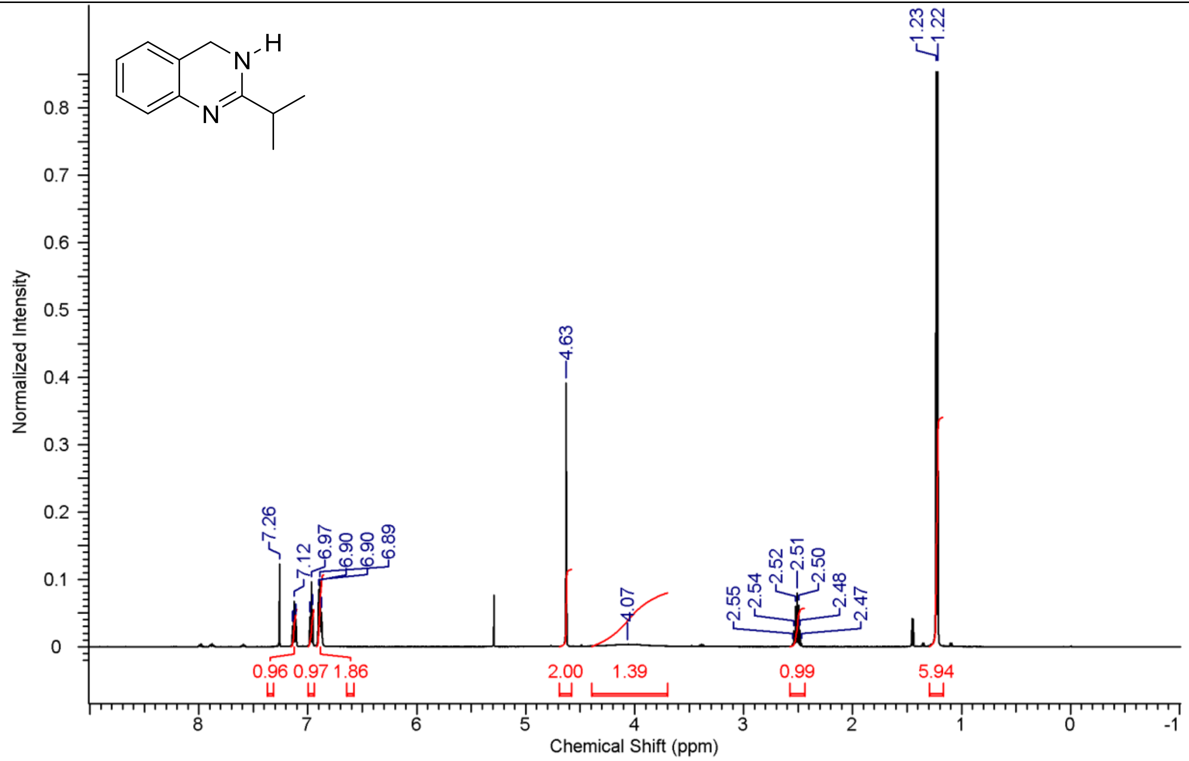

^{13}C NMR (125 MHz, $CDCl_3$) spectrum of compound **1c**

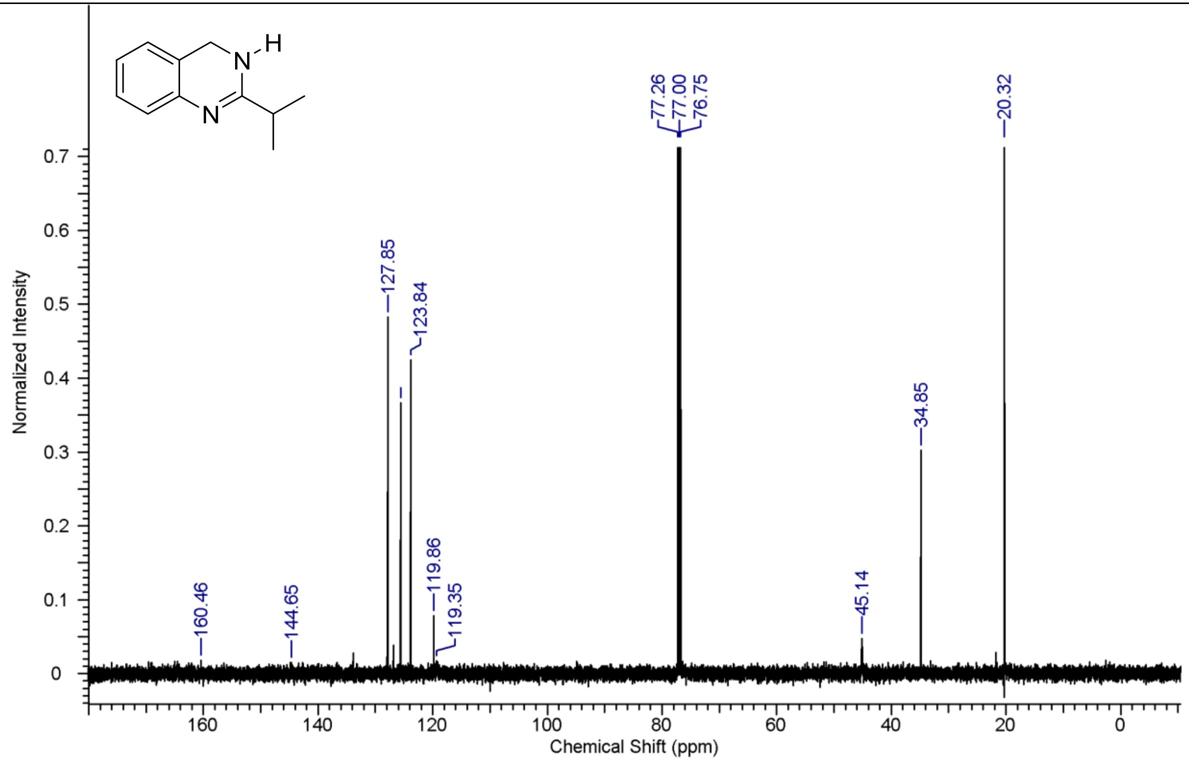

^{1}H NMR (500 MHz, $CDCl_3$) spectrum of compound **1d**

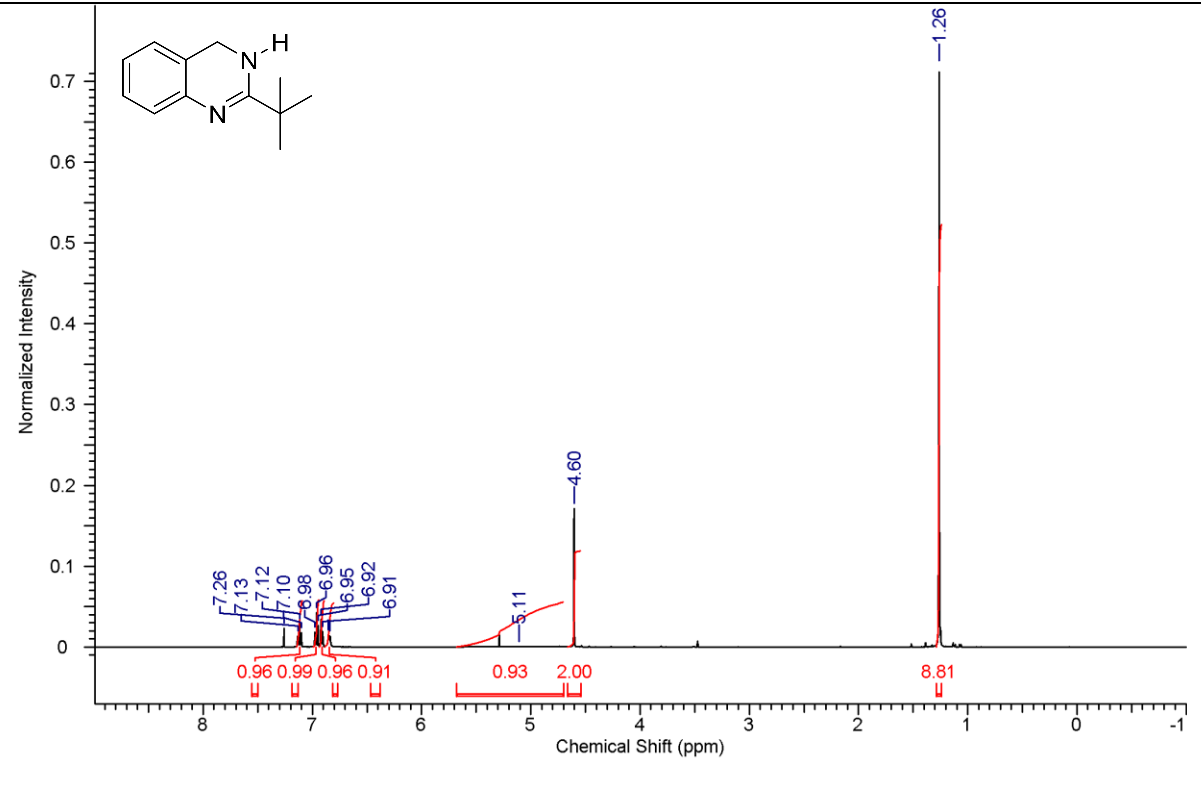

^{13}C NMR (125 MHz, $CDCl_3$) spectrum of compound **1d**

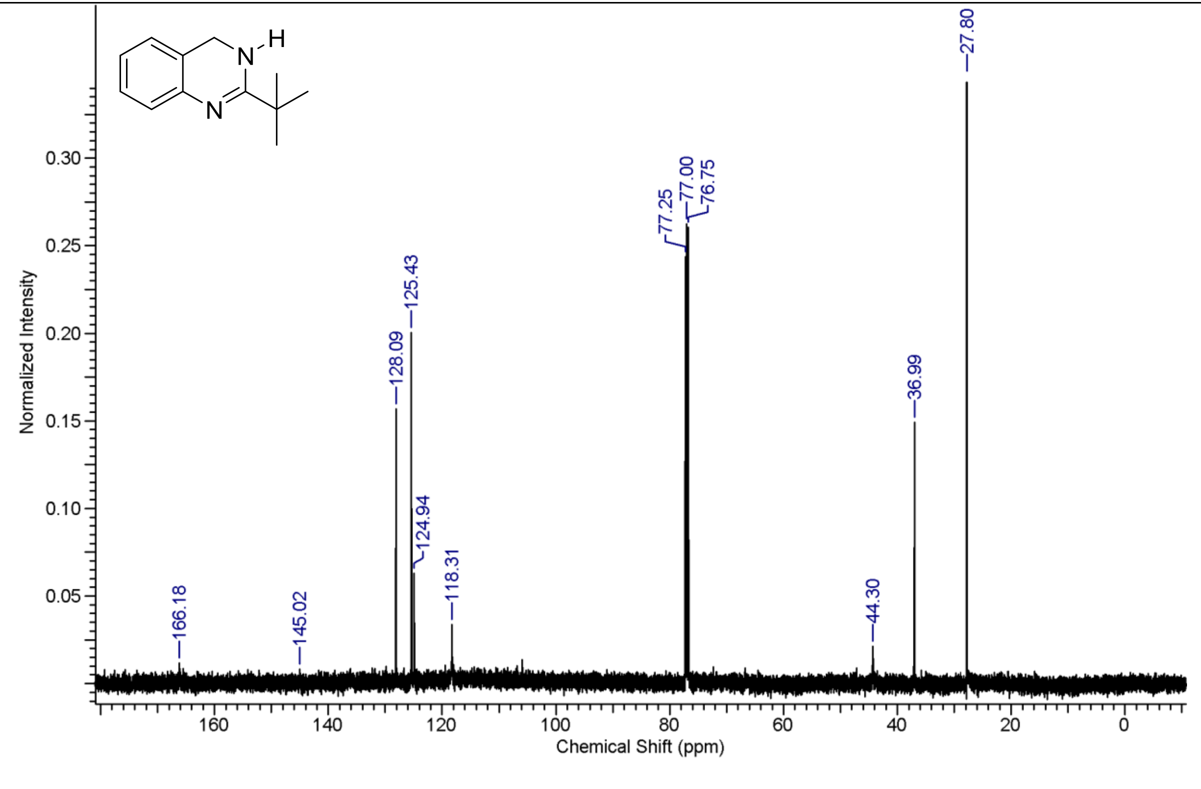

^{1}H NMR (500 MHz, $CDCl_3$) spectrum of compound **1e**

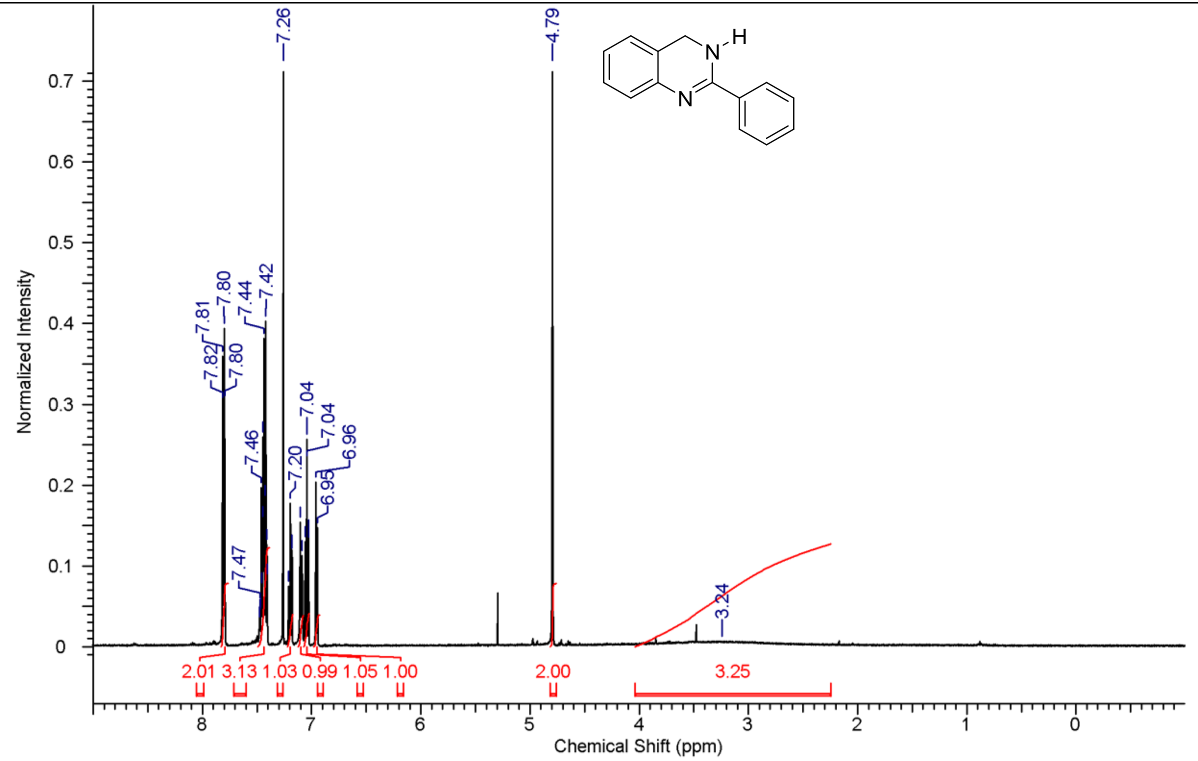

^{13}C NMR (125 MHz, $CDCl_3$) spectrum of compound **1e**

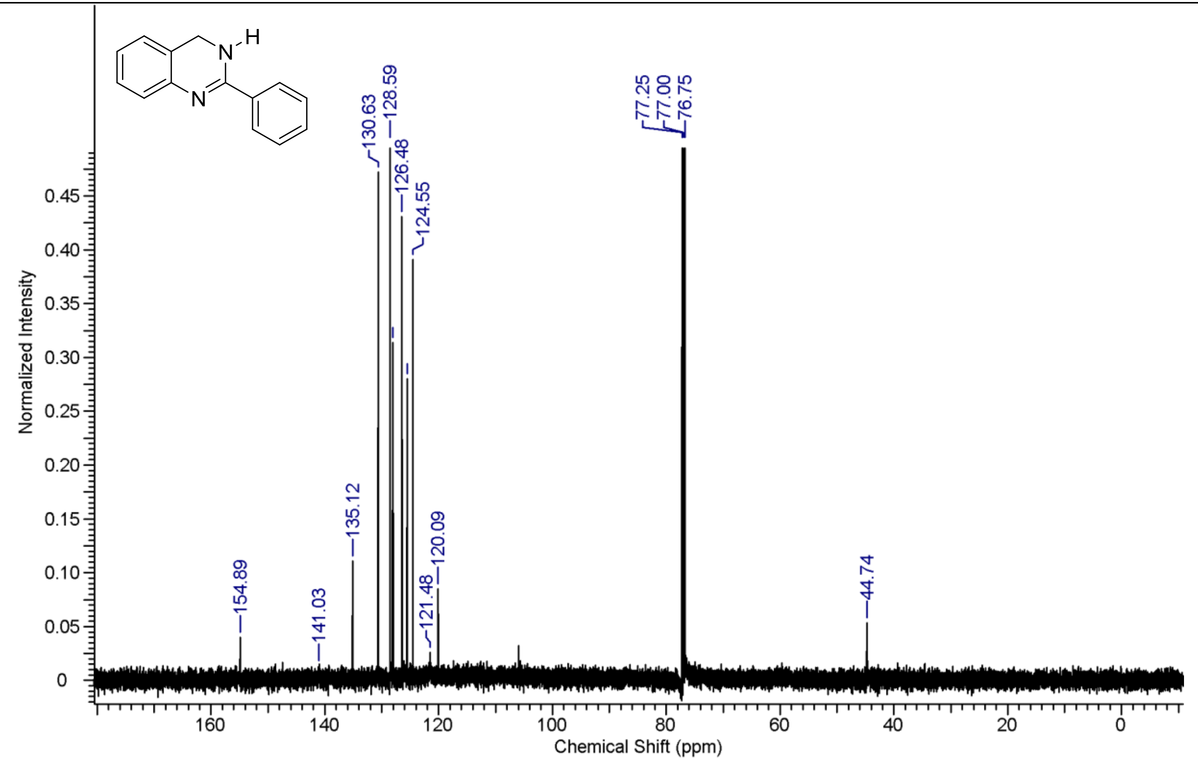

^{1}H NMR (600 MHz, $CDCl_3$) spectrum of compound **1f**

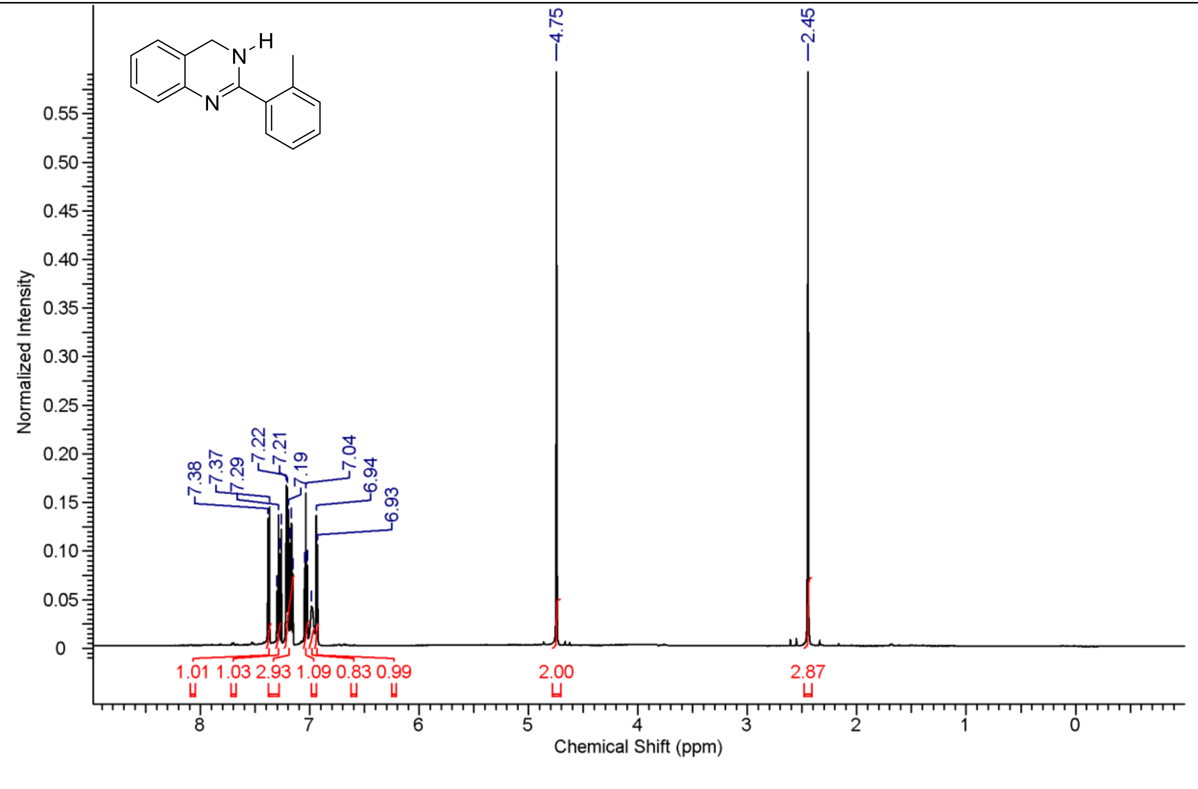

^{13}C NMR (150 MHz, $CDCl_3$) spectrum of compound **1f**

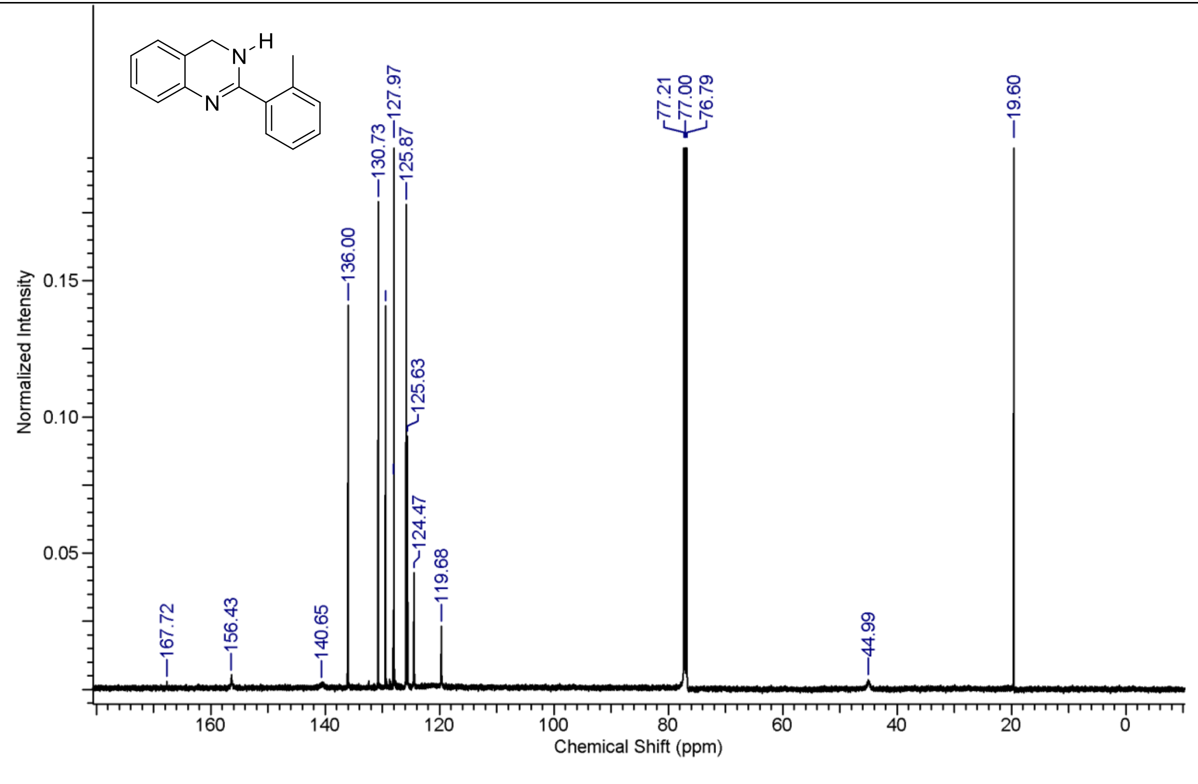

^{1}H NMR (500 MHz, $CDCl_3$) spectrum of compound **1g**

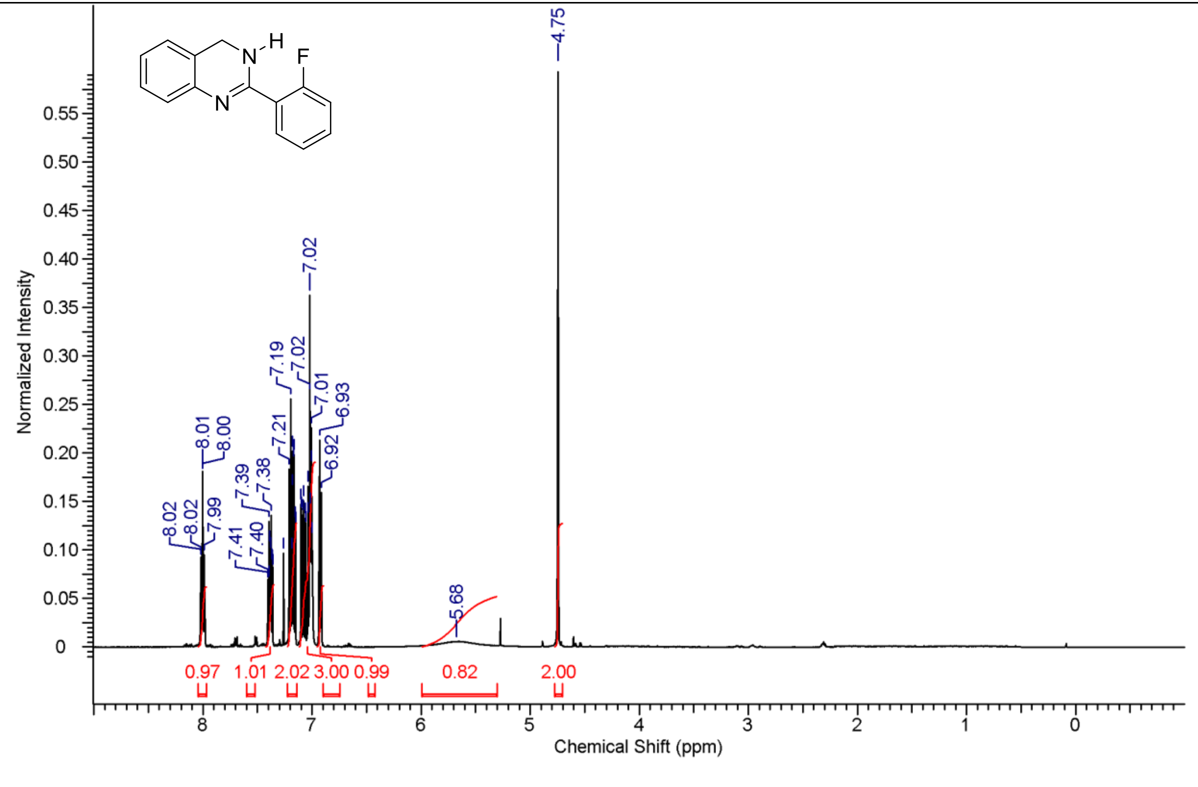

^{13}C NMR (125 MHz, $CDCl_3$) spectrum of compound **1g**

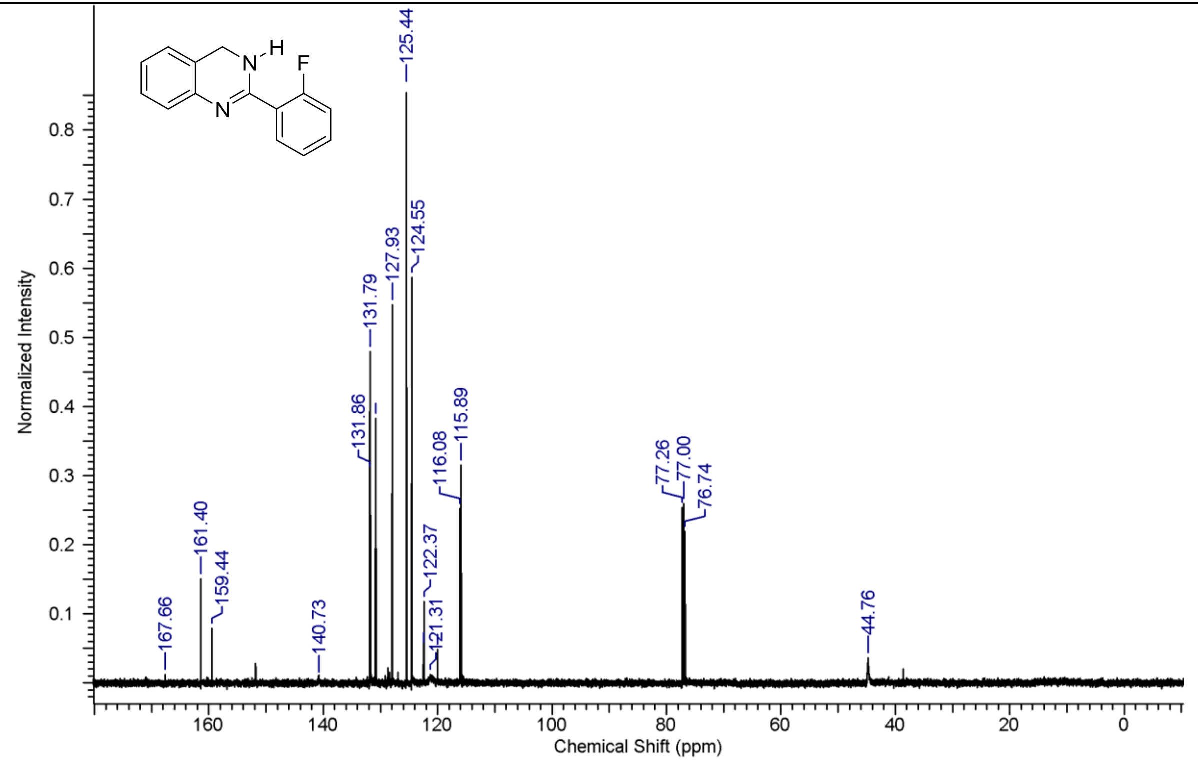

^{1}H NMR (500 MHz, $CDCl_3$) spectrum of compound **1h**

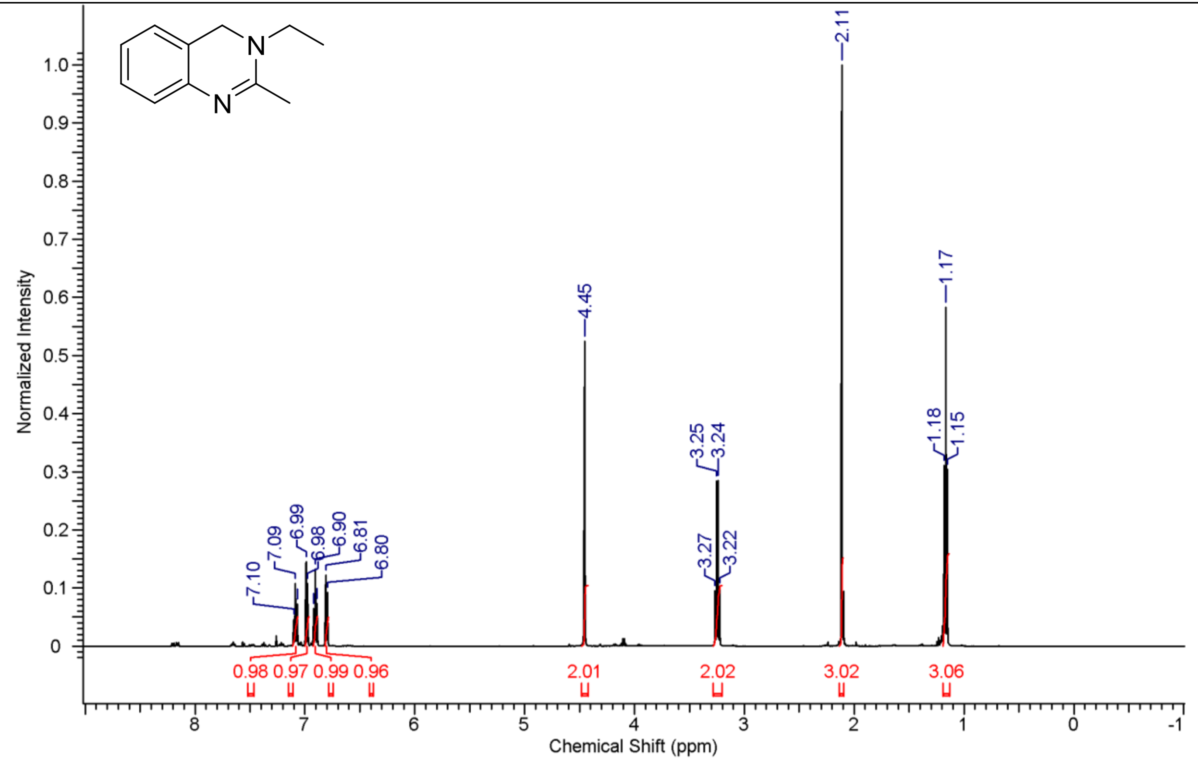

^{13}C NMR (125 MHz, $CDCl_3$) spectrum of compound **1h**

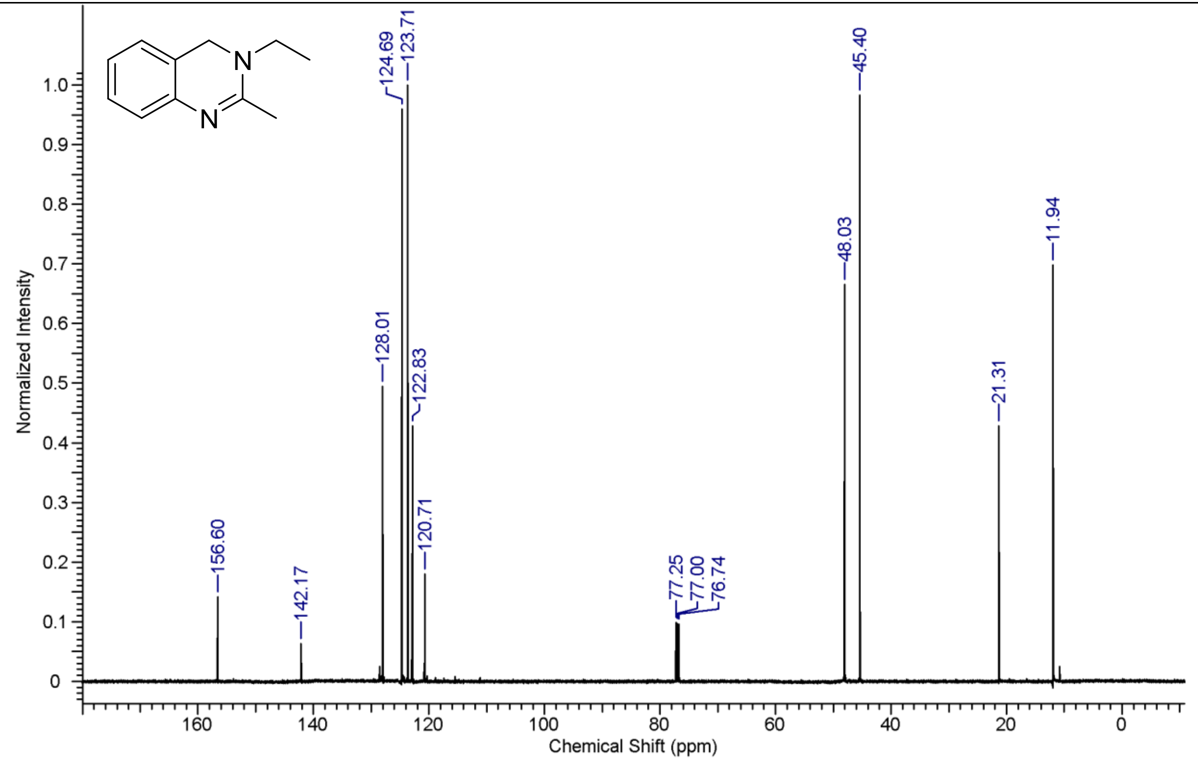

^{1}H NMR (500 MHz, $CDCl_3$) spectrum of compound **1i**

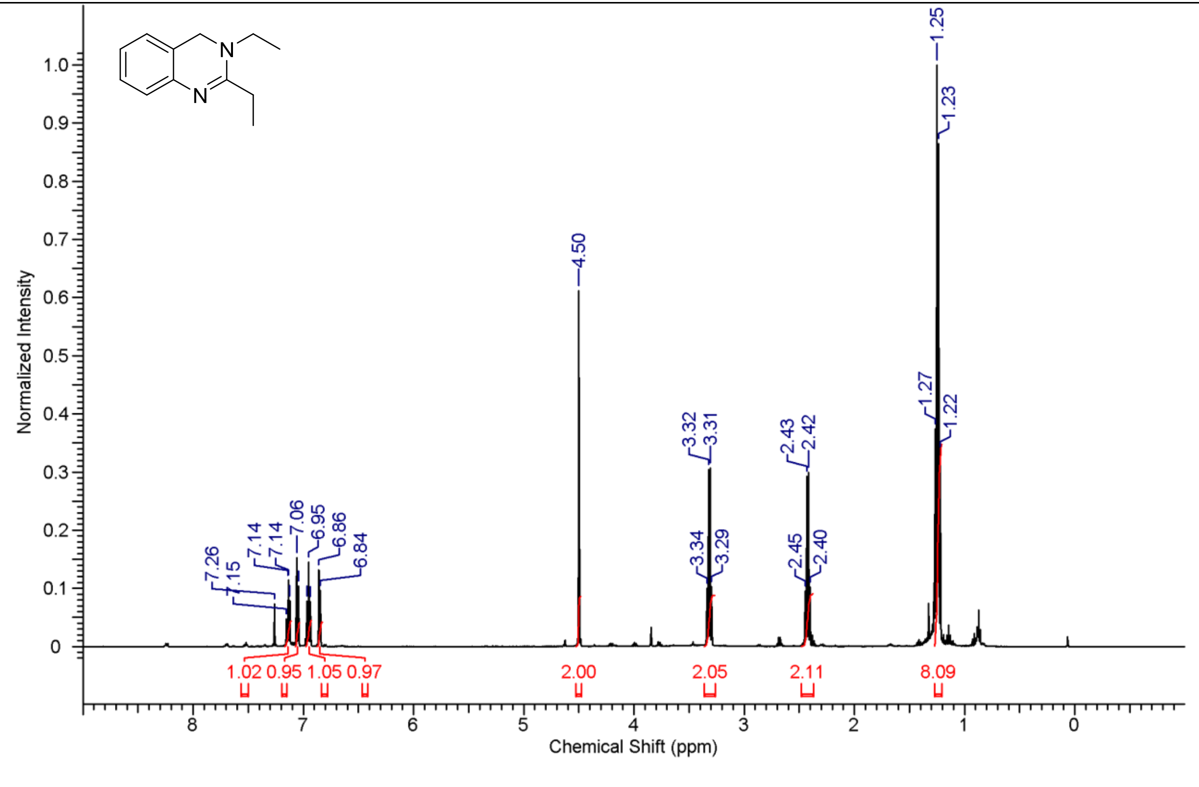

^{13}C NMR (125 MHz, $CDCl_3$) spectrum of compound **1i**

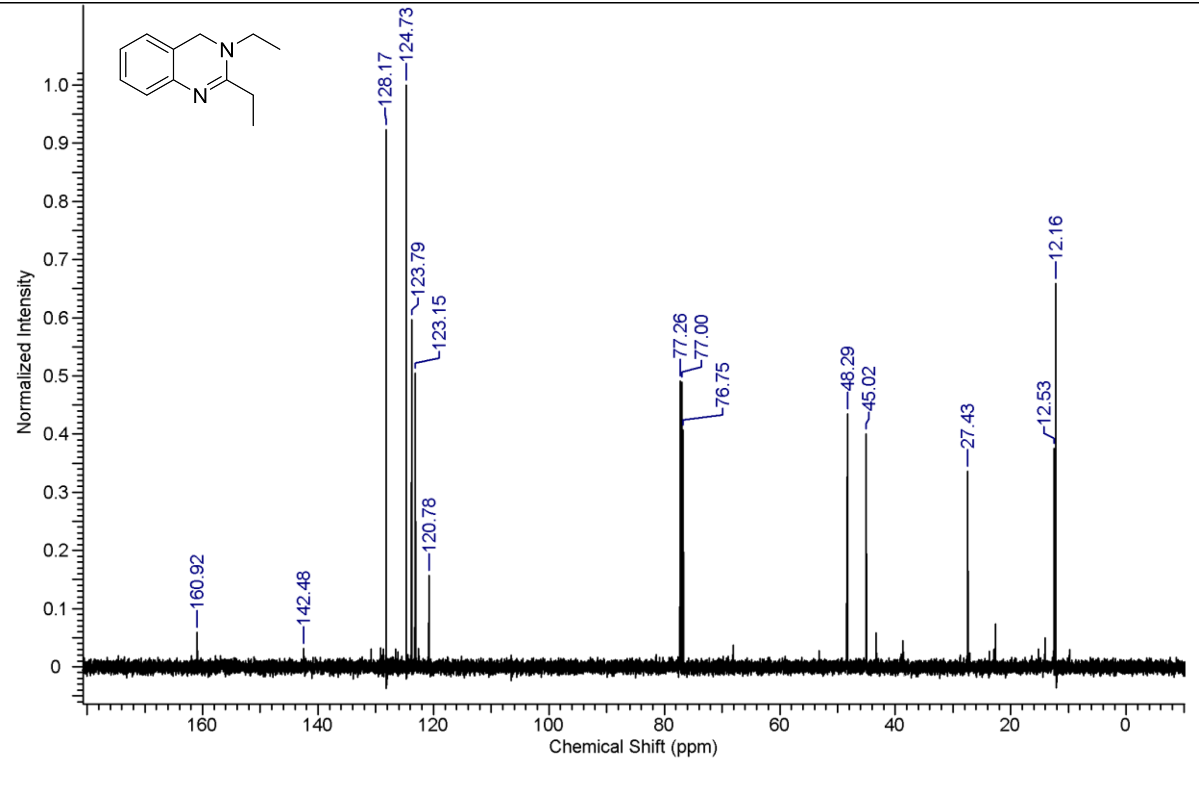

^{1}H NMR (500 MHz, $CDCl_3$) spectrum of compound **1j**

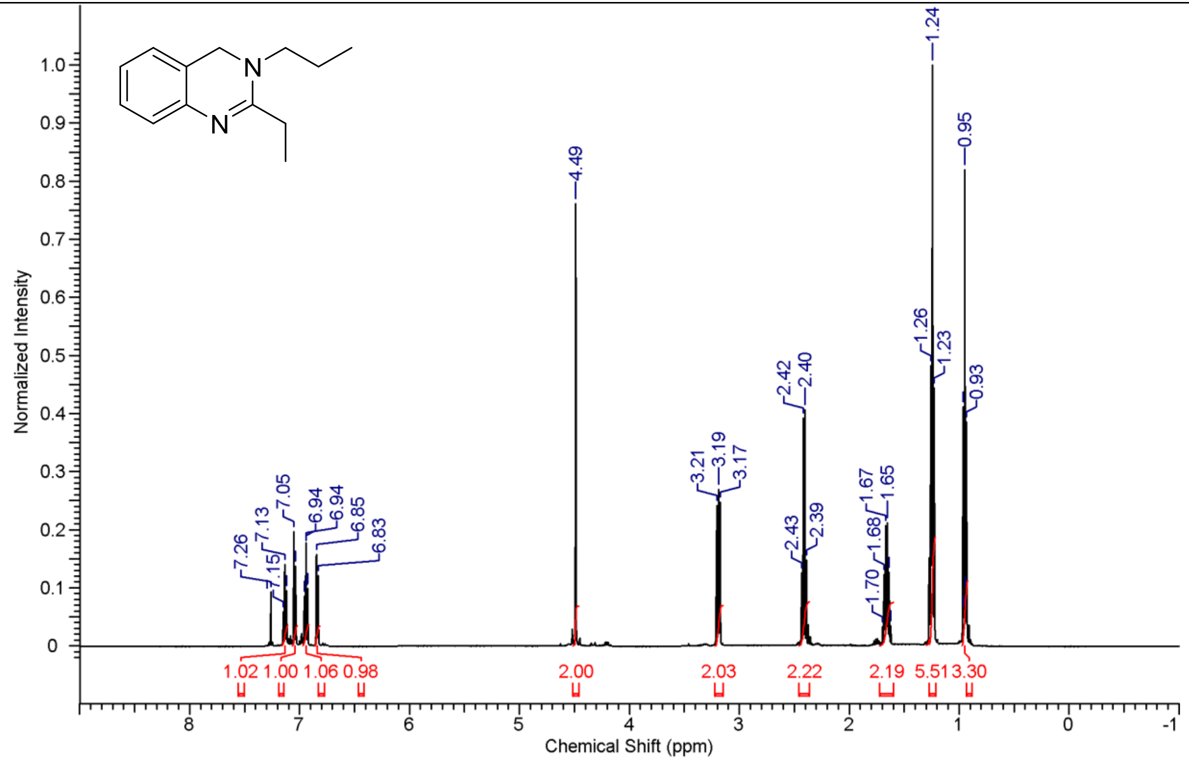

^{13}C NMR (125 MHz, $CDCl_3$) spectrum of compound **1j**

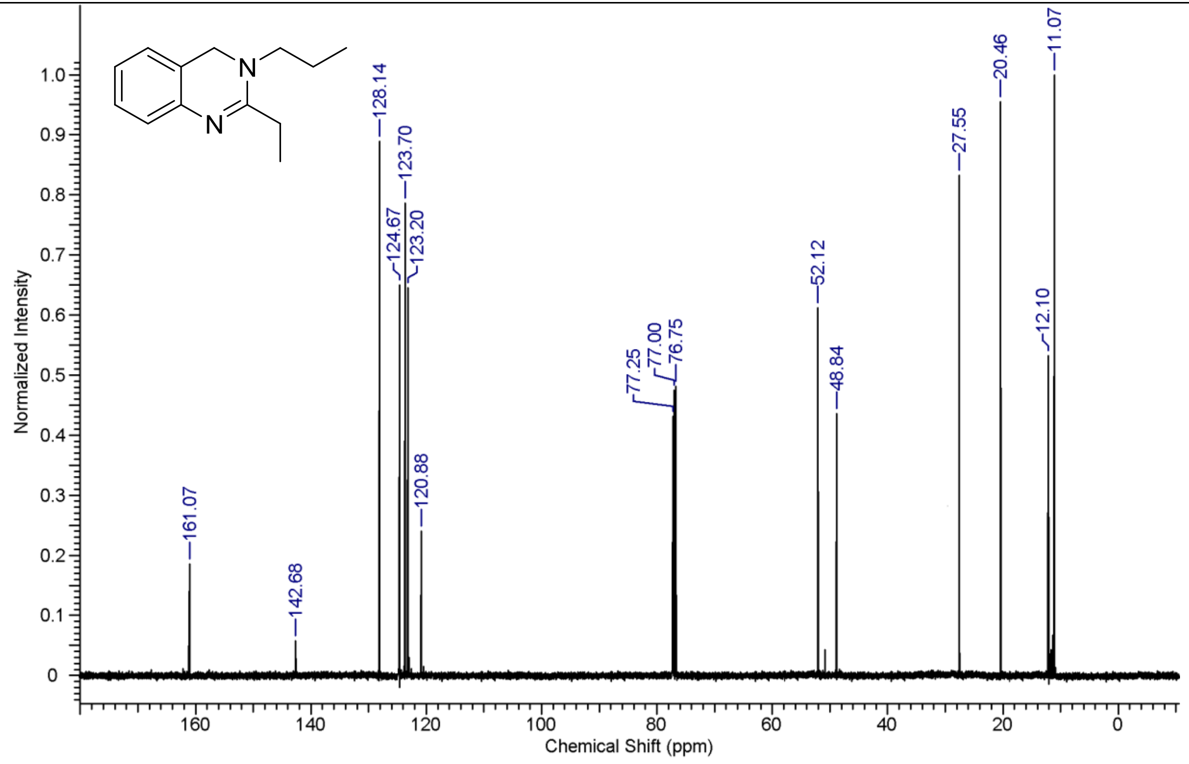

^{1}H NMR (500 MHz, $CDCl_3$) spectrum of compound **1k**

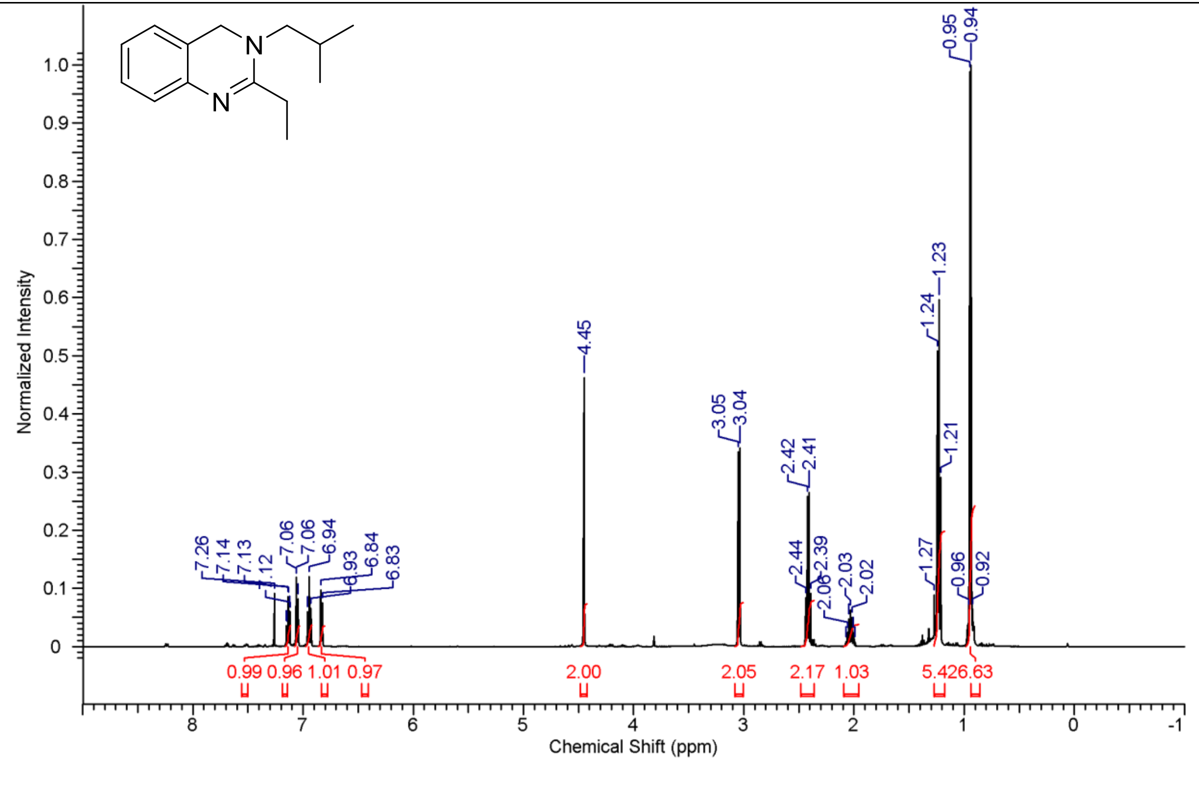

^{13}C NMR (125 MHz, $CDCl_3$) spectrum of compound **1k**

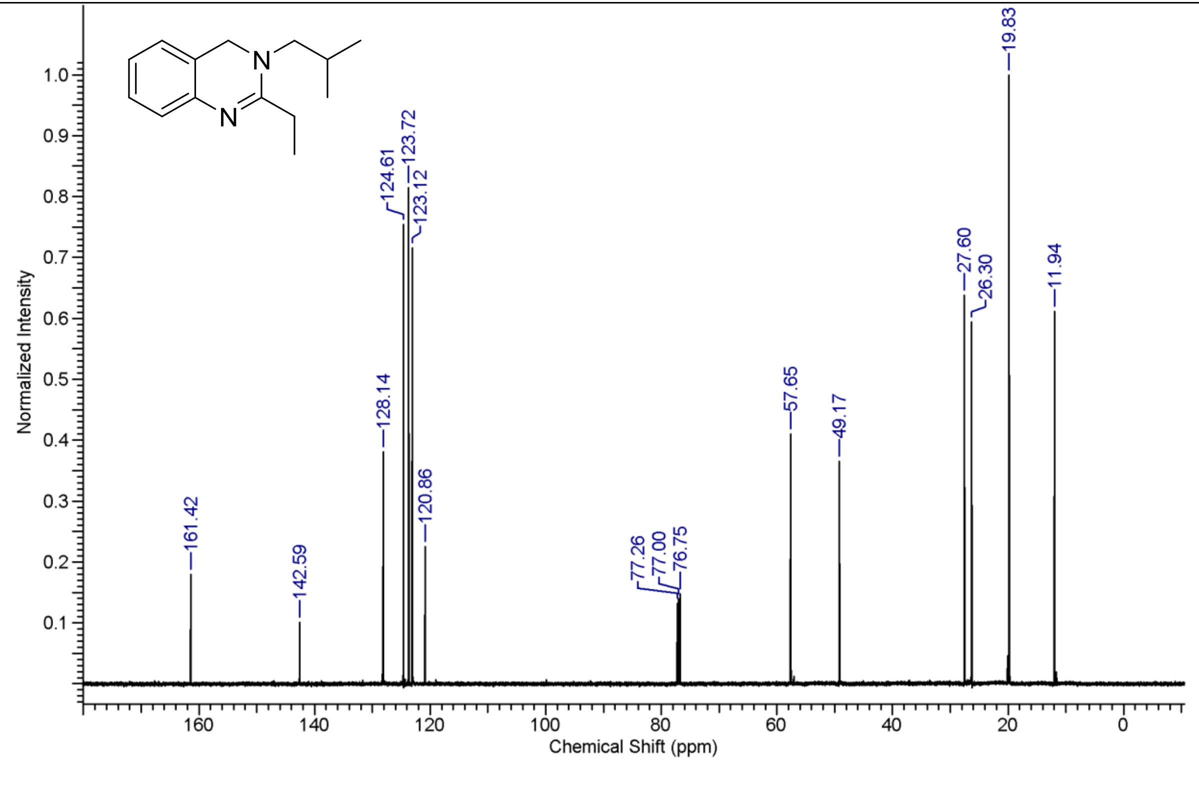

^{1}H NMR (500 MHz, $CDCl_3$) spectrum of compound **2a**

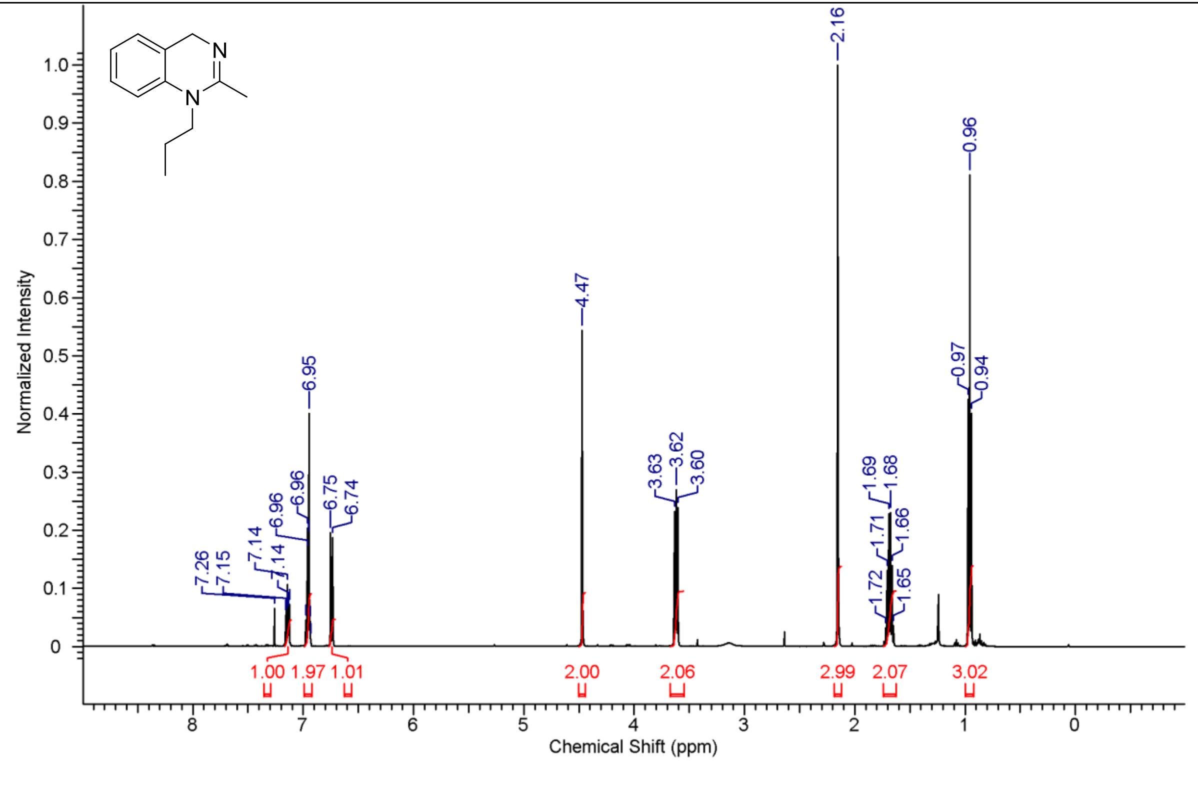

^{13}C NMR (125 MHz, $CDCl_3$) spectrum of compound **2a**

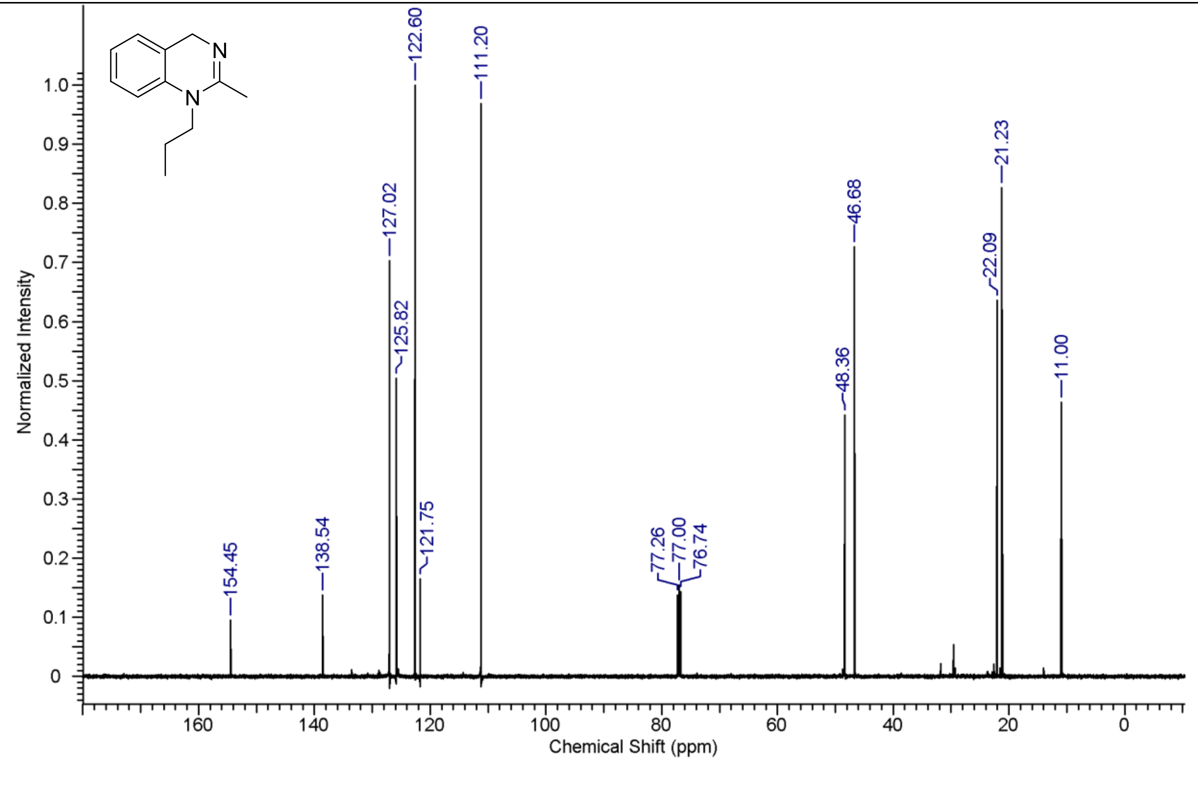

^{1}H NMR (500 MHz, $CDCl_3$) spectrum of compound **2b**

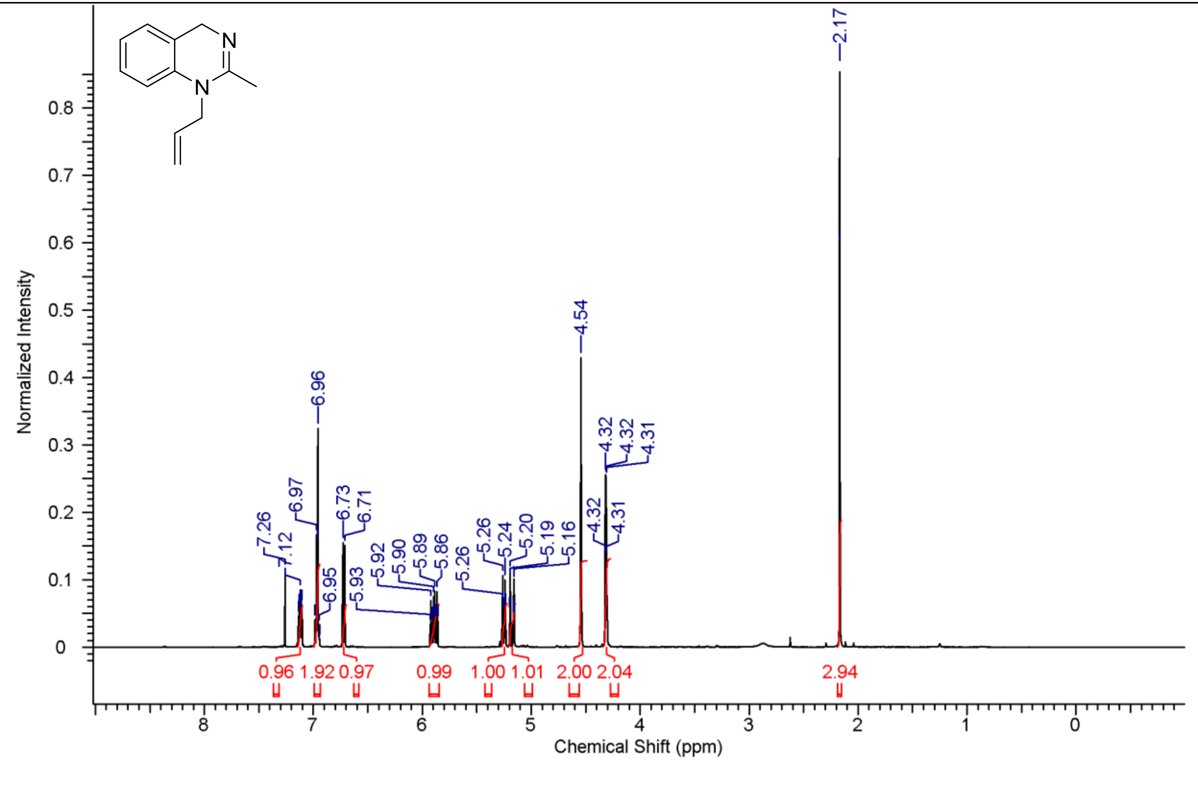

^{13}C NMR (125 MHz, $CDCl_3$) spectrum of compound **2b**

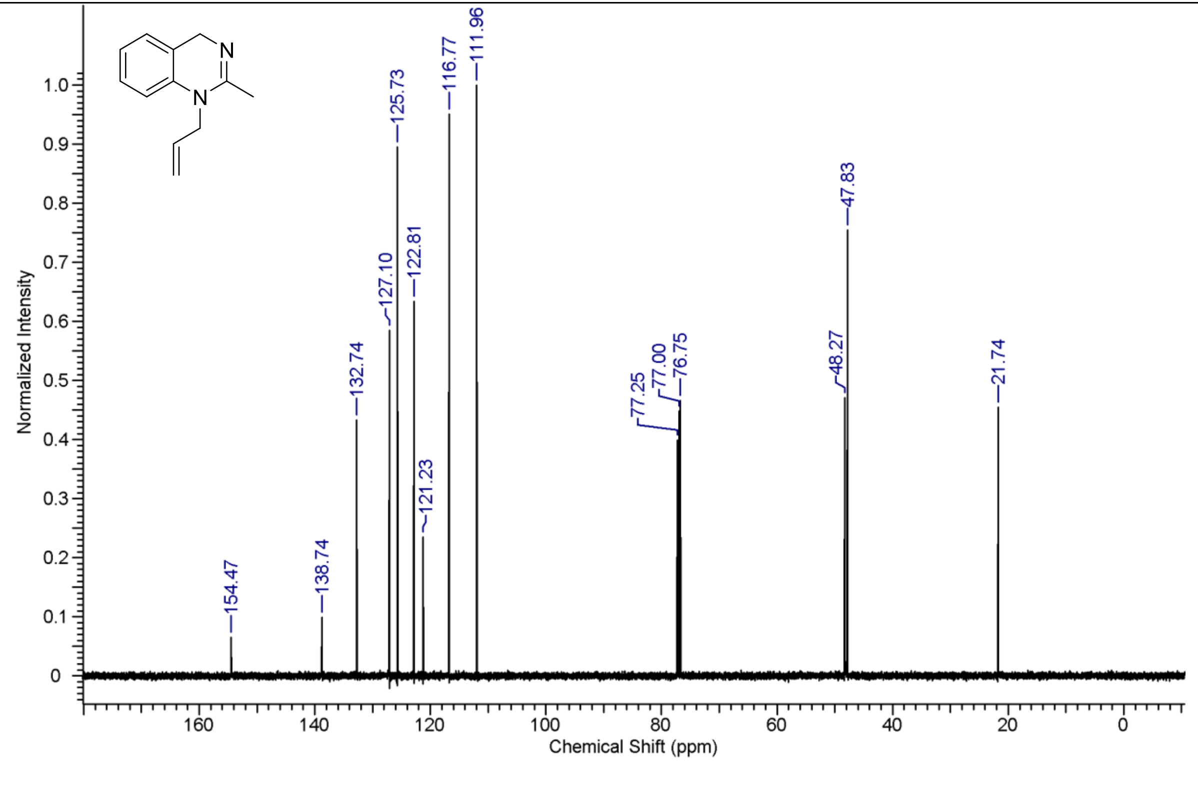

^{1}H NMR (500 MHz, $CDCl_3$) spectrum of compound **2c**

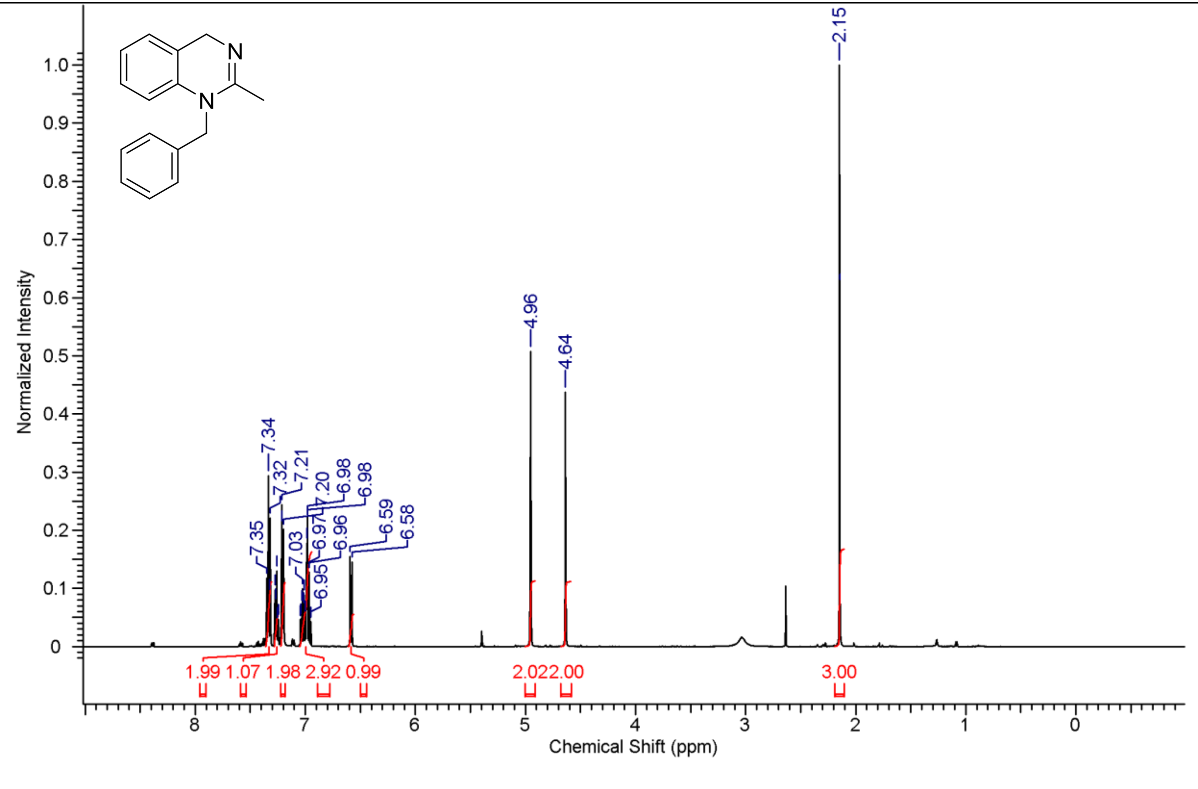

^{13}C NMR (125 MHz, $CDCl_3$) spectrum of compound **2c**

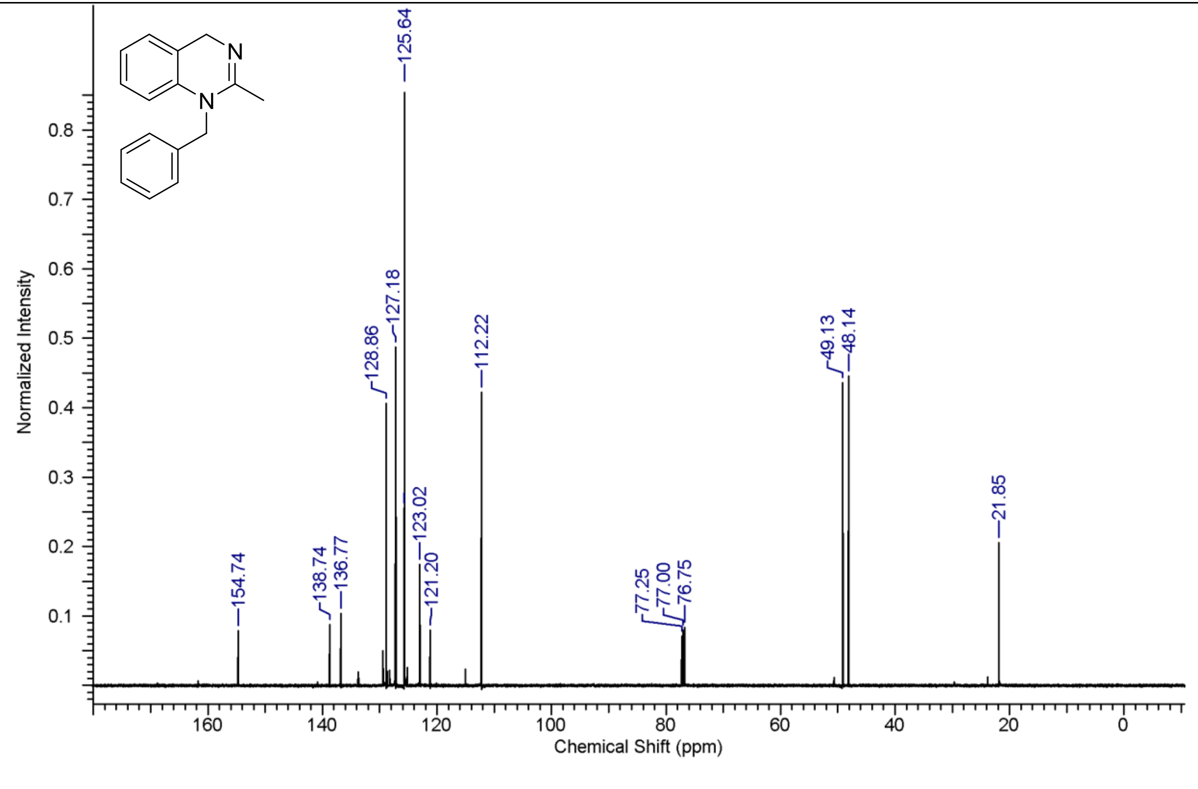

^{1}H NMR (500 MHz, $CDCl_3$) spectrum of compound **2d**

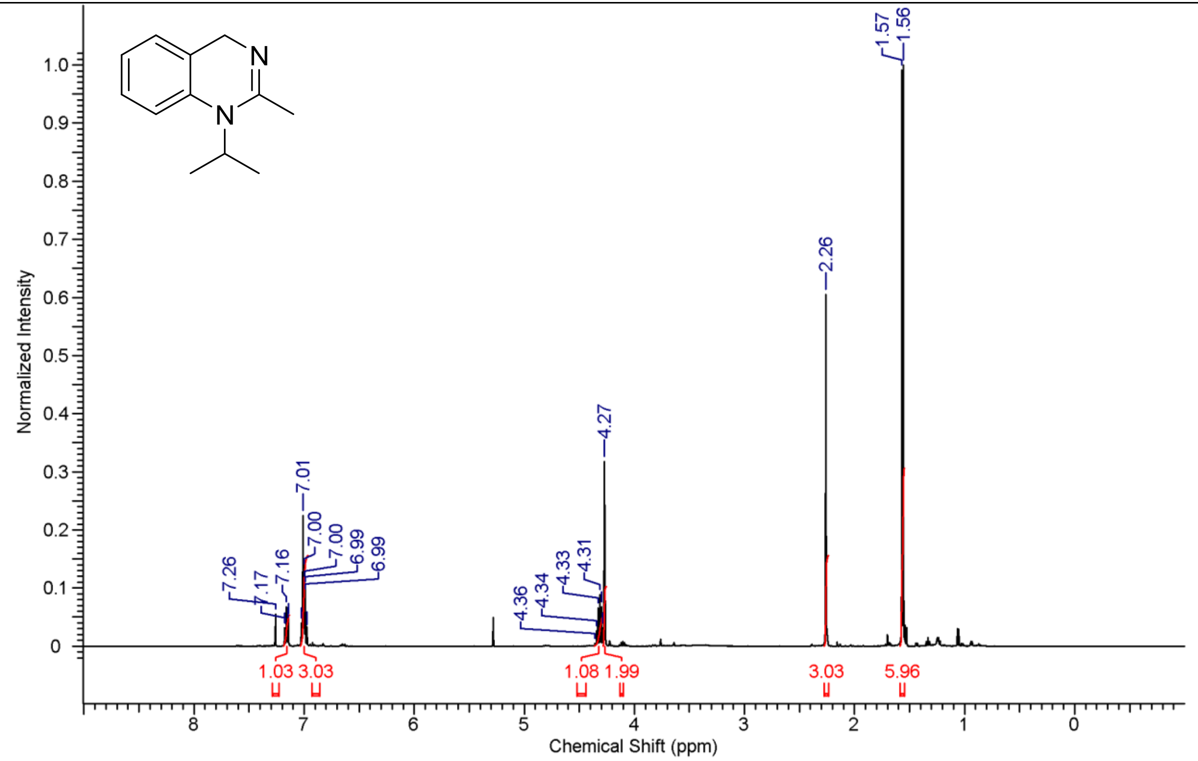

^{13}C NMR (125 MHz, $CDCl_3$) spectrum of compound **2d**

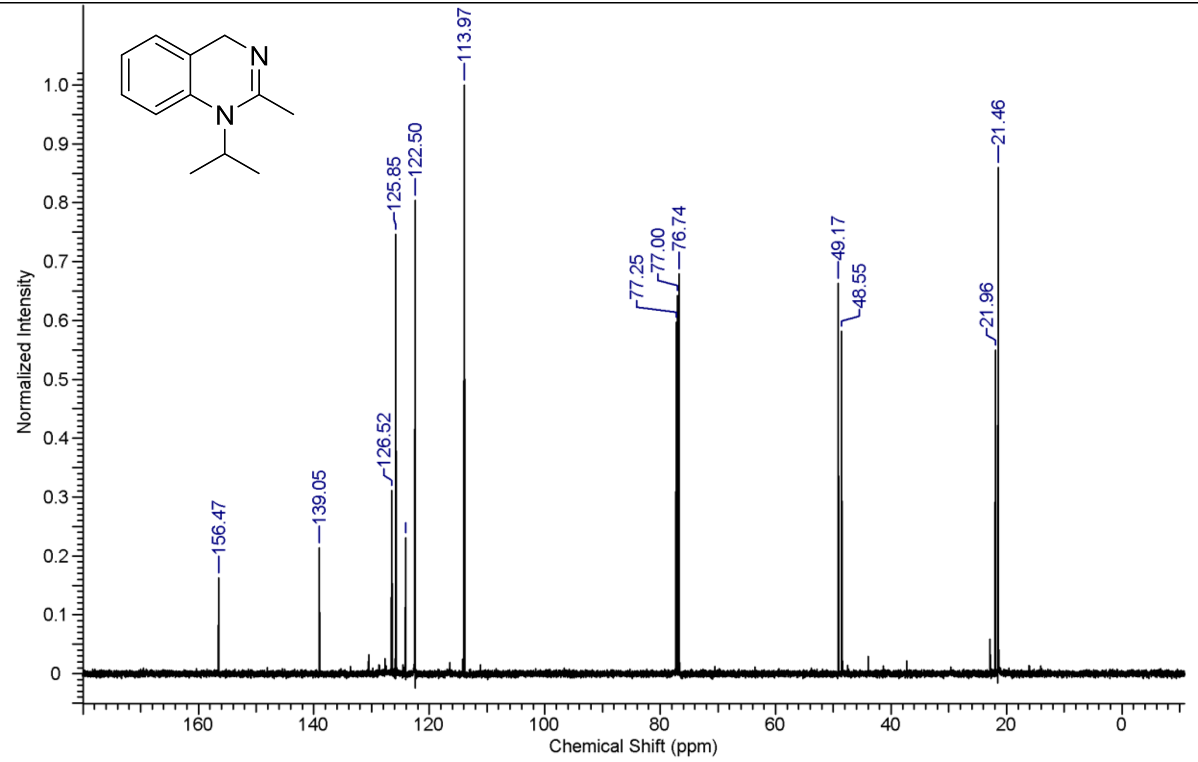

^{1}H NMR (500 MHz, $CDCl_3$) spectrum of compound **2e**

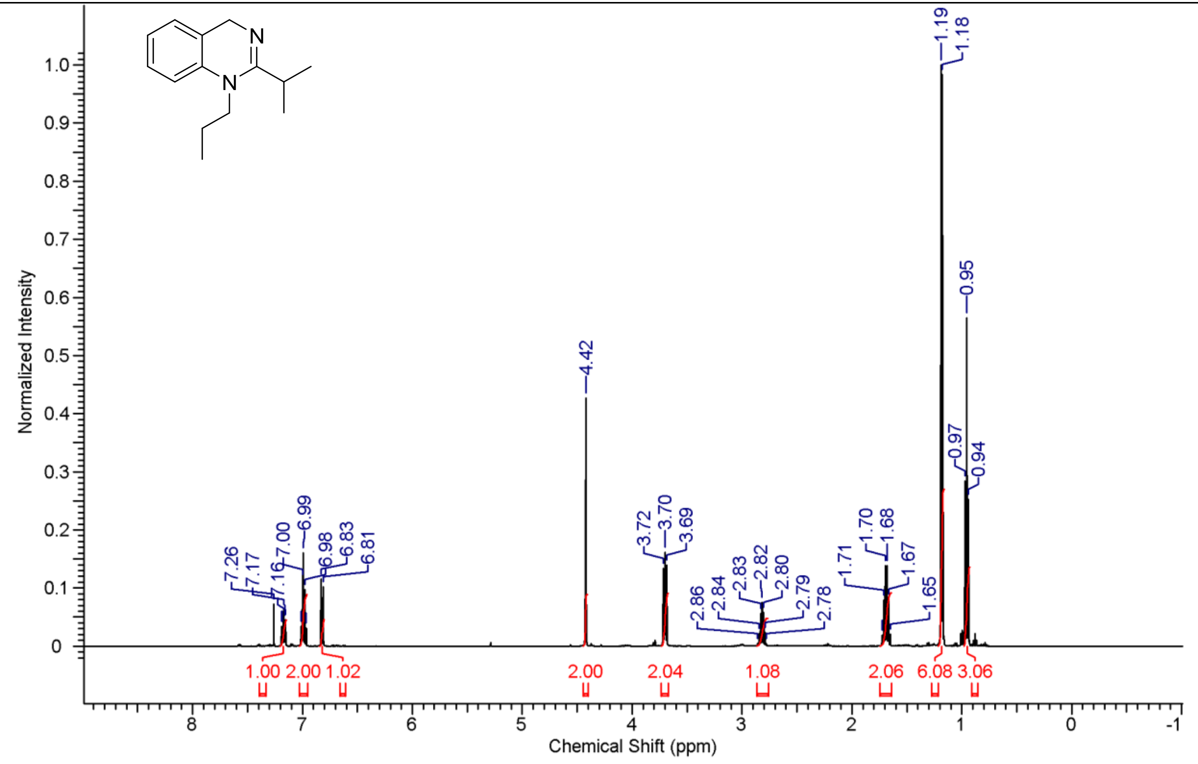

^{13}C NMR (125 MHz, $CDCl_3$) spectrum of compound **2e**

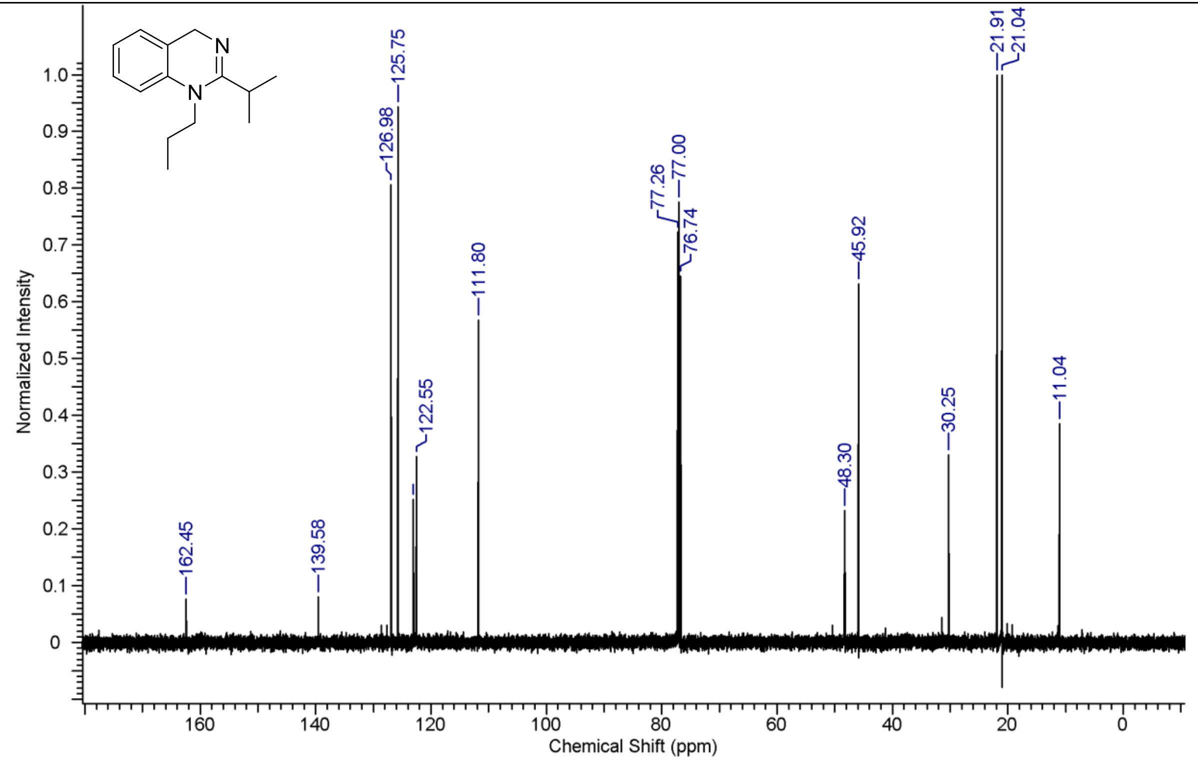

^{1}H NMR (500 MHz, $CDCl_3$) spectrum of compound **2f**

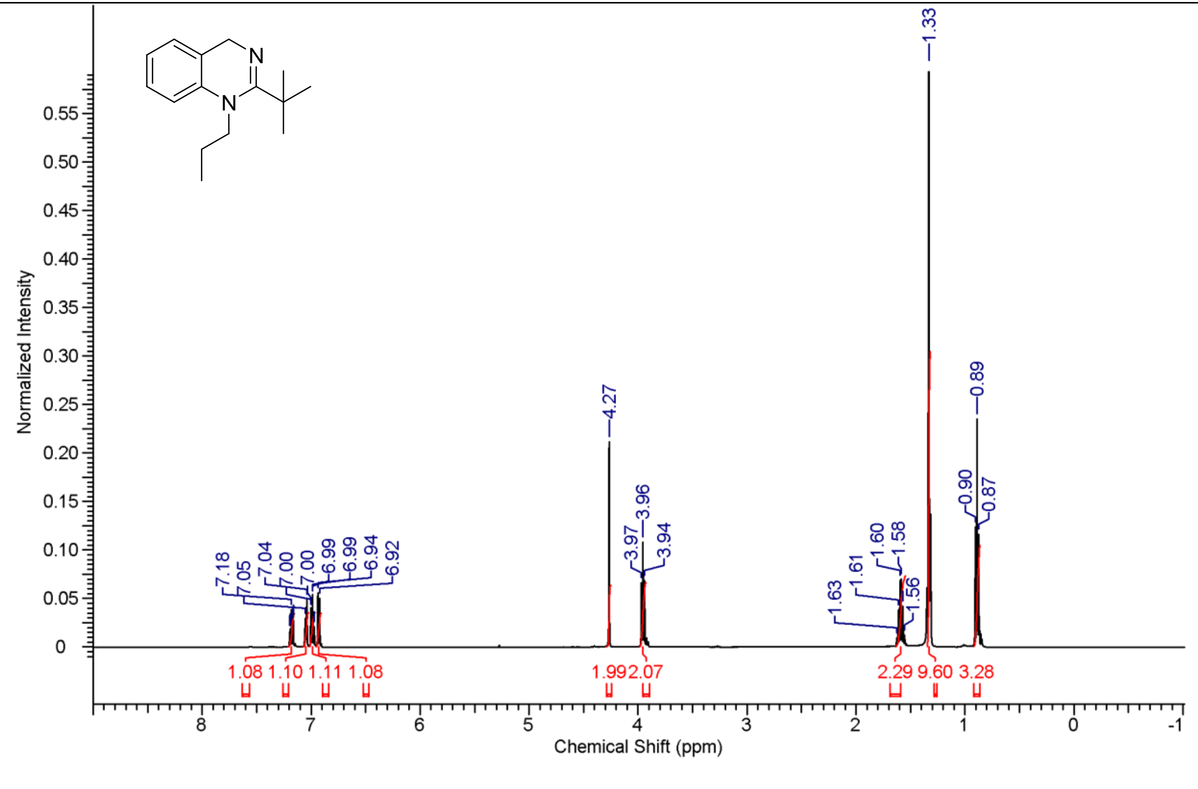

^{13}C NMR (125 MHz, $CDCl_3$) spectrum of compound **2f**

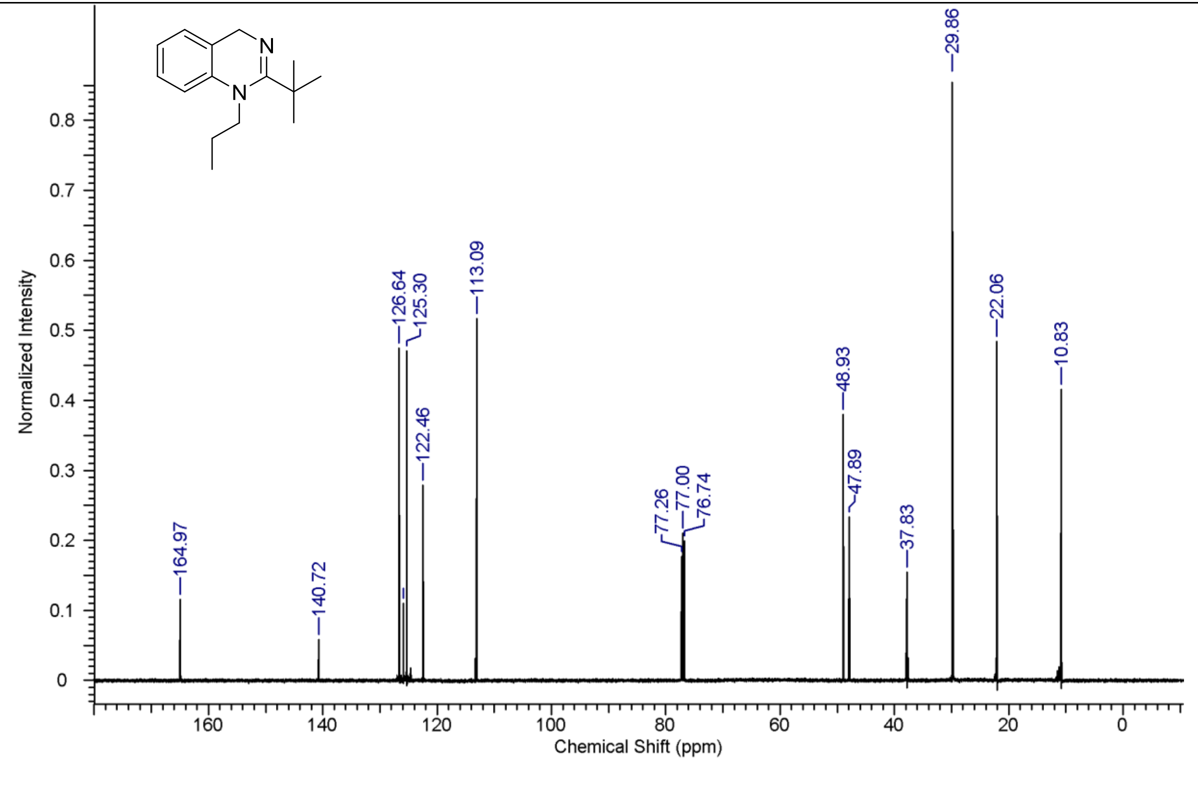

^{1}H NMR (500 MHz, $CDCl_3$) spectrum of compound **2i**

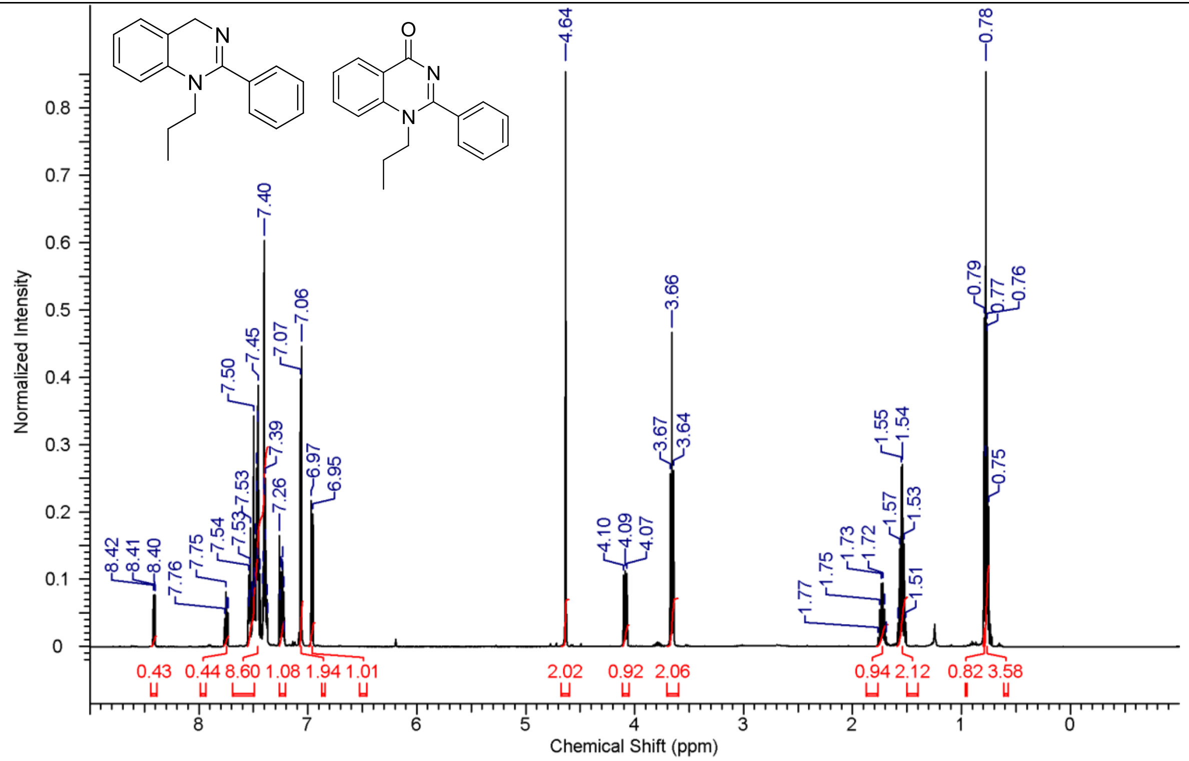

^{13}C NMR (125 MHz, $CDCl_3$) spectrum of compound **2i**

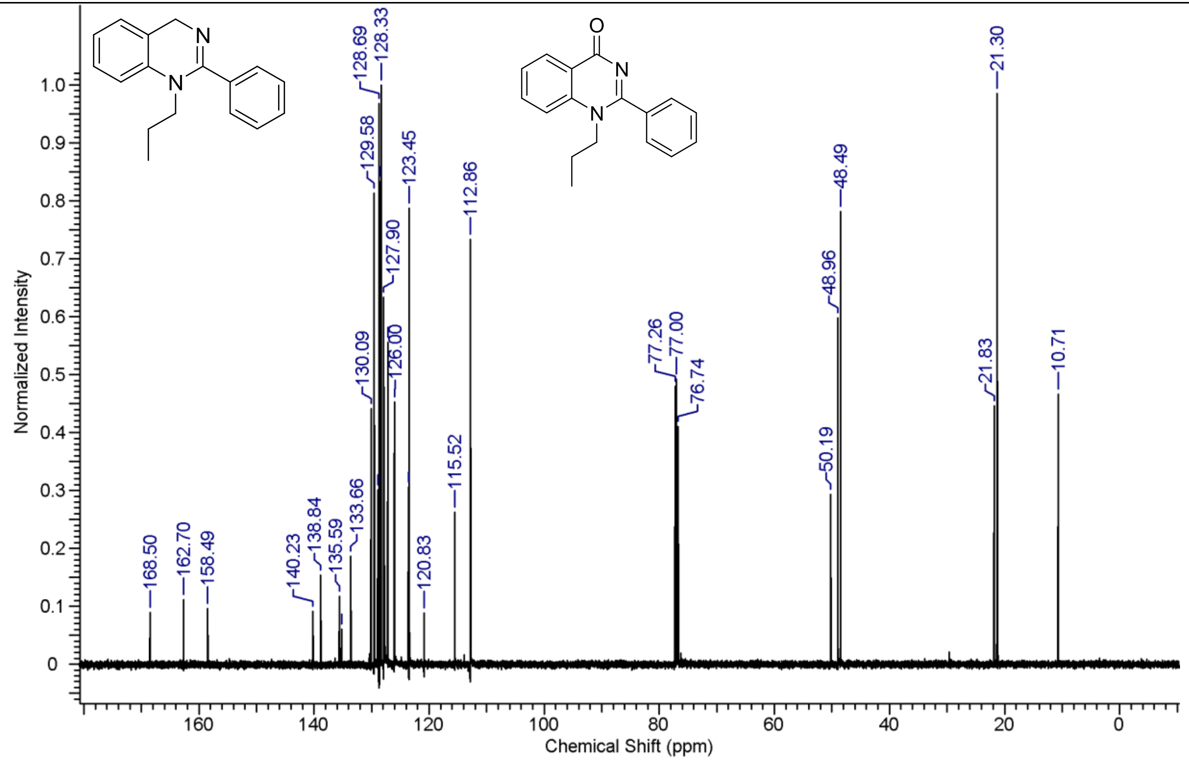

^{1}H NMR (500 MHz, $CDCl_3$) spectrum of compound **2j**

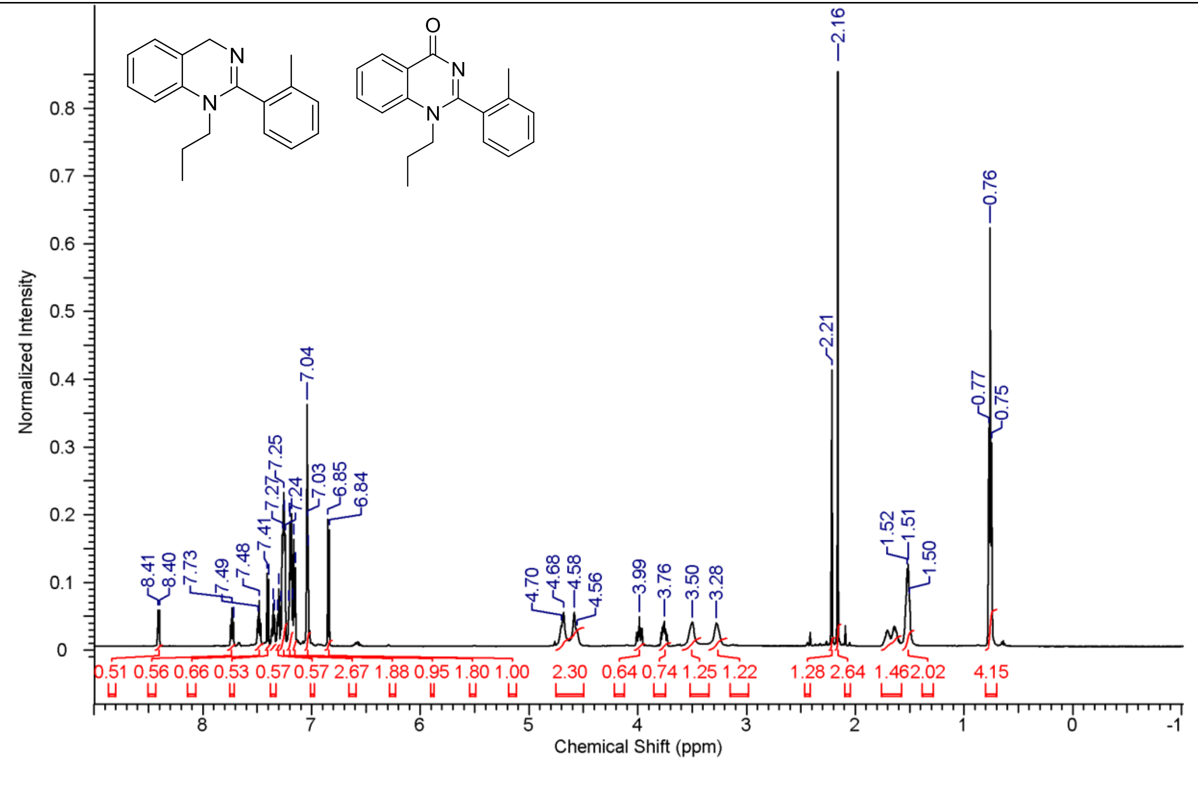

^{13}C NMR (125 MHz, $CDCl_3$) spectrum of compound **2j**

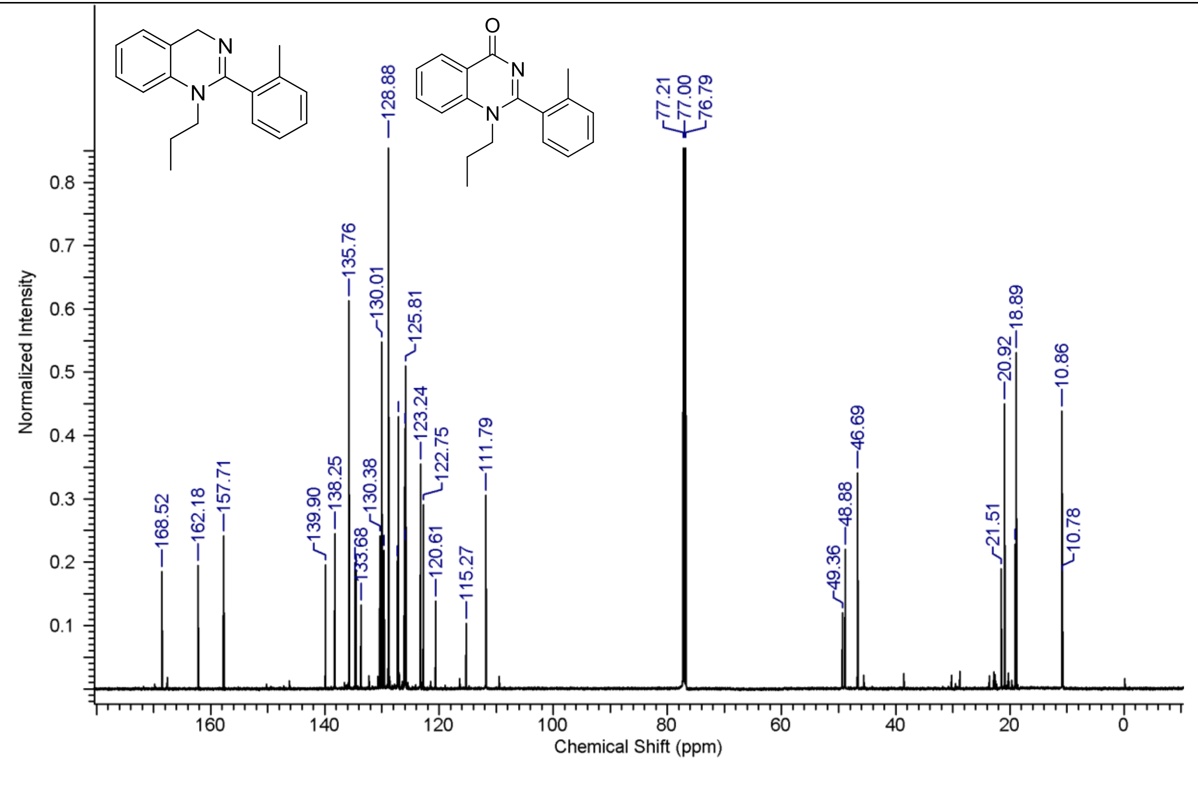

^{1}H NMR (500 MHz, $CDCl_3$) spectrum of compound **6a**

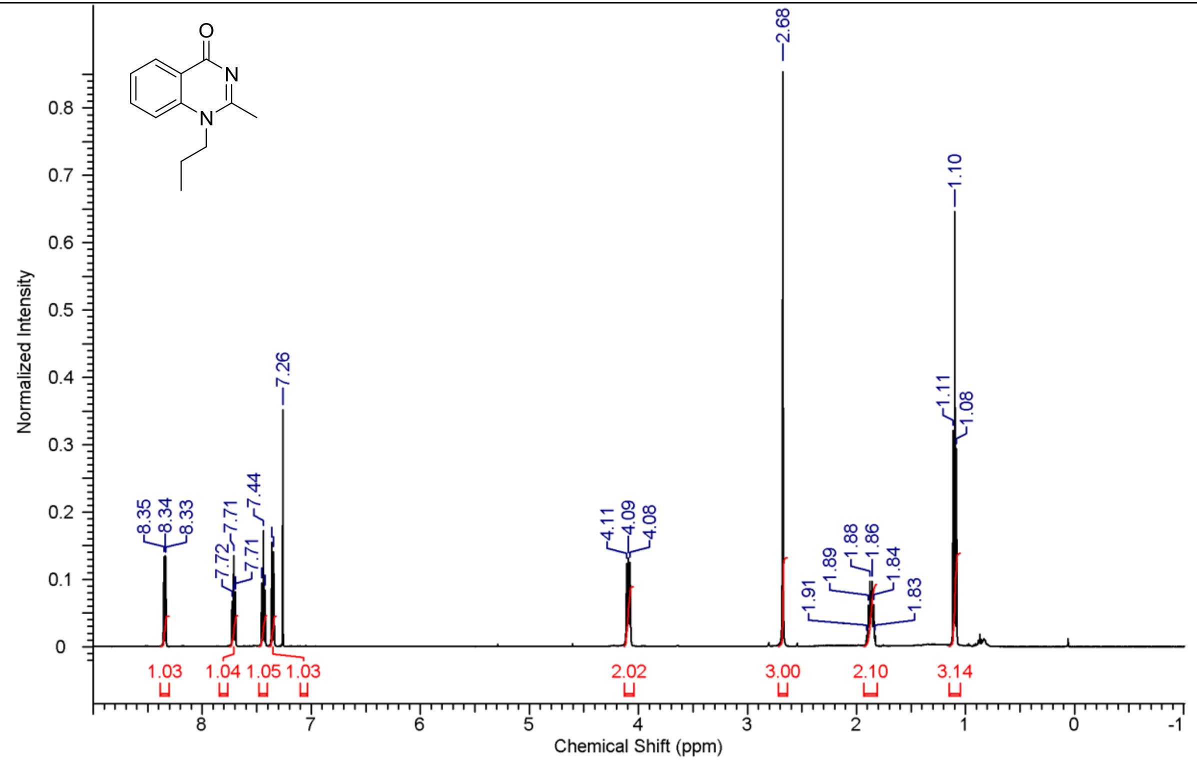

^{13}C NMR (125 MHz, $CDCl_3$) spectrum of compound **6a**

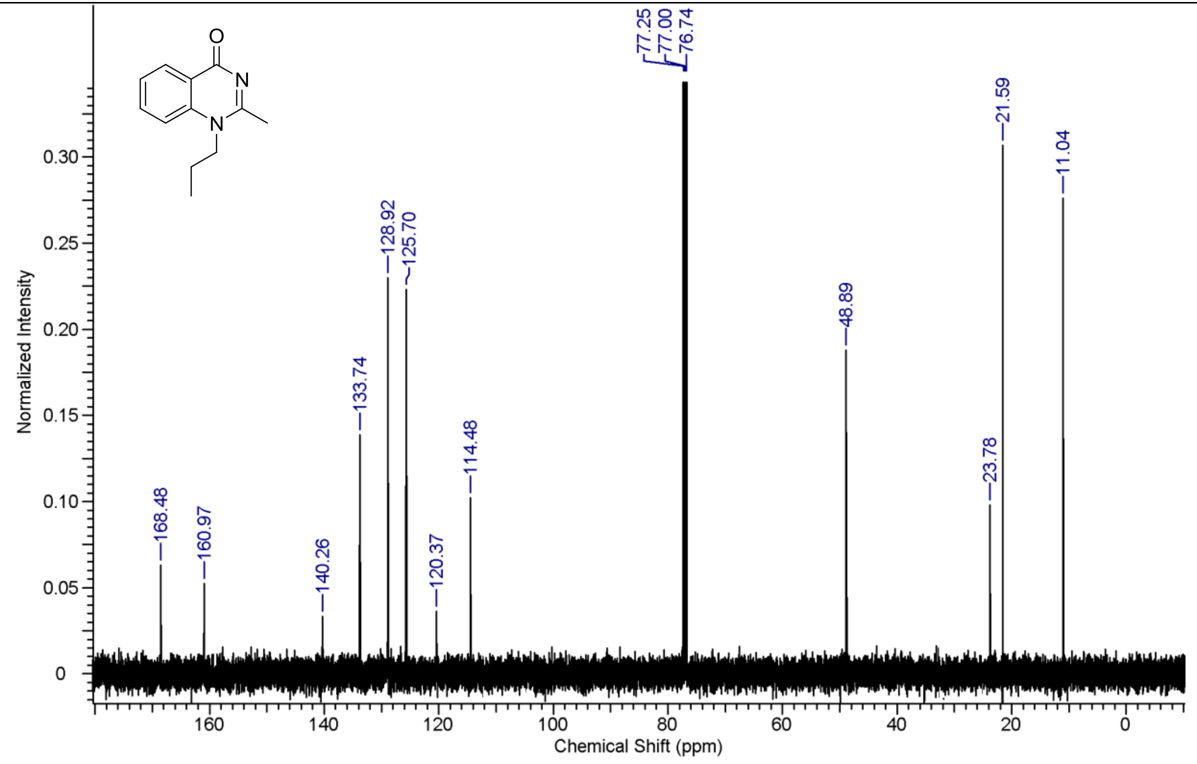

^{1}H NMR (500 MHz, $CDCl_3$) spectrum of compound **3a**

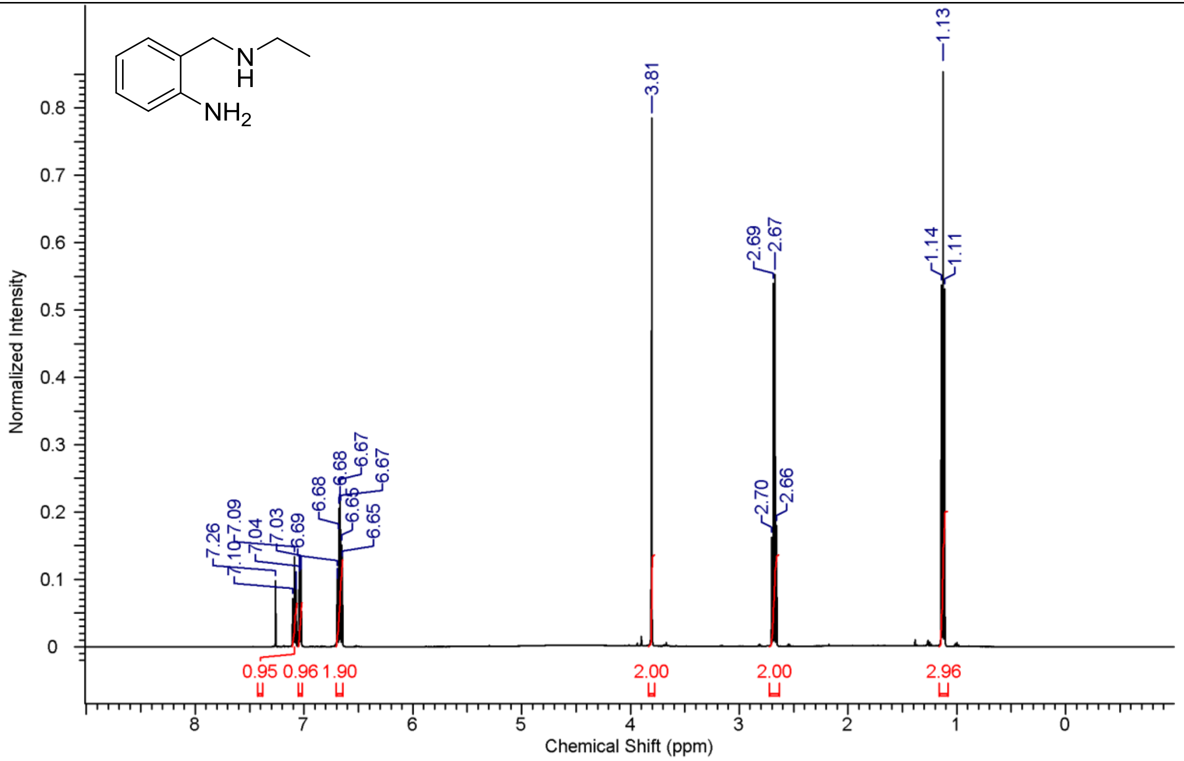

^{13}C NMR (125 MHz, $CDCl_3$) spectrum of compound **3a**

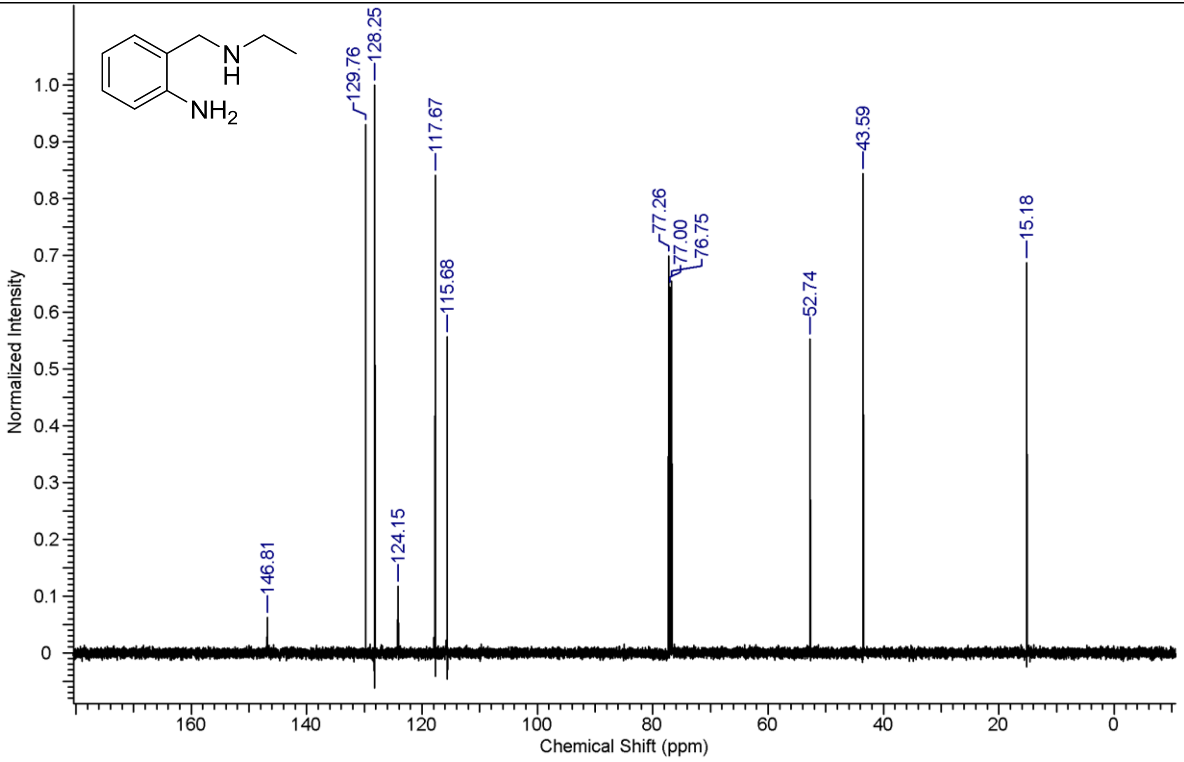

^{1}H NMR (500 MHz, $CDCl_3$) spectrum of compound **3b**

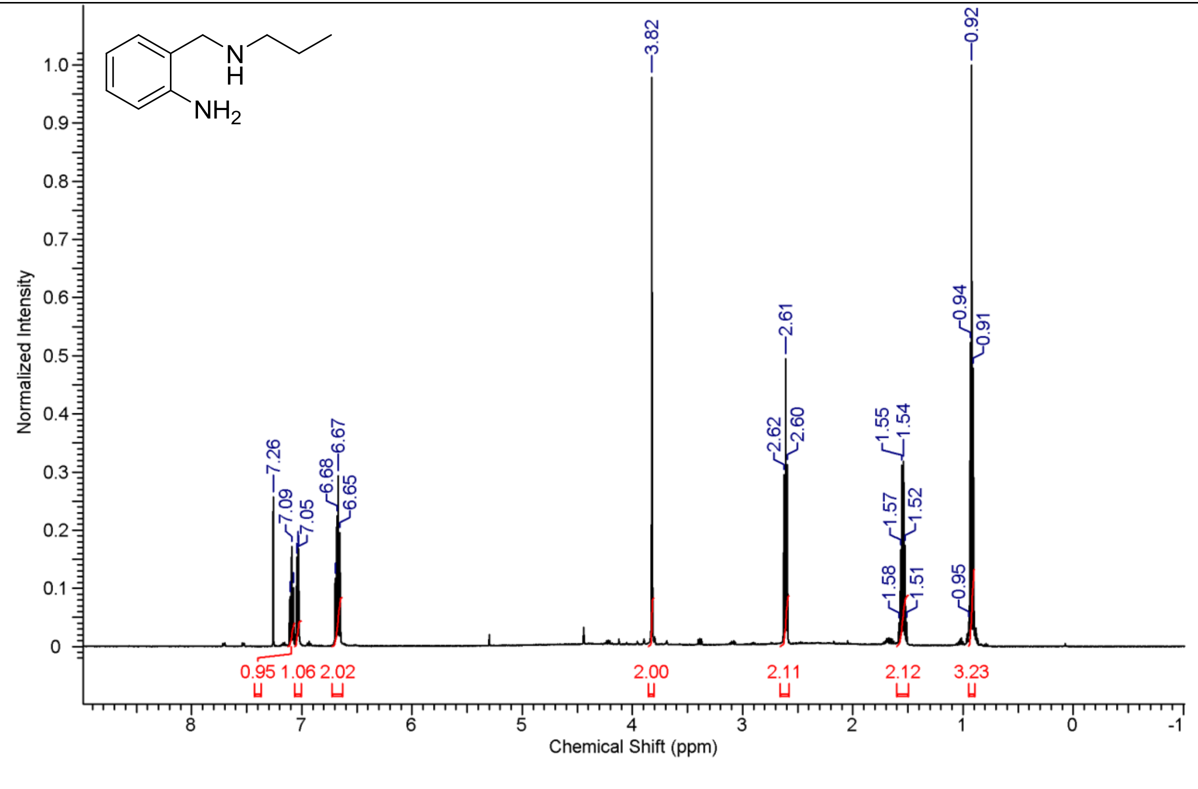

^{13}C NMR (125 MHz, $CDCl_3$) spectrum of compound **3b**

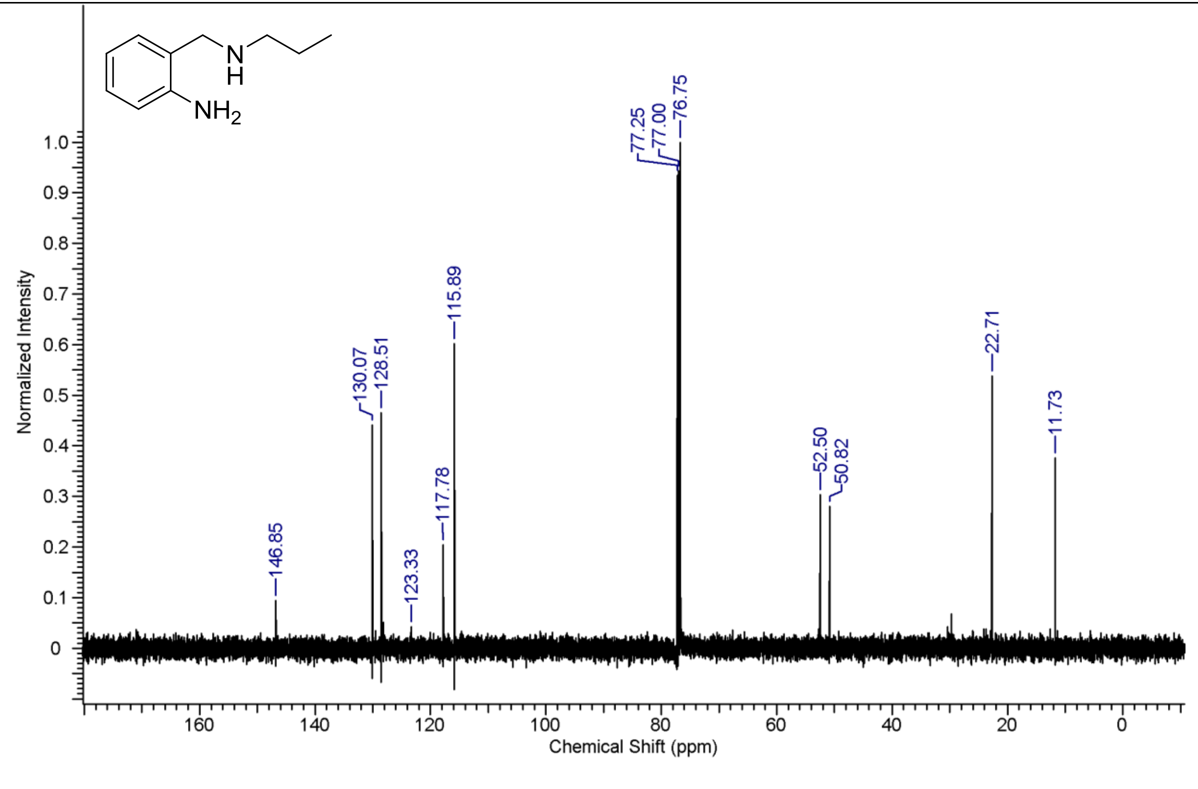

^{1}H NMR (500 MHz, $CDCl_3$) spectrum of compound **3c**

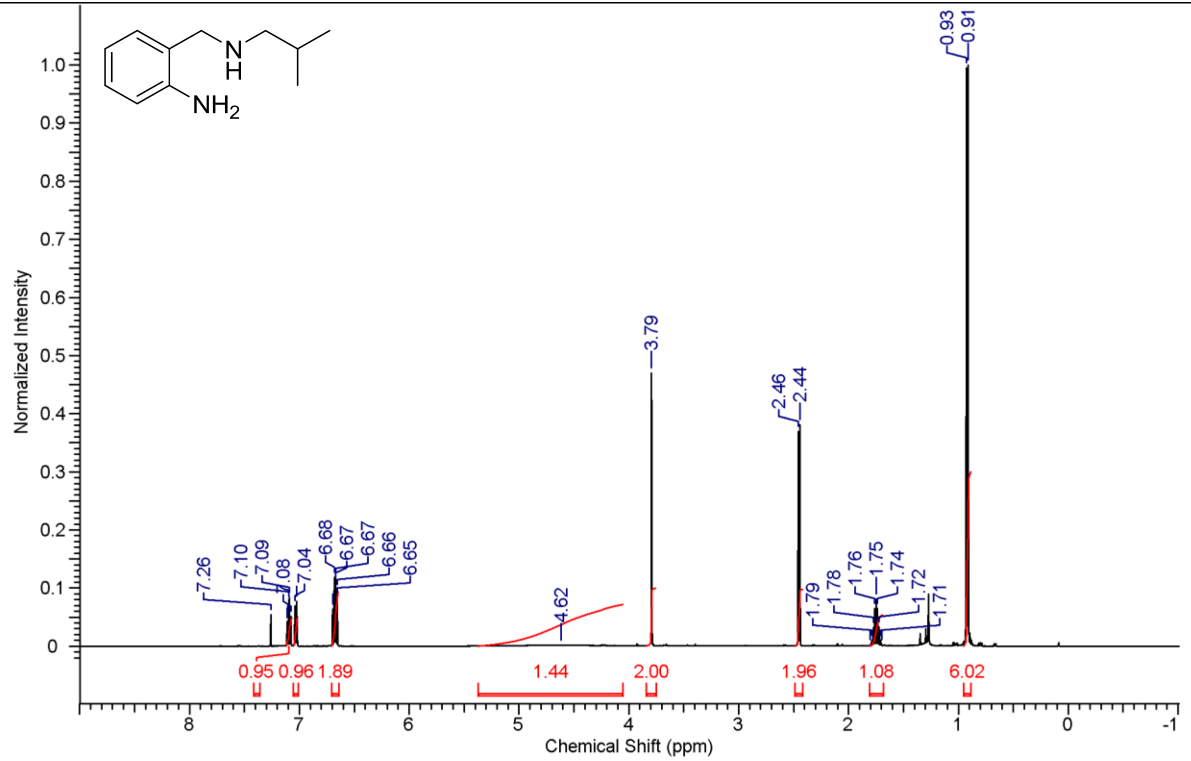

^{13}C NMR (125 MHz, $CDCl_3$) spectrum of compound **3c**

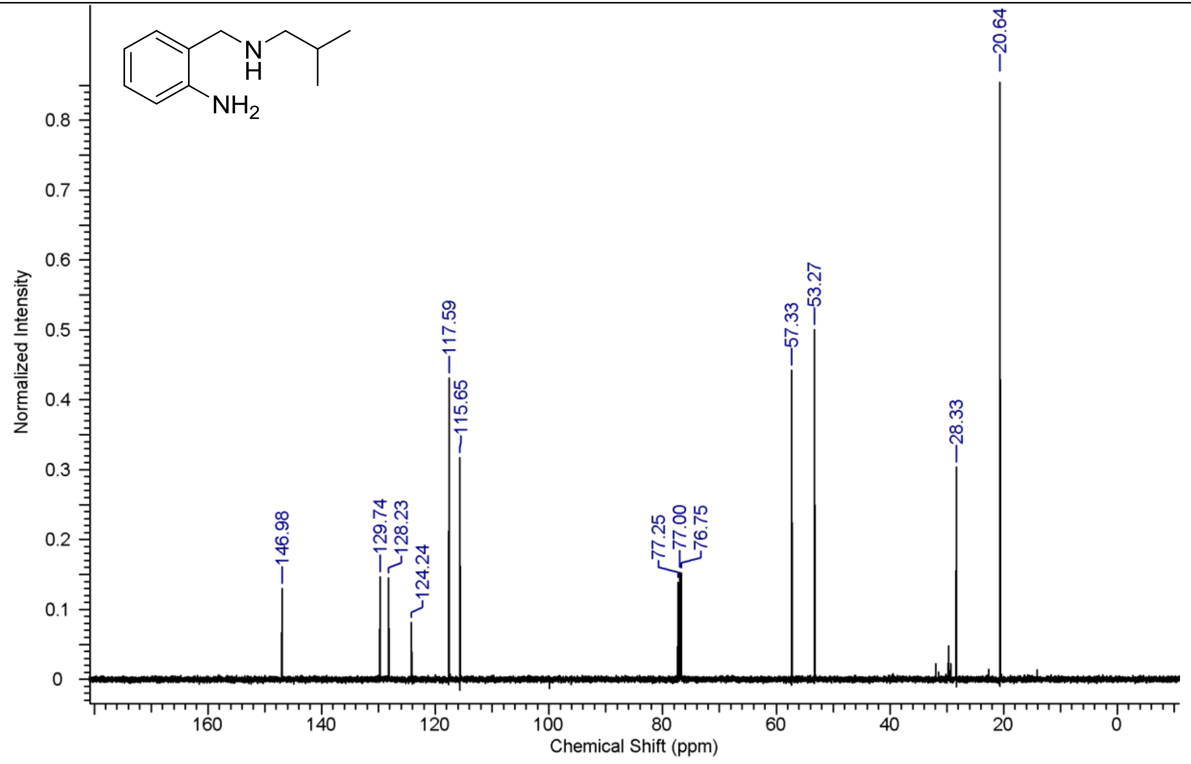

^{1}H NMR (500 MHz, $CDCl_3$) spectrum of compound **4a**

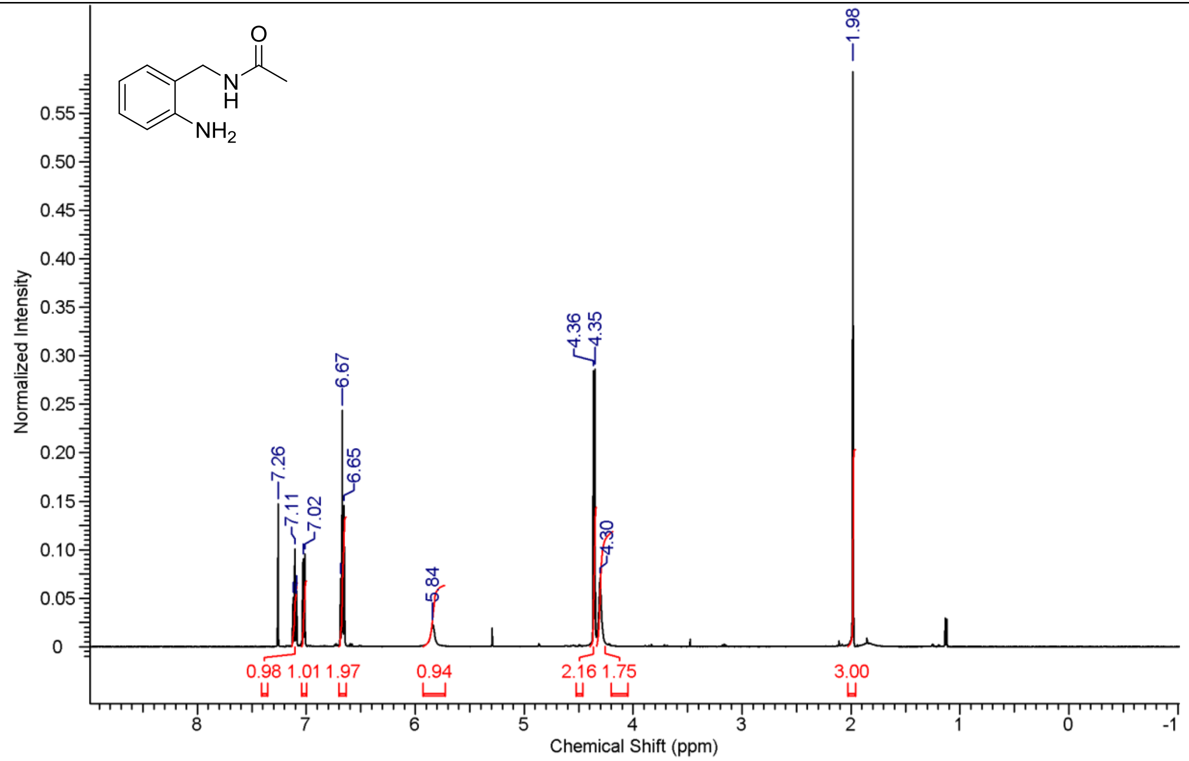

^{13}C NMR (125 MHz, $CDCl_3$) spectrum of compound **4a**

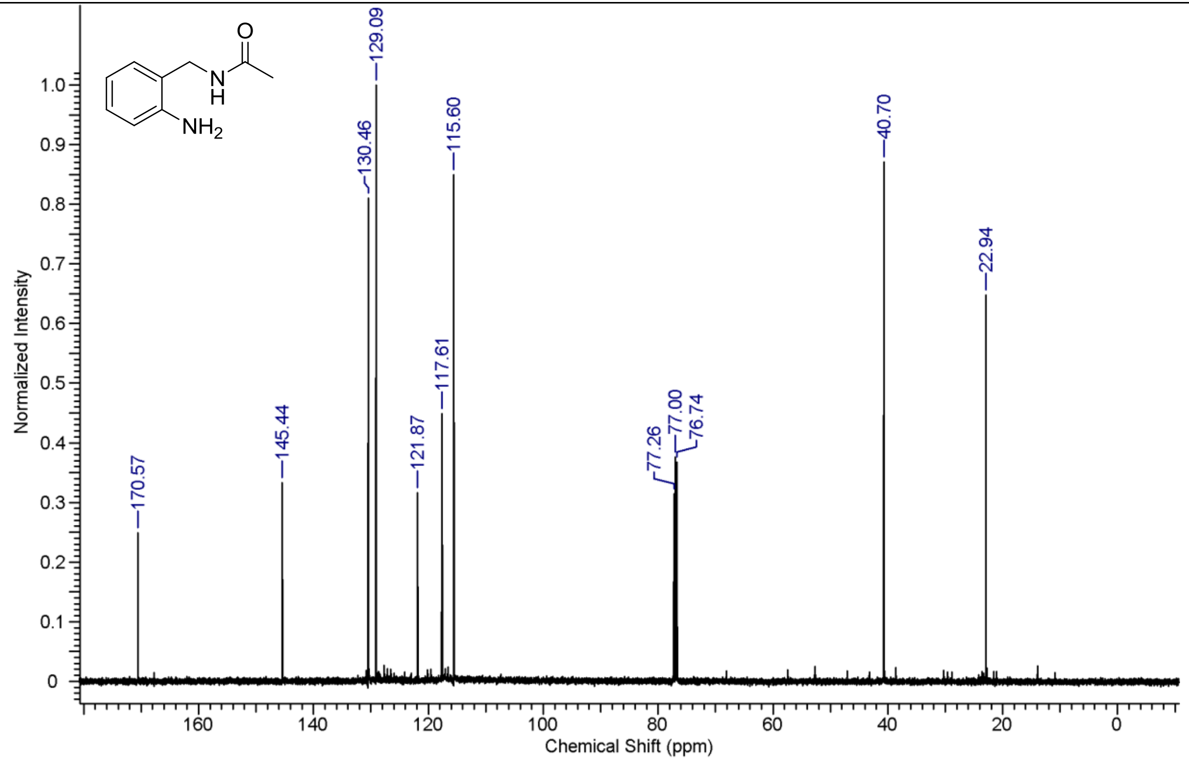

^{1}H NMR (500 MHz, $CDCl_3$) spectrum of compound **4b**

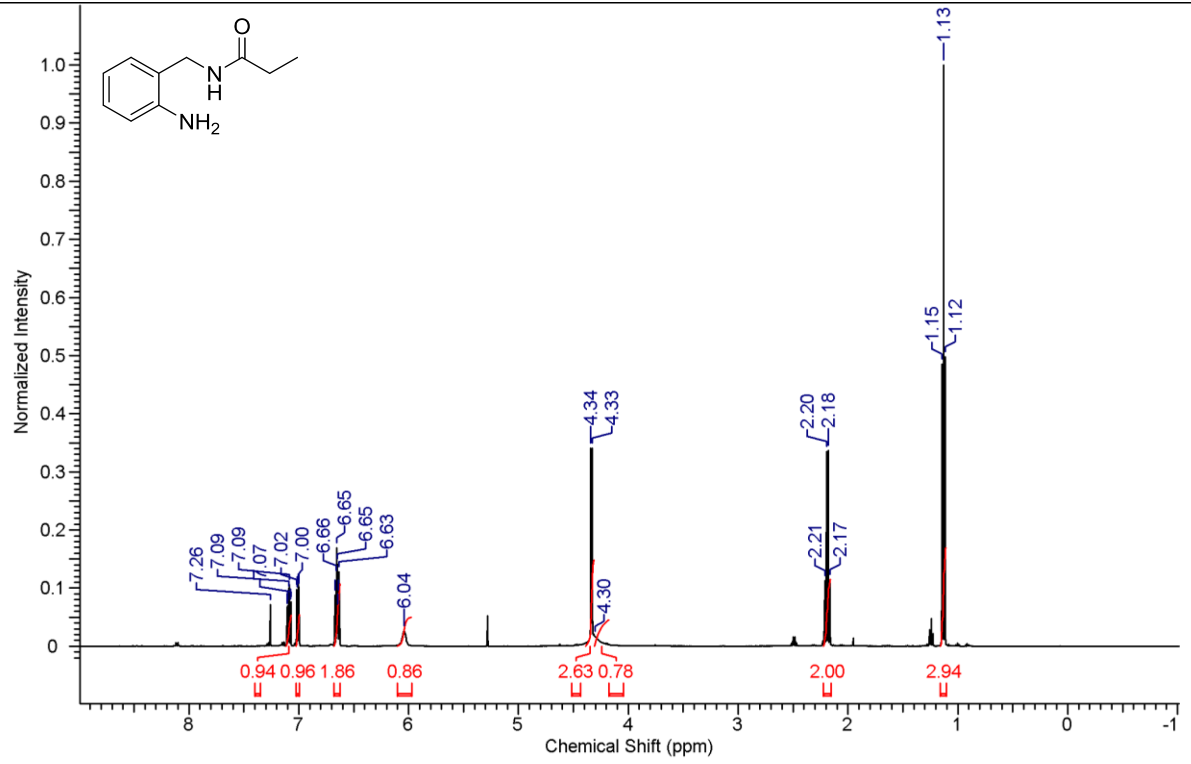

^{13}C NMR (125 MHz, $CDCl_3$) spectrum of compound **4b**

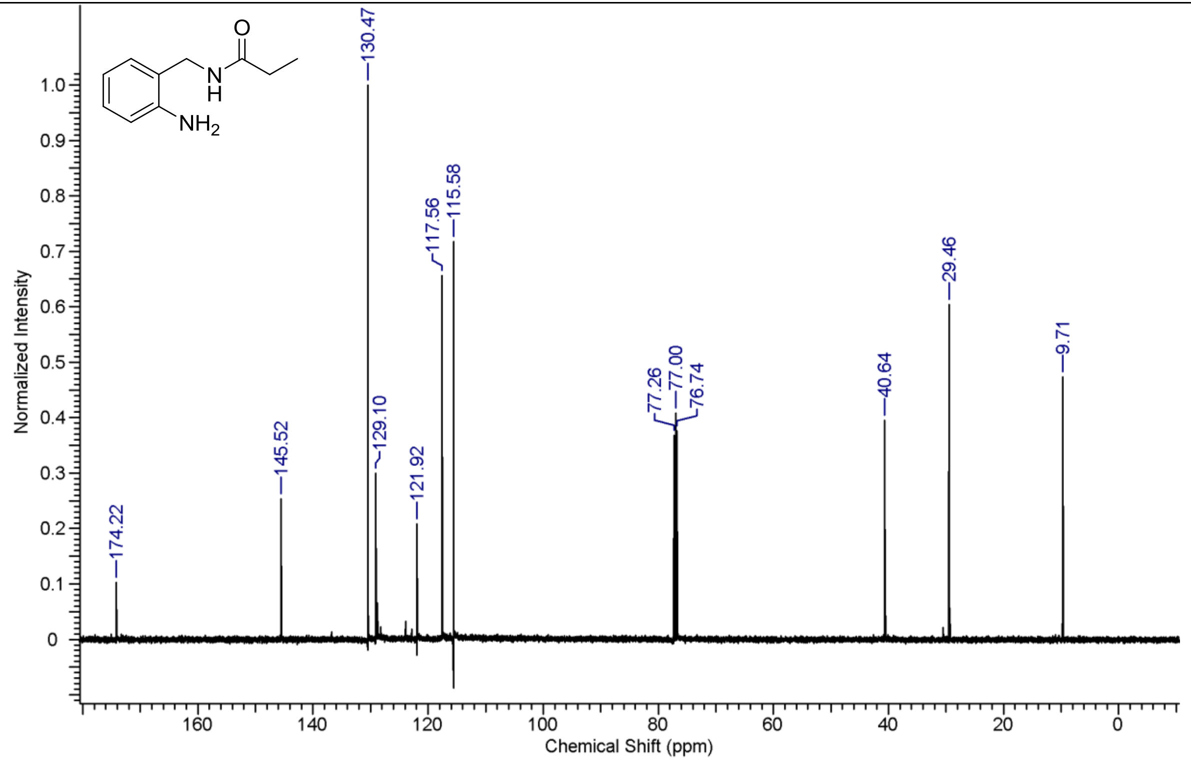

^{1}H NMR (500 MHz, $CDCl_3$) spectrum of compound **4c**

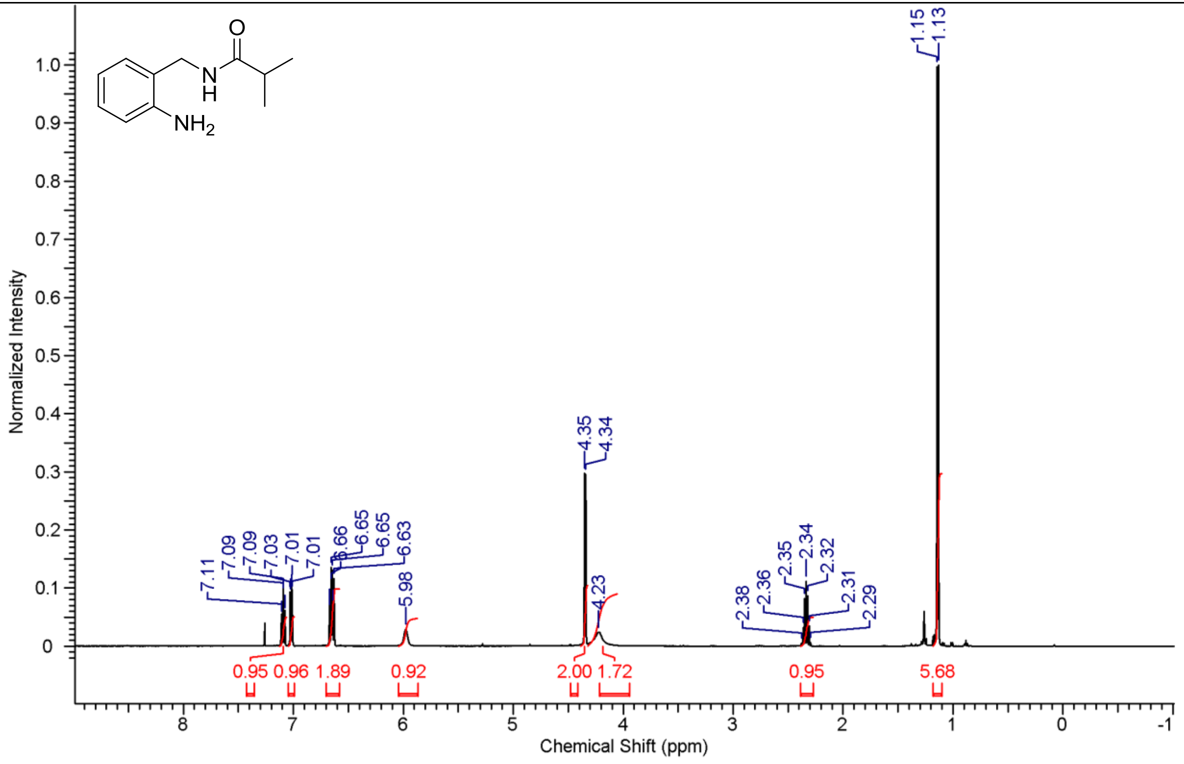

^{13}C NMR (125 MHz, $CDCl_3$) spectrum of compound **4c**

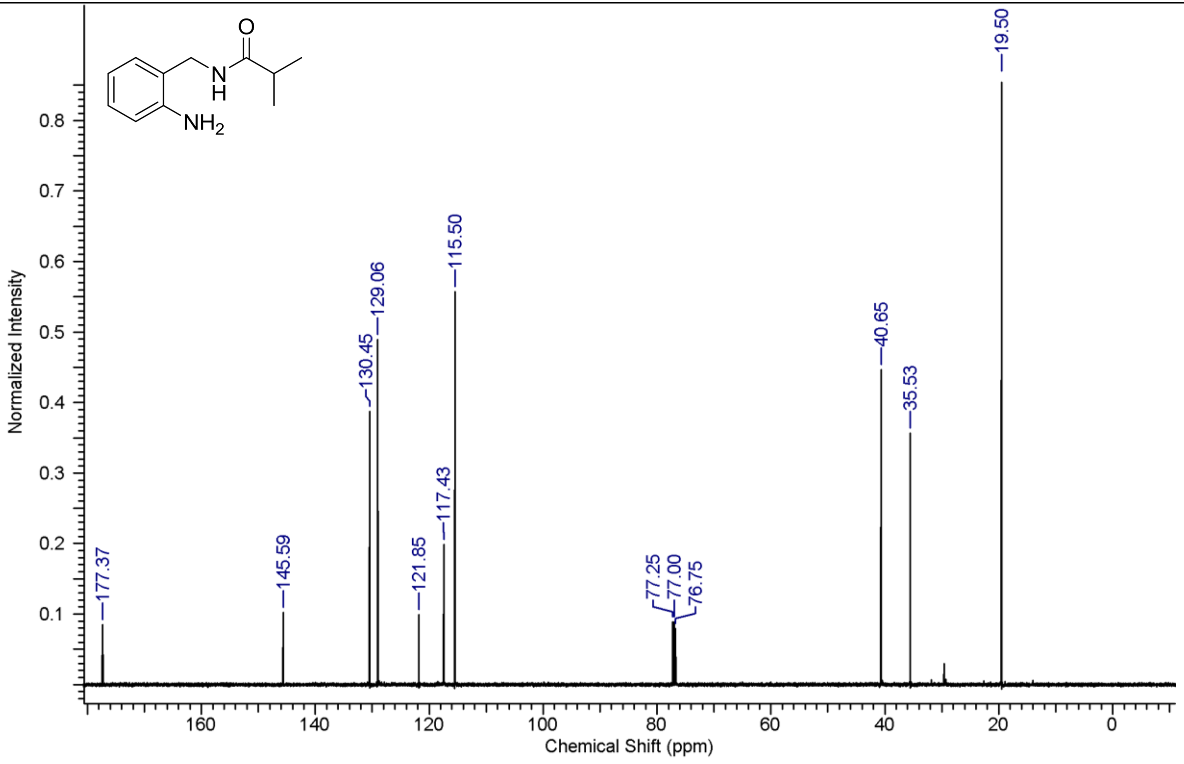

^{1}H NMR (500 MHz, $CDCl_3$) spectrum of compound **4d**

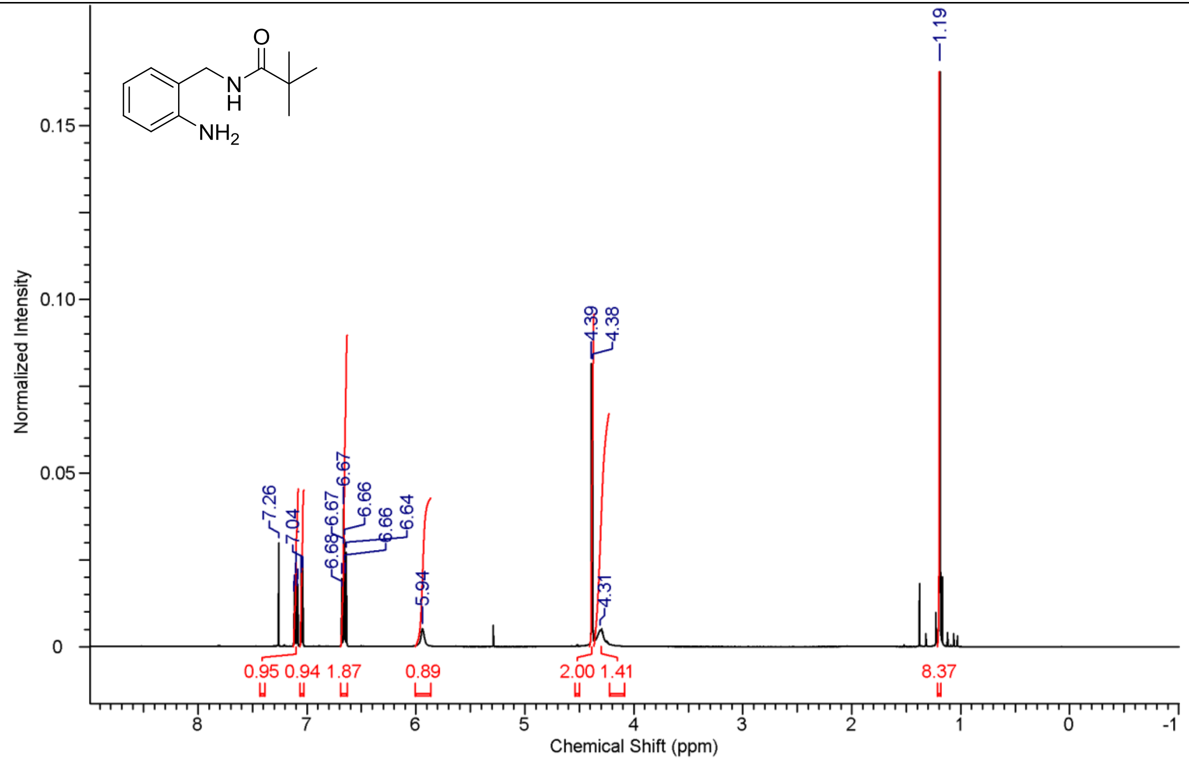

^{13}C NMR (125 MHz, $CDCl_3$) spectrum of compound **4d**

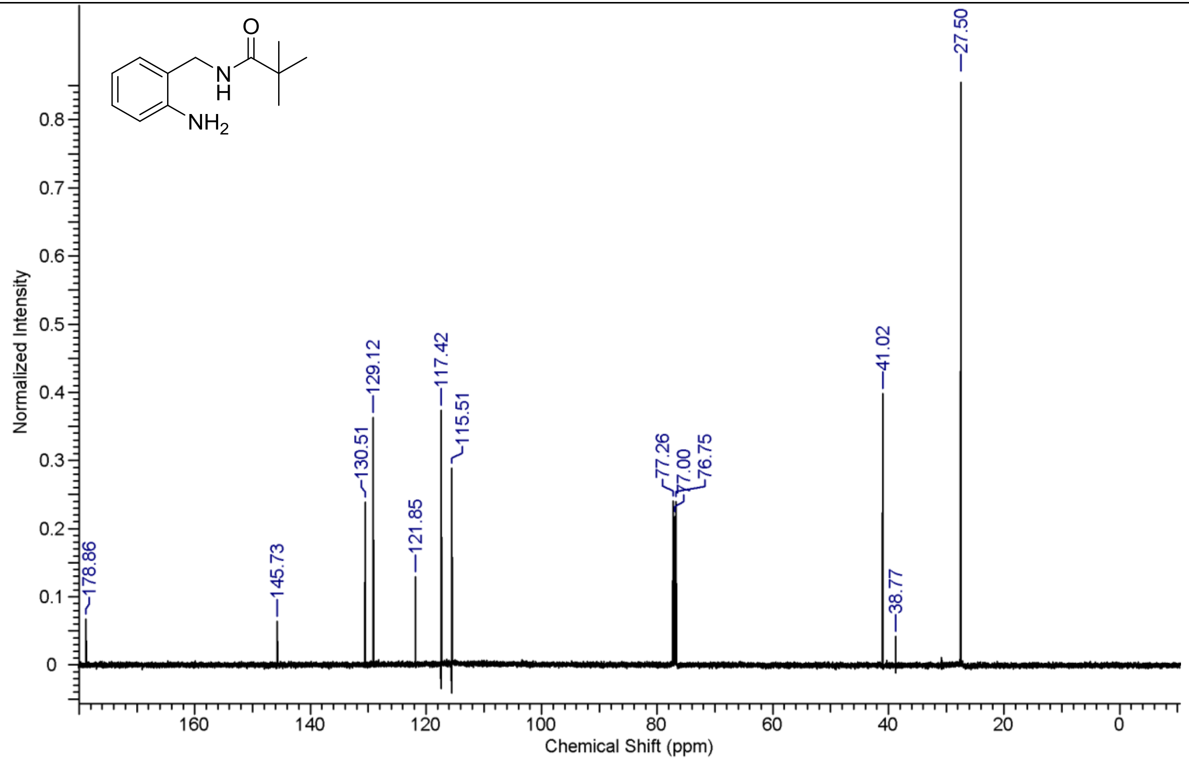

^{1}H NMR (500 MHz, $CDCl_3$) spectrum of compound **4e**

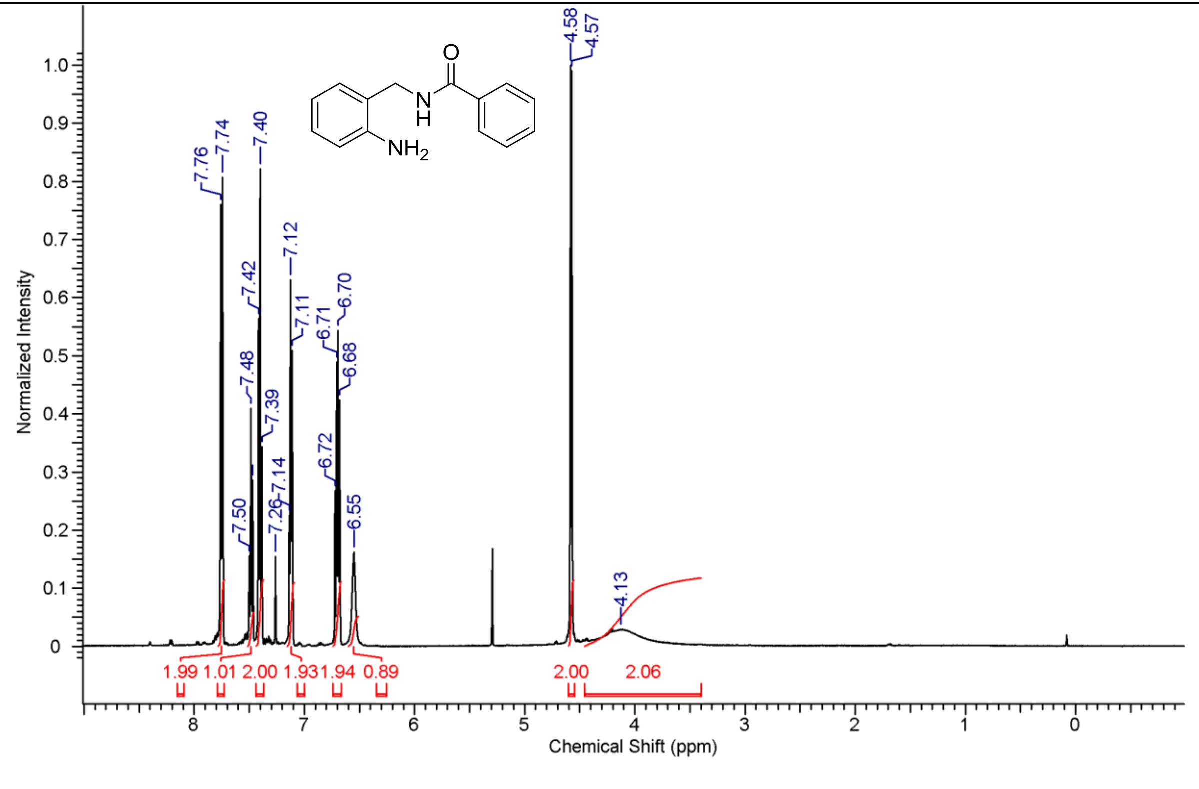

^{13}C NMR (125 MHz, $CDCl_3$) spectrum of compound **4e**

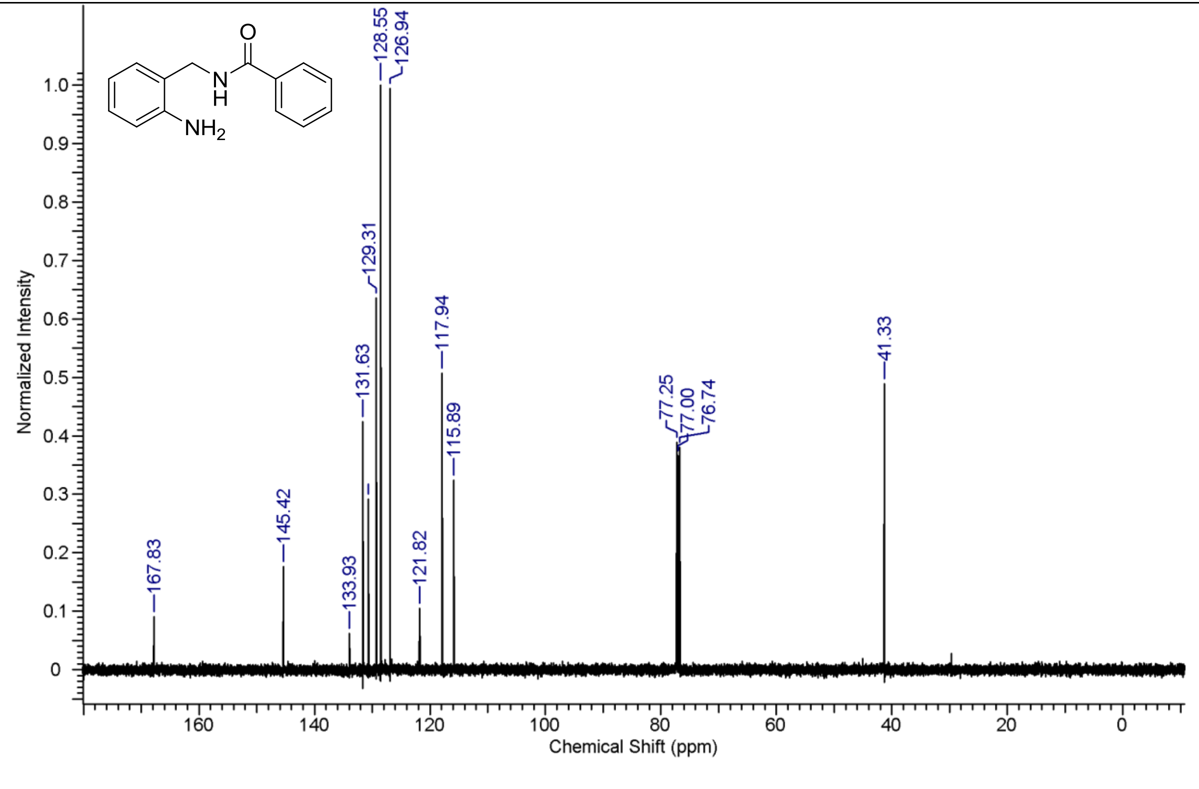

^{1}H NMR (500 MHz, $CDCl_3$) spectrum of compound **4f**

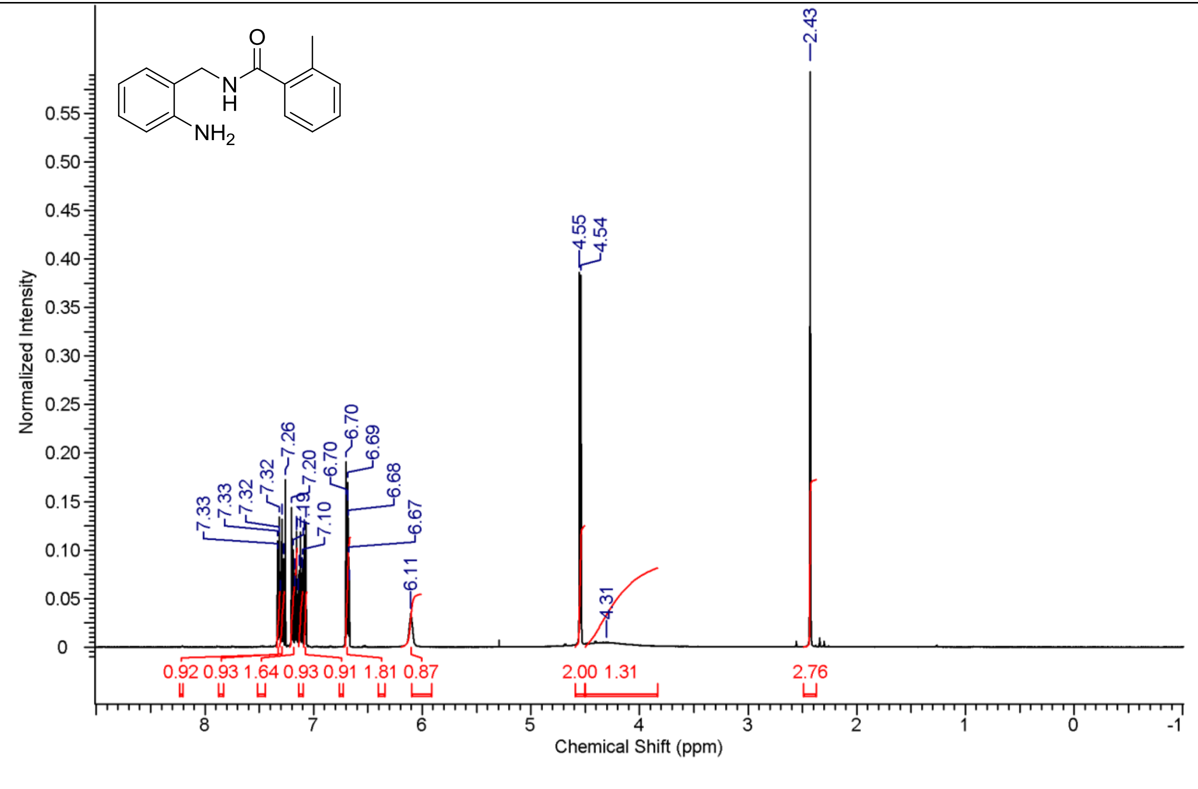

^{13}C NMR (125 MHz, $CDCl_3$) spectrum of compound **4f**

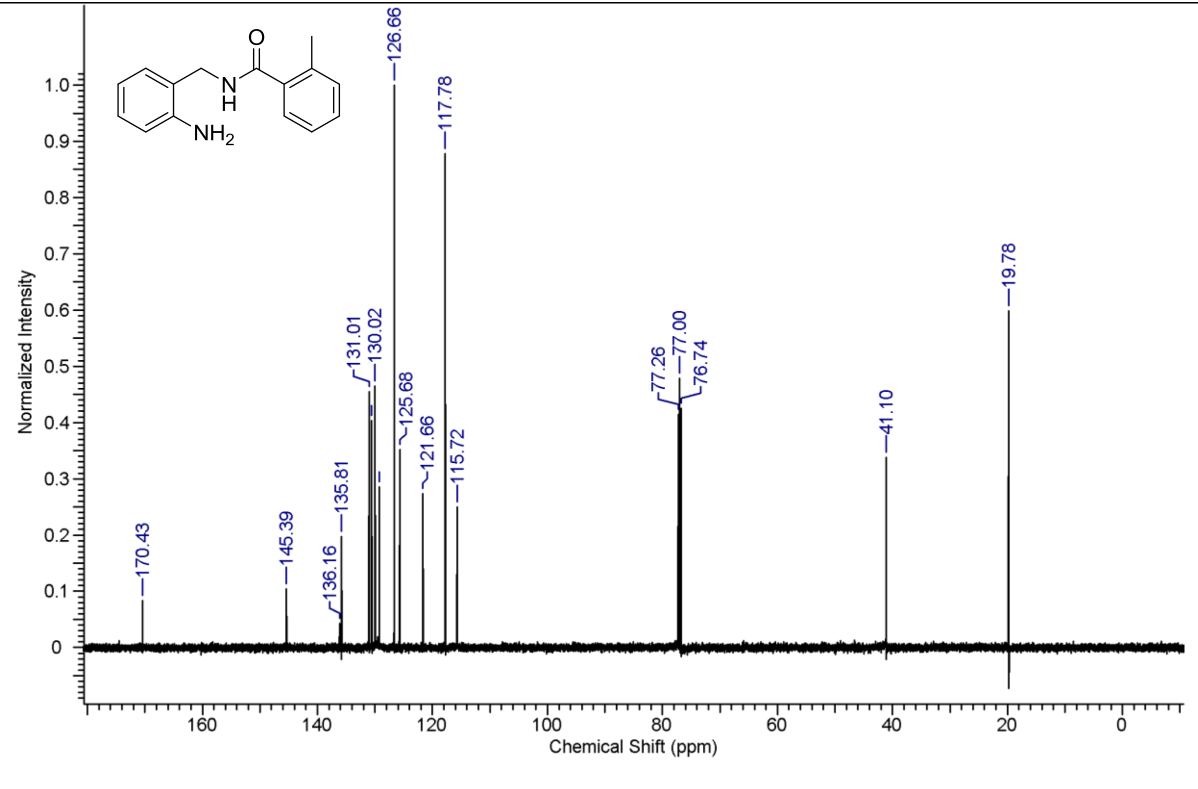

^{1}H NMR (500 MHz, $CDCl_3$) spectrum of compound **4g**

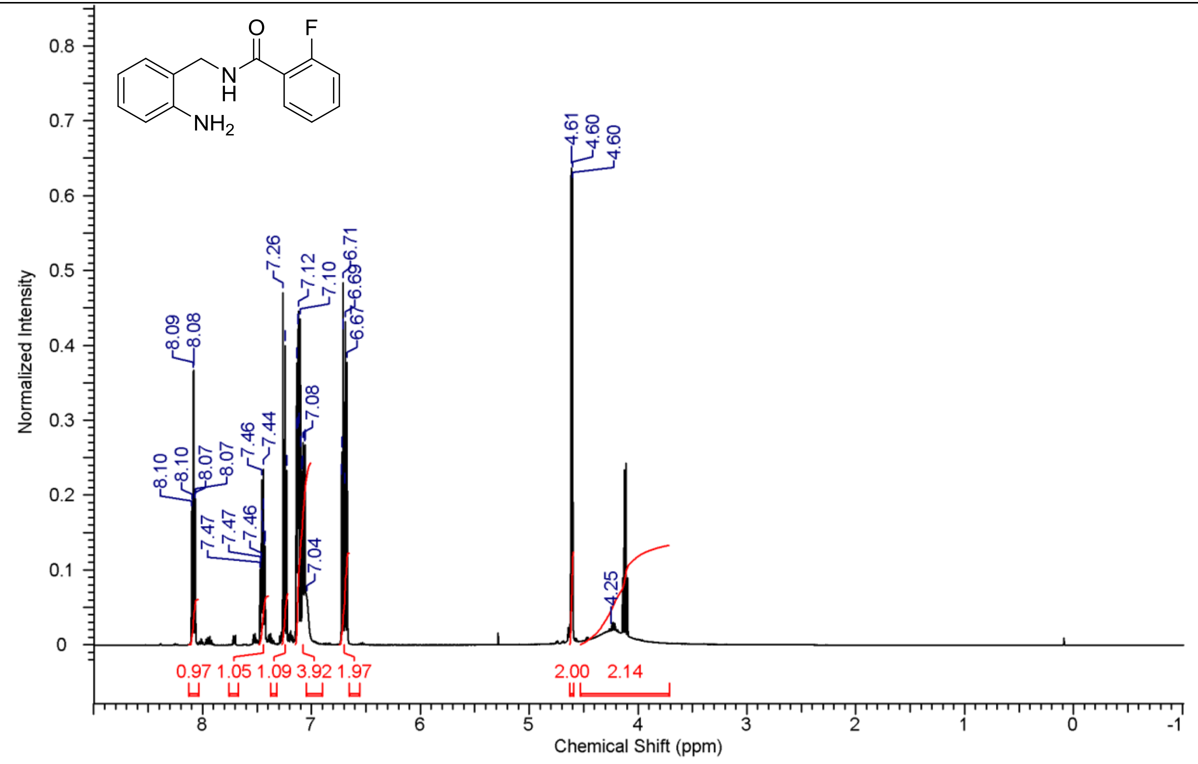

^{13}C NMR (125 MHz, $CDCl_3$) spectrum of compound **4g**

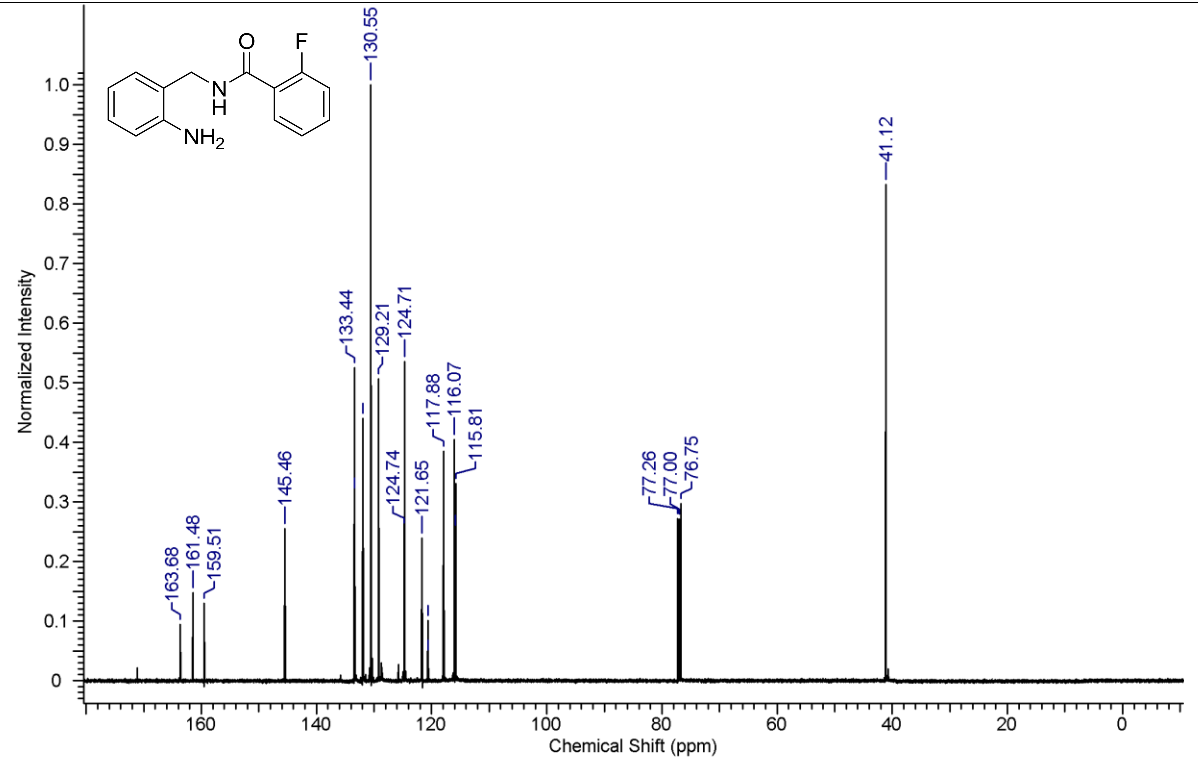

^{1}H NMR (500 MHz, $CDCl_3$) spectrum of compound **4h**

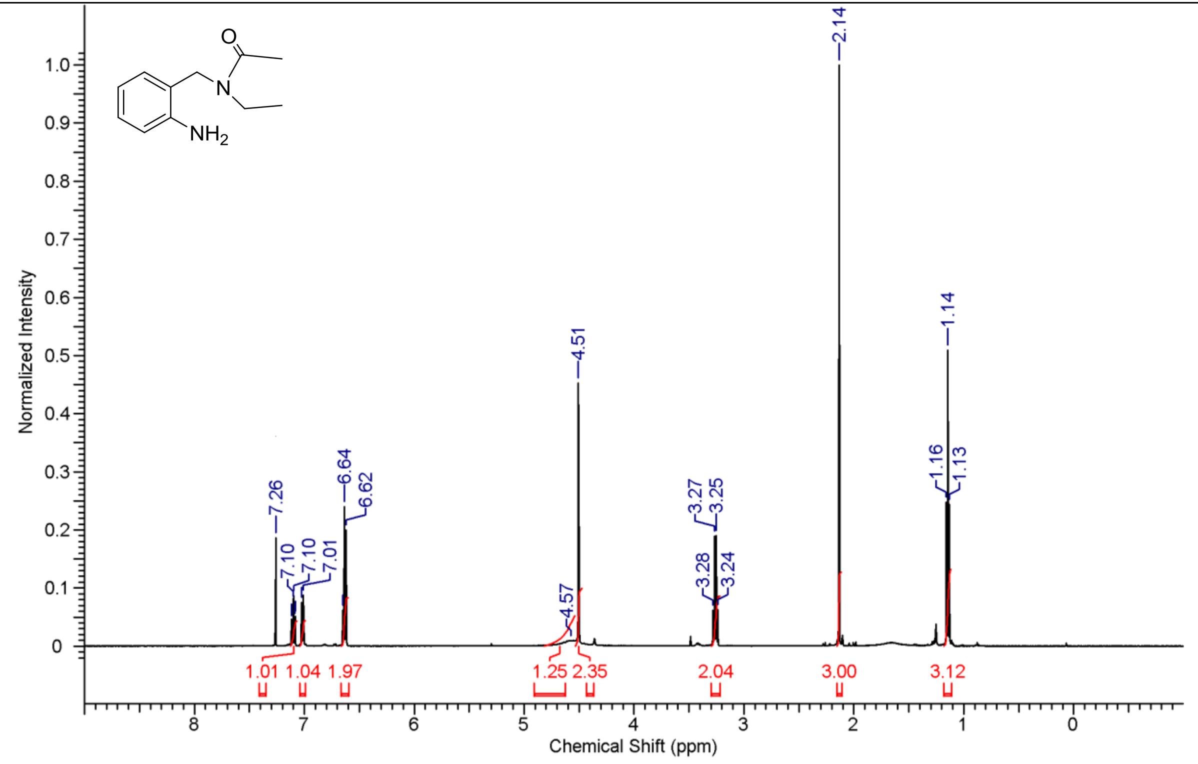

^{13}C NMR (125 MHz, $CDCl_3$) spectrum of compound **4h**

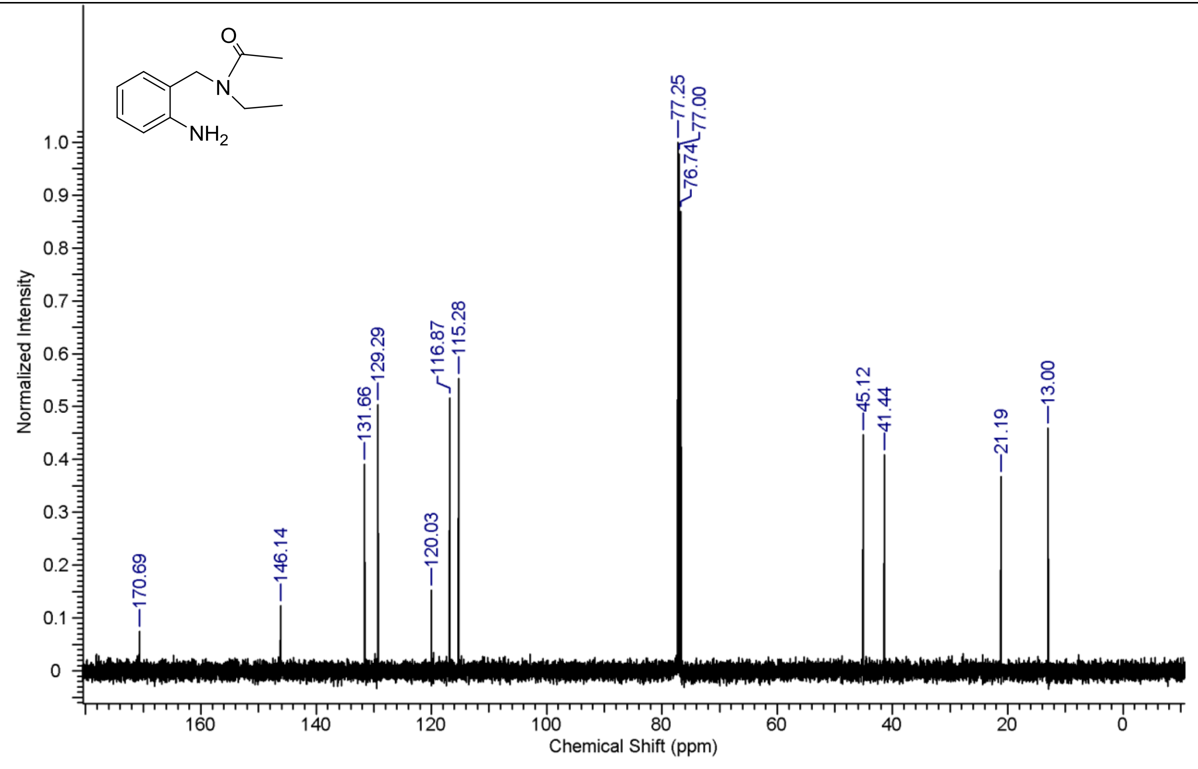

^{1}H NMR (500 MHz, $CDCl_3$) spectrum of compound **4i**

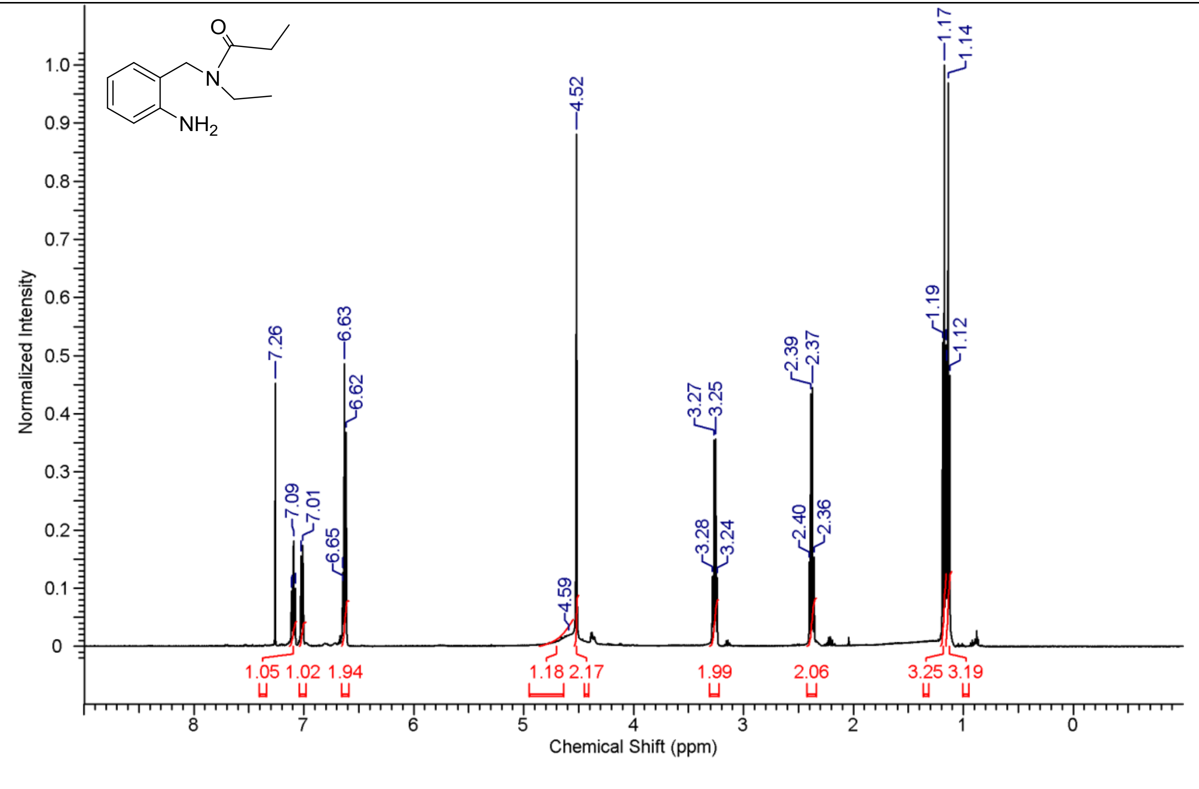

^{13}C NMR (125 MHz, $CDCl_3$) spectrum of compound **4i**

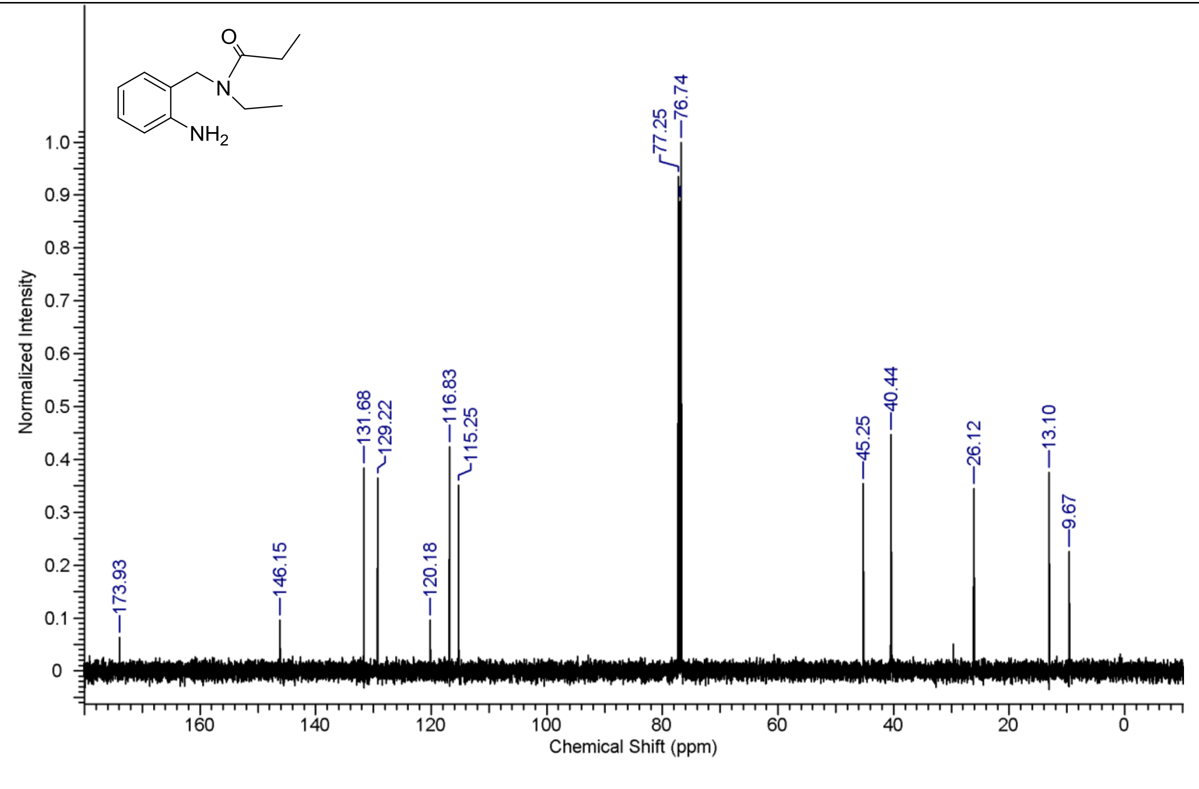

^{1}H NMR (500 MHz, $CDCl_3$) spectrum of compound **4j**

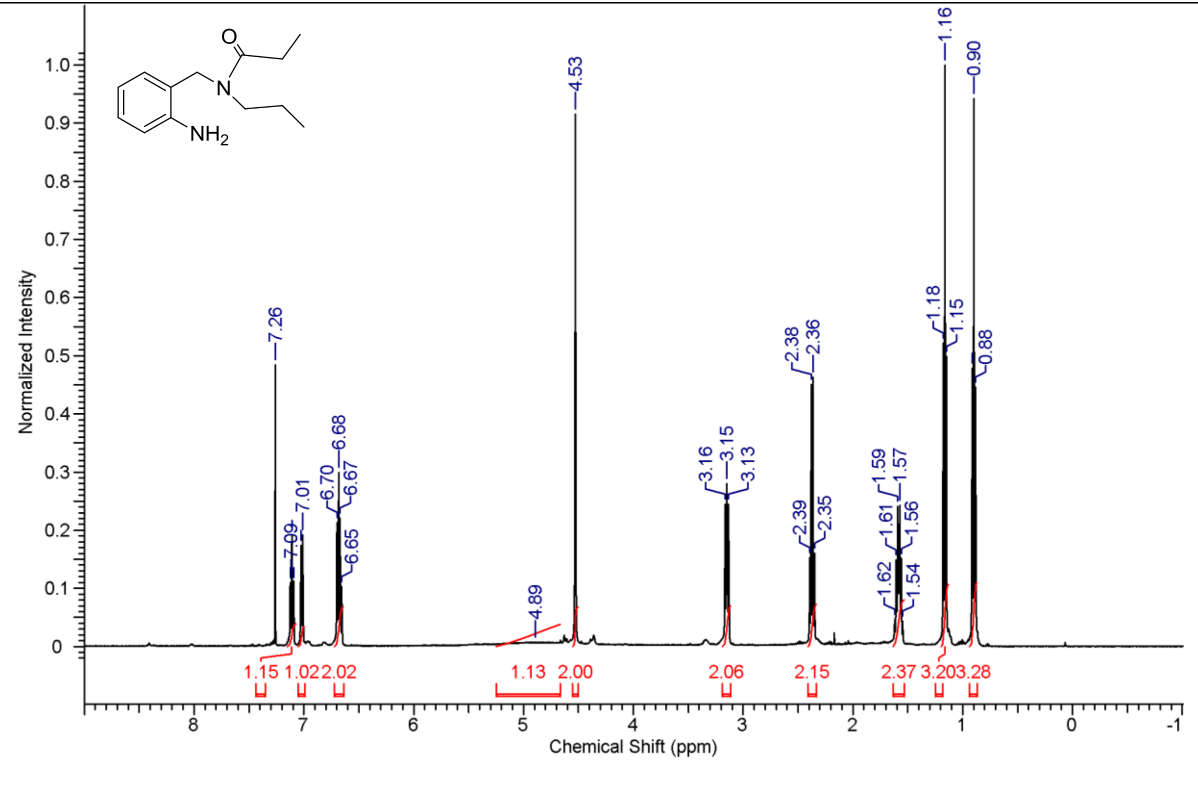

^{13}C NMR (125 MHz, $CDCl_3$) spectrum of compound **4j**

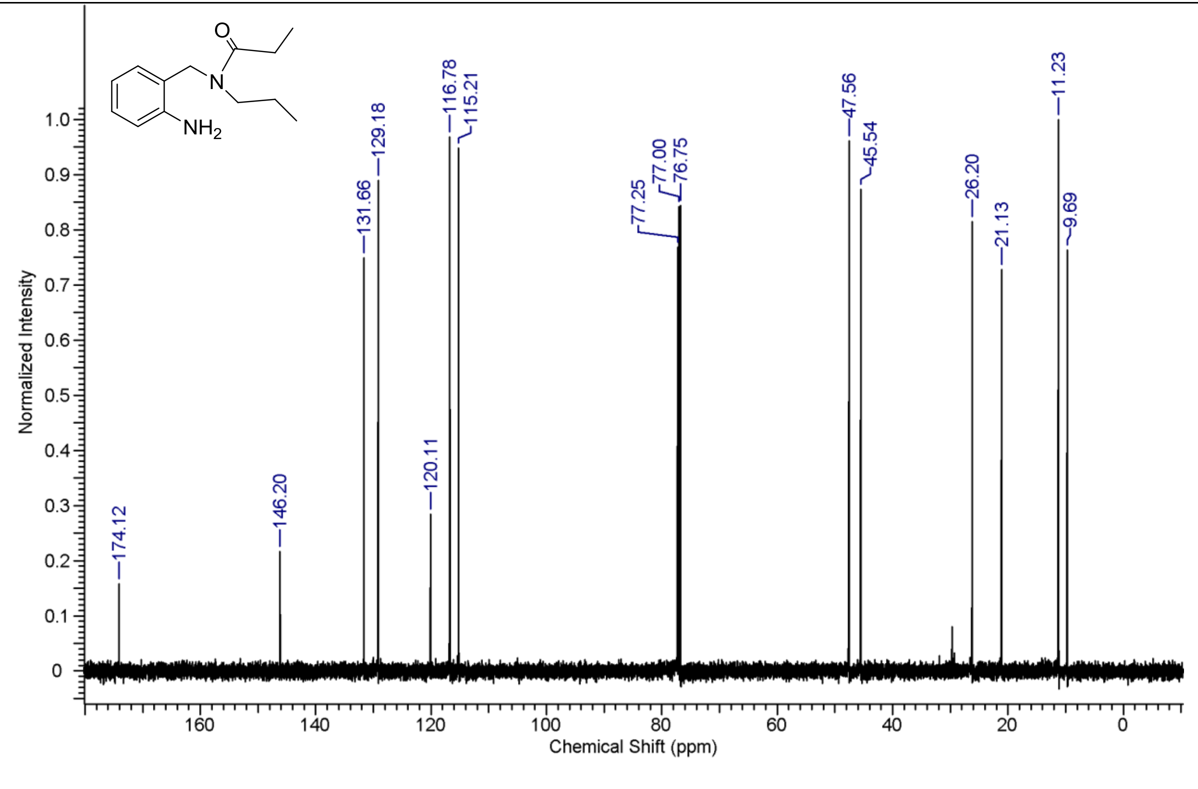

^{1}H NMR (500 MHz, $CDCl_3$) spectrum of compound **4k**

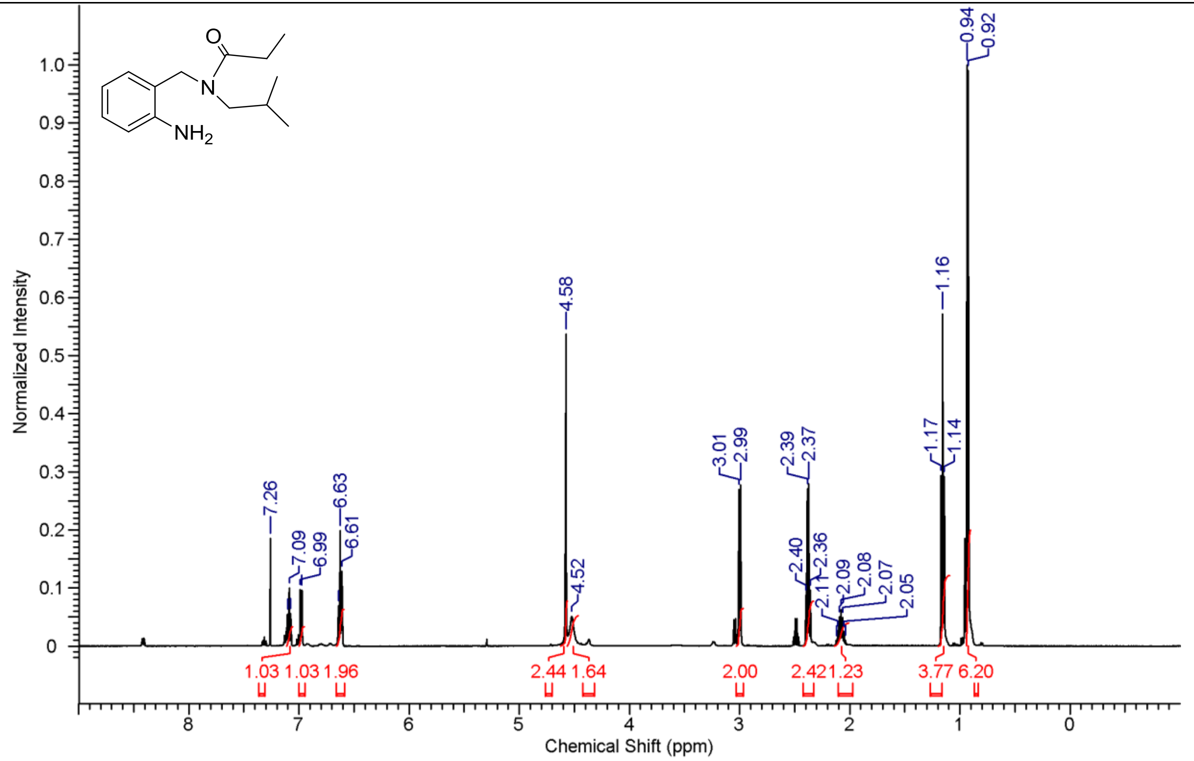

^{13}C NMR (125 MHz, $CDCl_3$) spectrum of compound **4k**

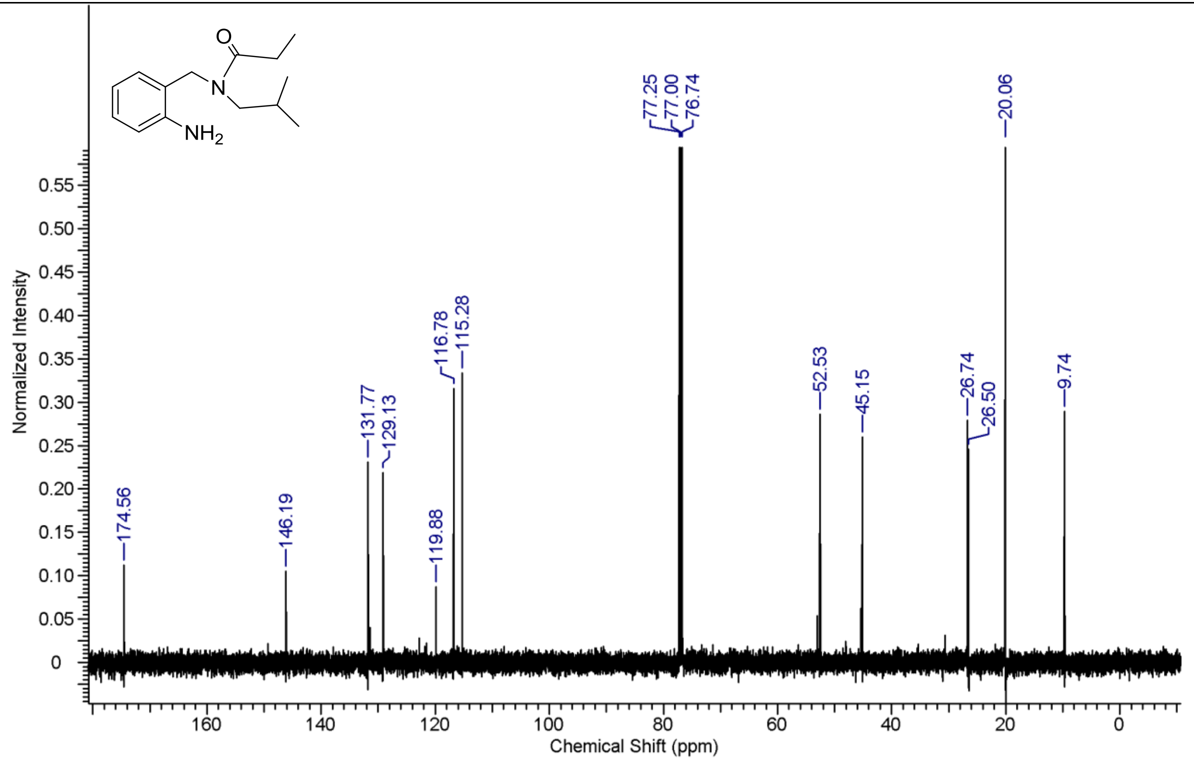

^{1}H NMR (500 MHz, $CDCl_3$) spectrum of compound **5a**

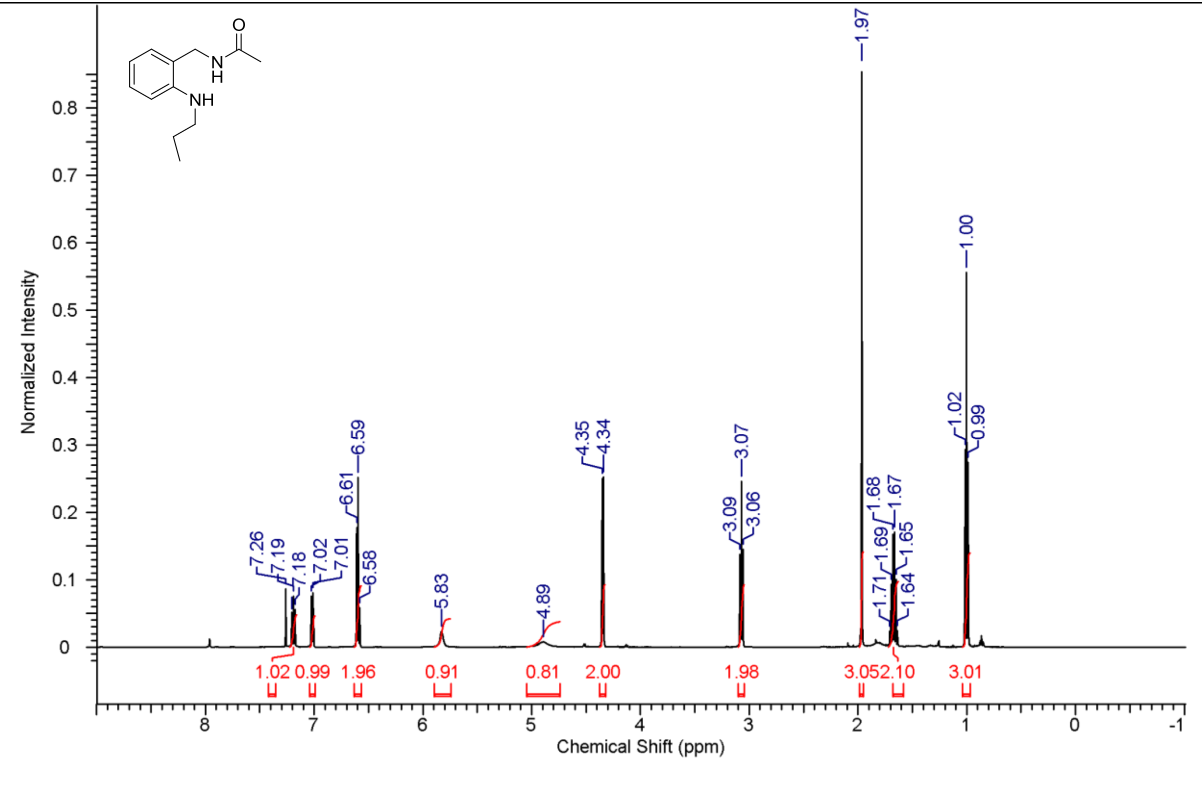

^{13}C NMR (125 MHz, $CDCl_3$) spectrum of compound **5a**

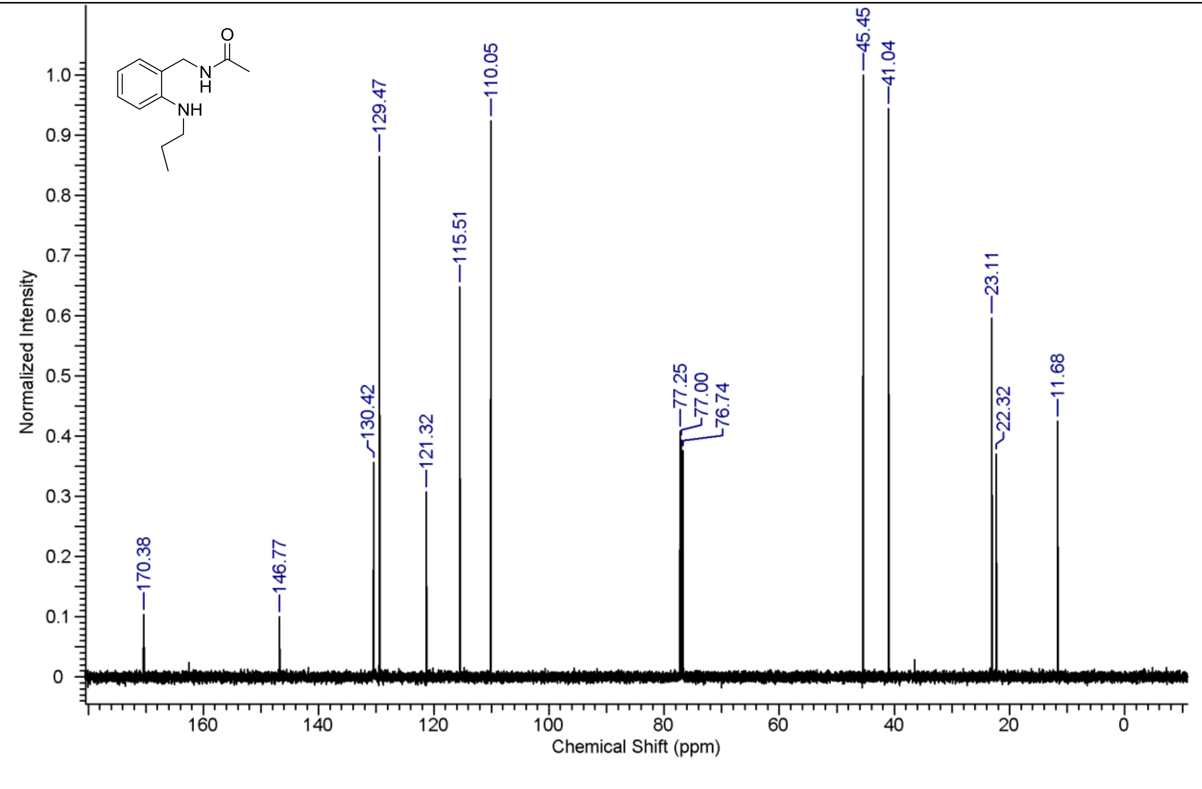

^{1}H NMR (500 MHz, $CDCl_3$) spectrum of compound **5b**

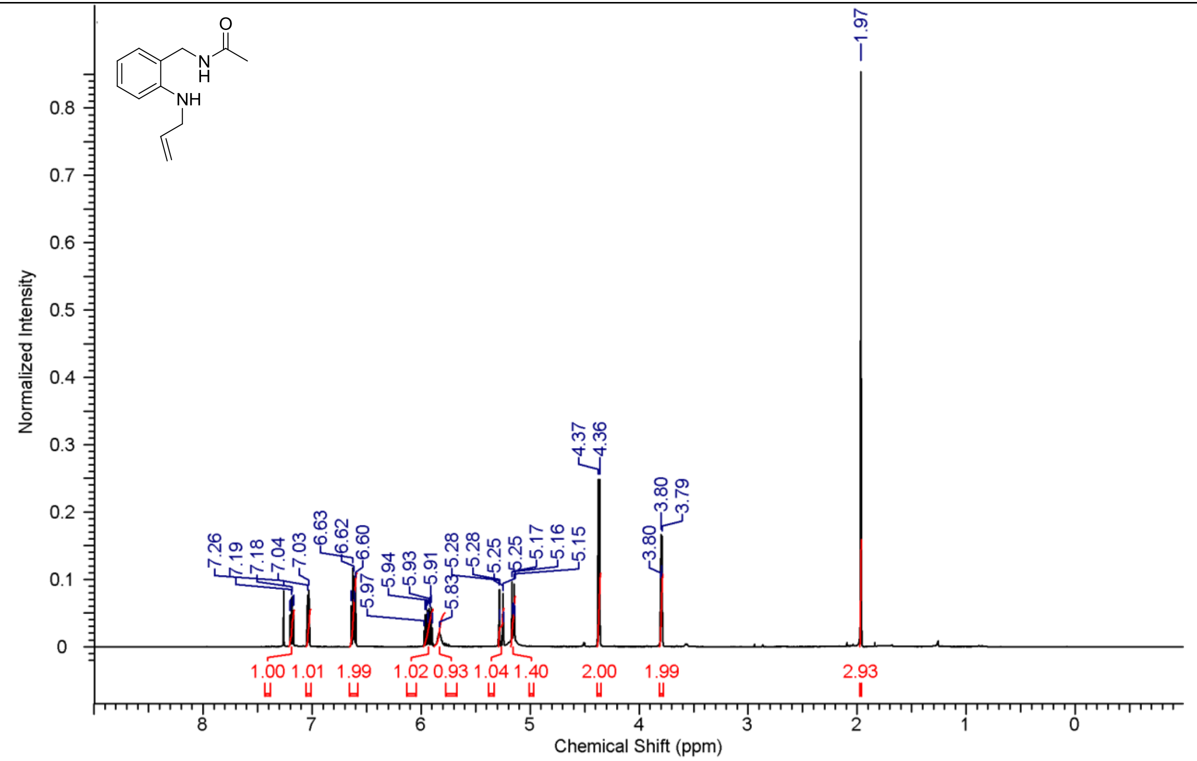

^{13}C NMR (125 MHz, $CDCl_3$) spectrum of compound **5b**

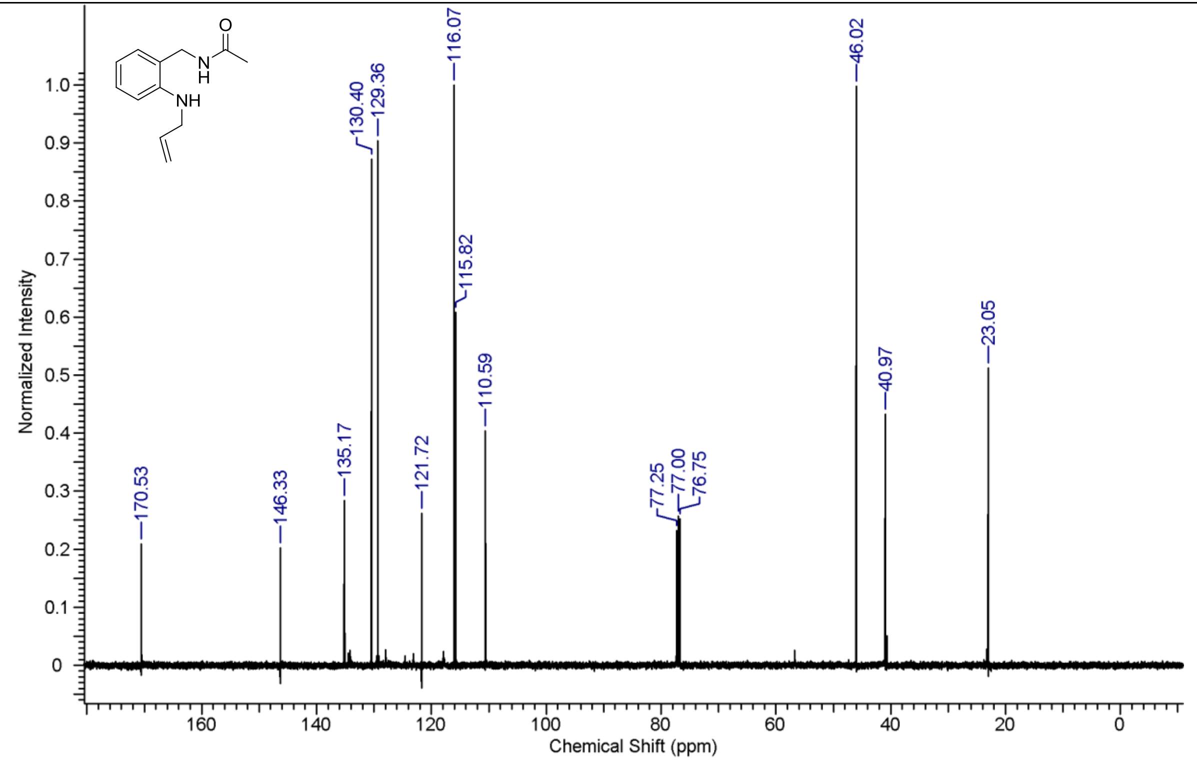

^{1}H NMR (500 MHz, $CDCl_3$) spectrum of compound **5c**

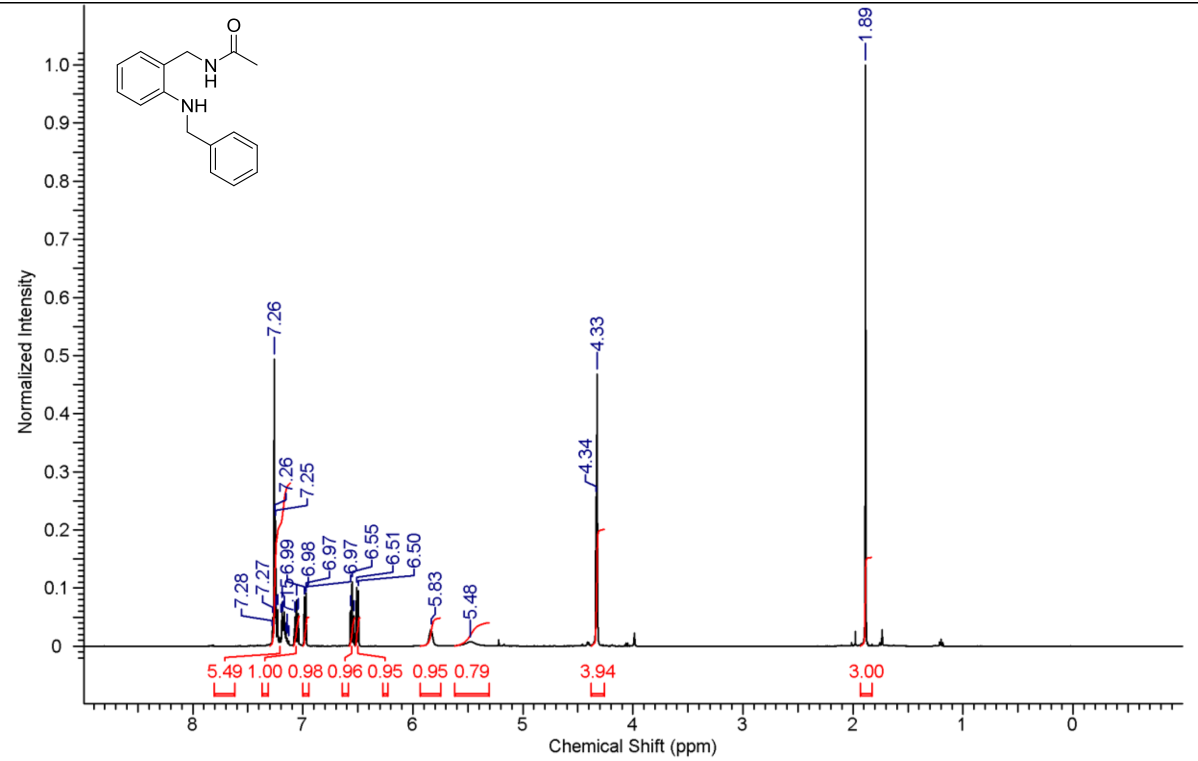

^{13}C NMR (125 MHz, $CDCl_3$) spectrum of compound **5c**

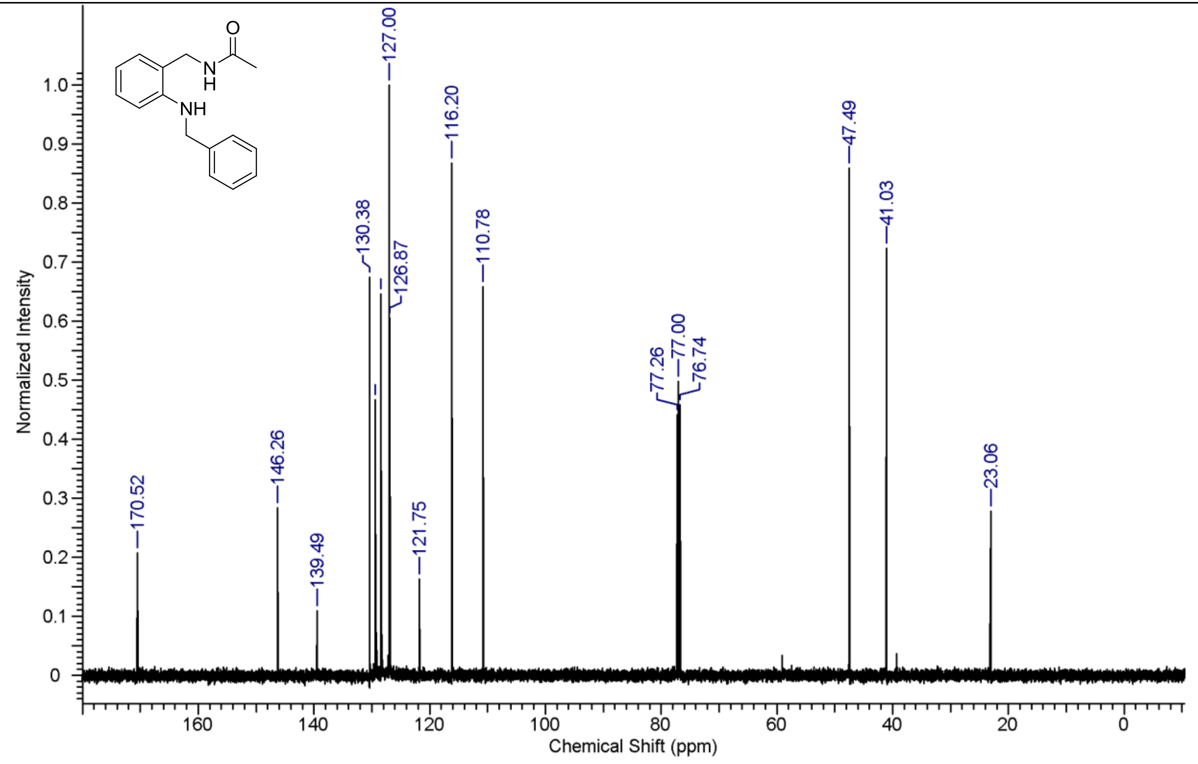

^{1}H NMR (500 MHz, $CDCl_3$) spectrum of compound **5d**

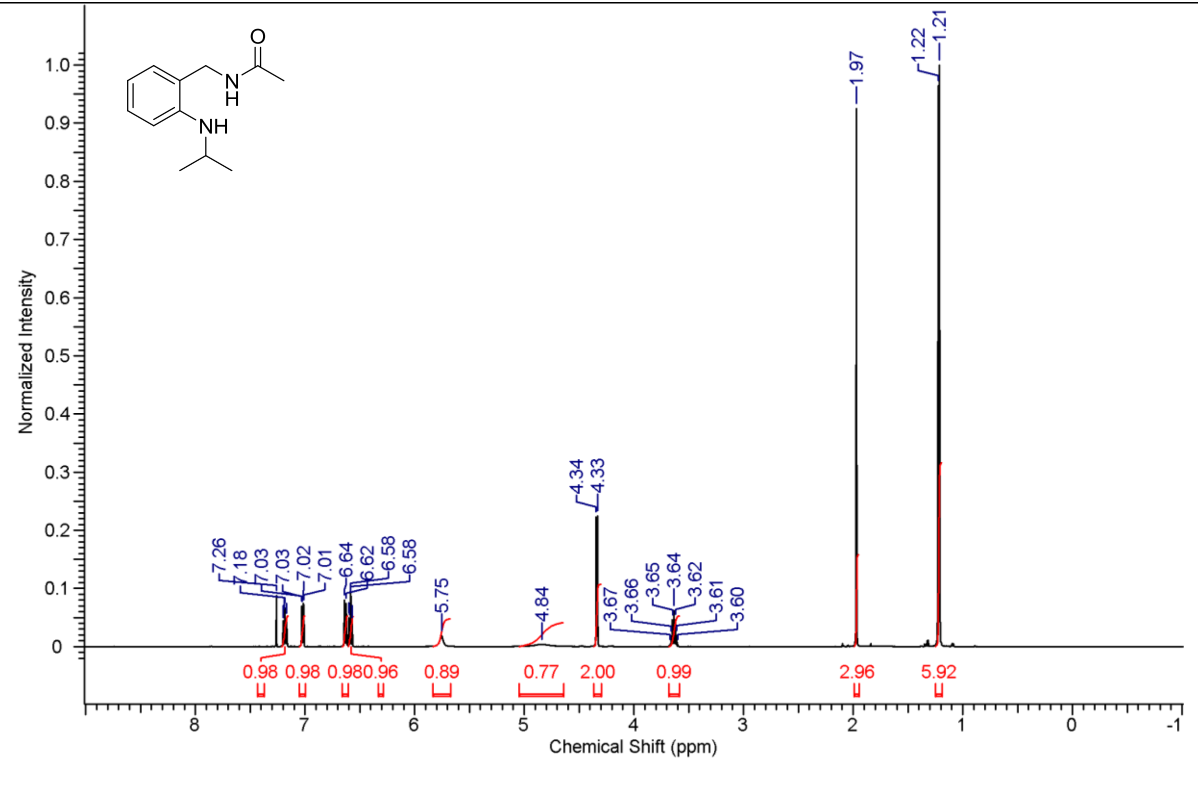

^{13}C NMR (125 MHz, $CDCl_3$) spectrum of compound **5d**

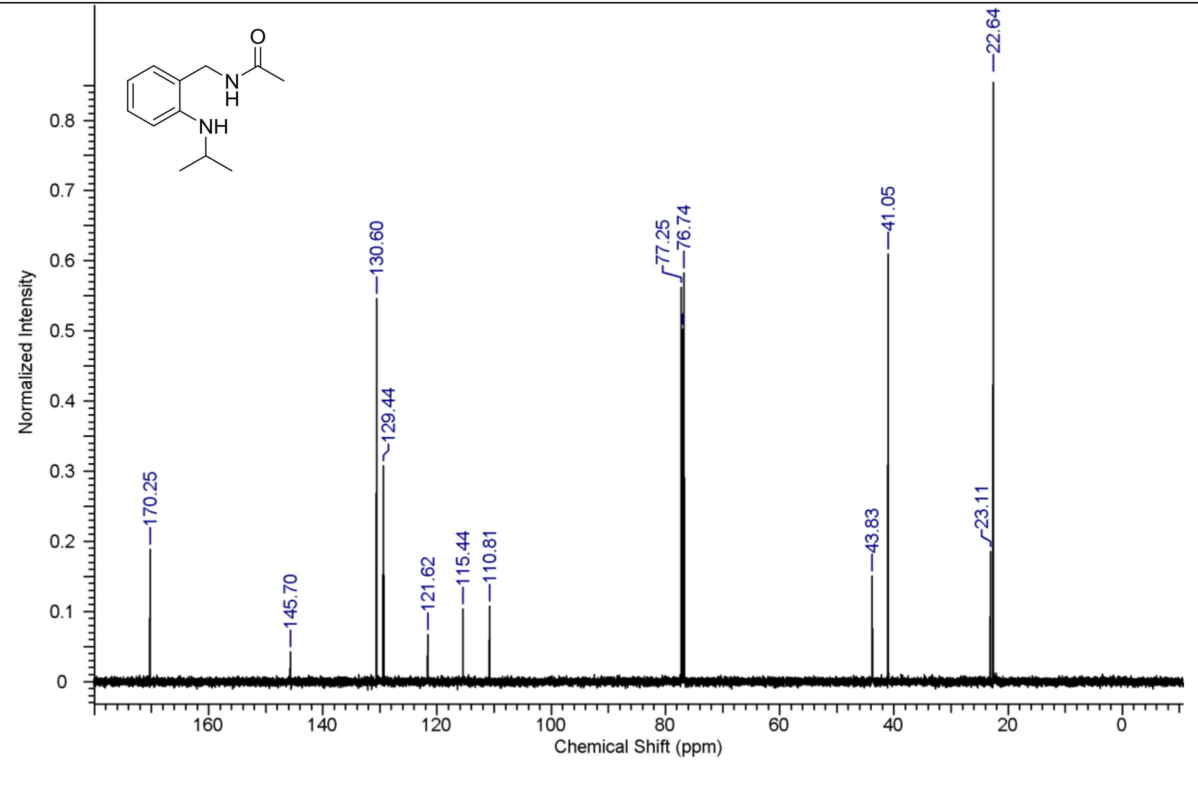

^{1}H NMR (500 MHz, $CDCl_3$) spectrum of compound **5e**

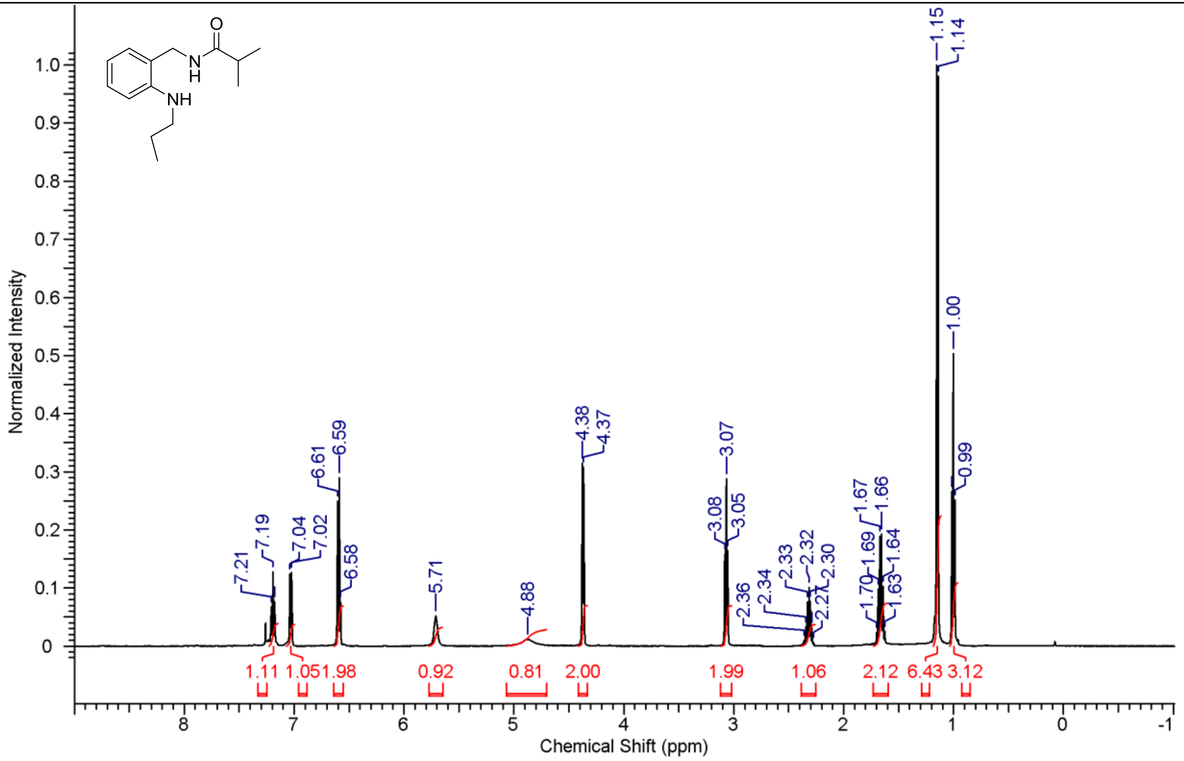

^{13}C NMR (125 MHz, $CDCl_3$) spectrum of compound **5e**

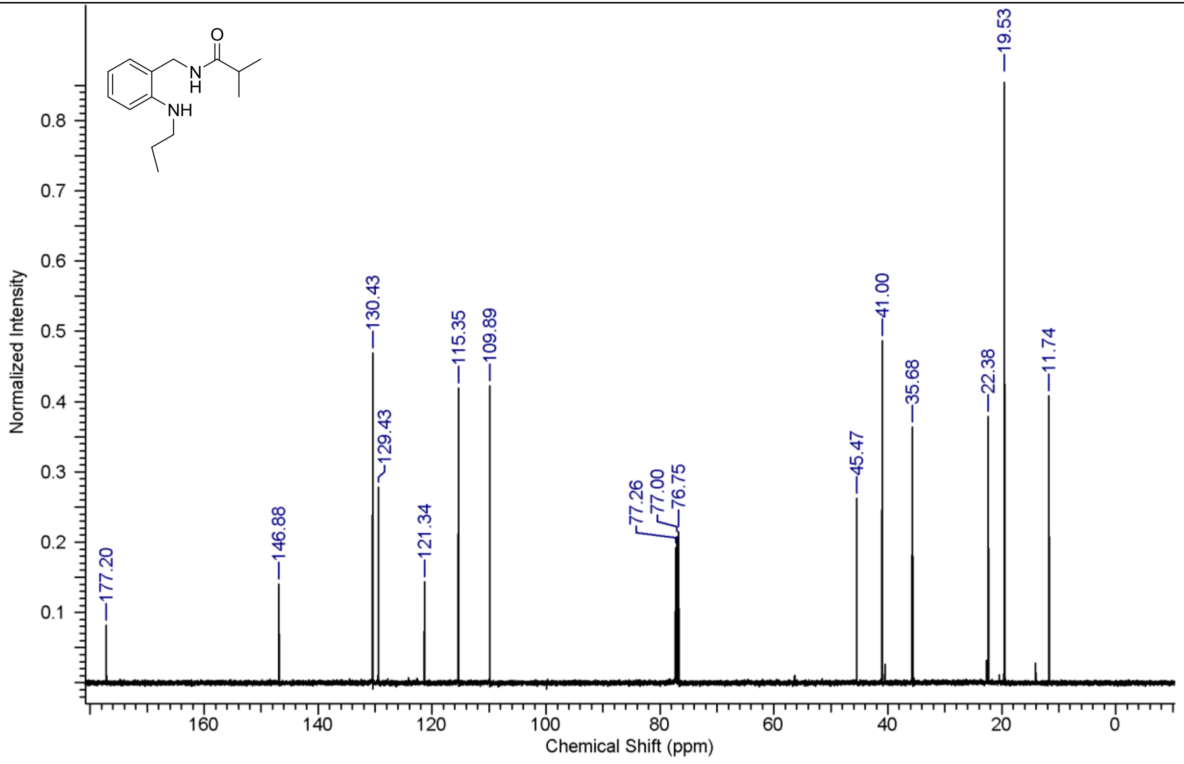

^{1}H NMR (500 MHz, $CDCl_3$) spectrum of compound **5f**

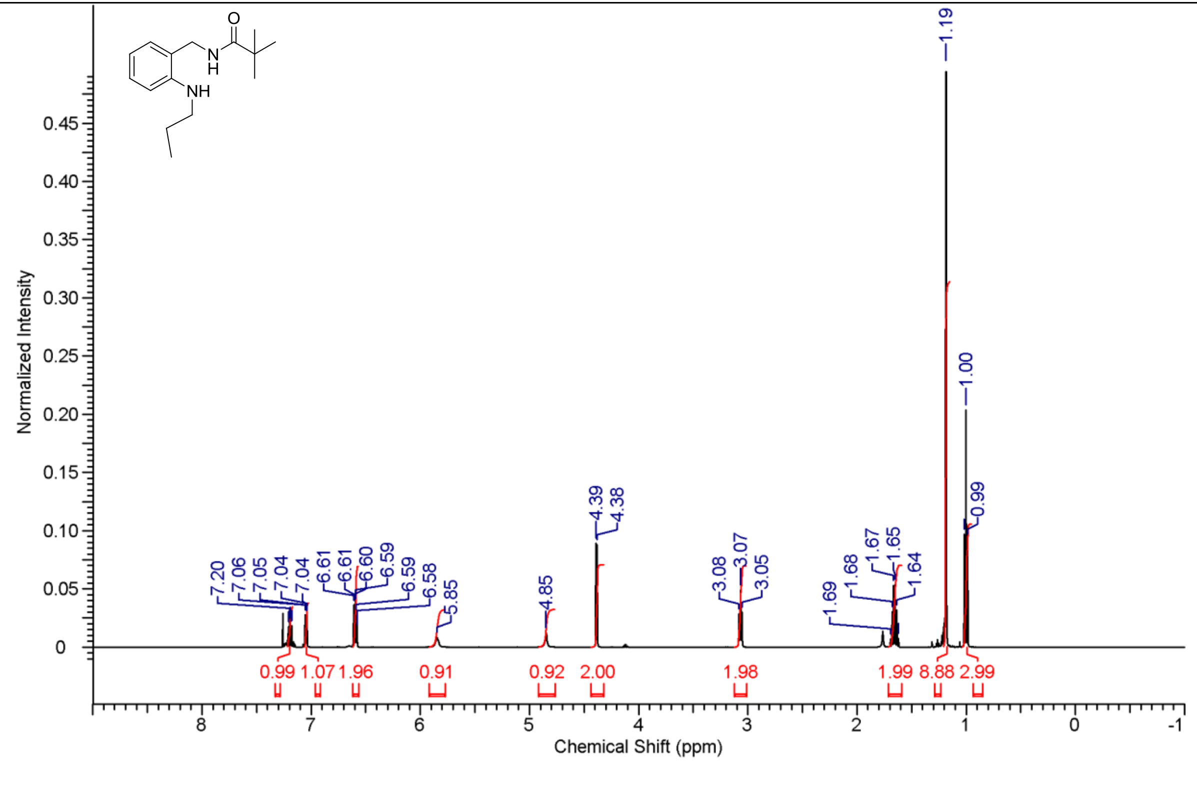

^{13}C NMR (125 MHz, $CDCl_3$) spectrum of compound **5f**

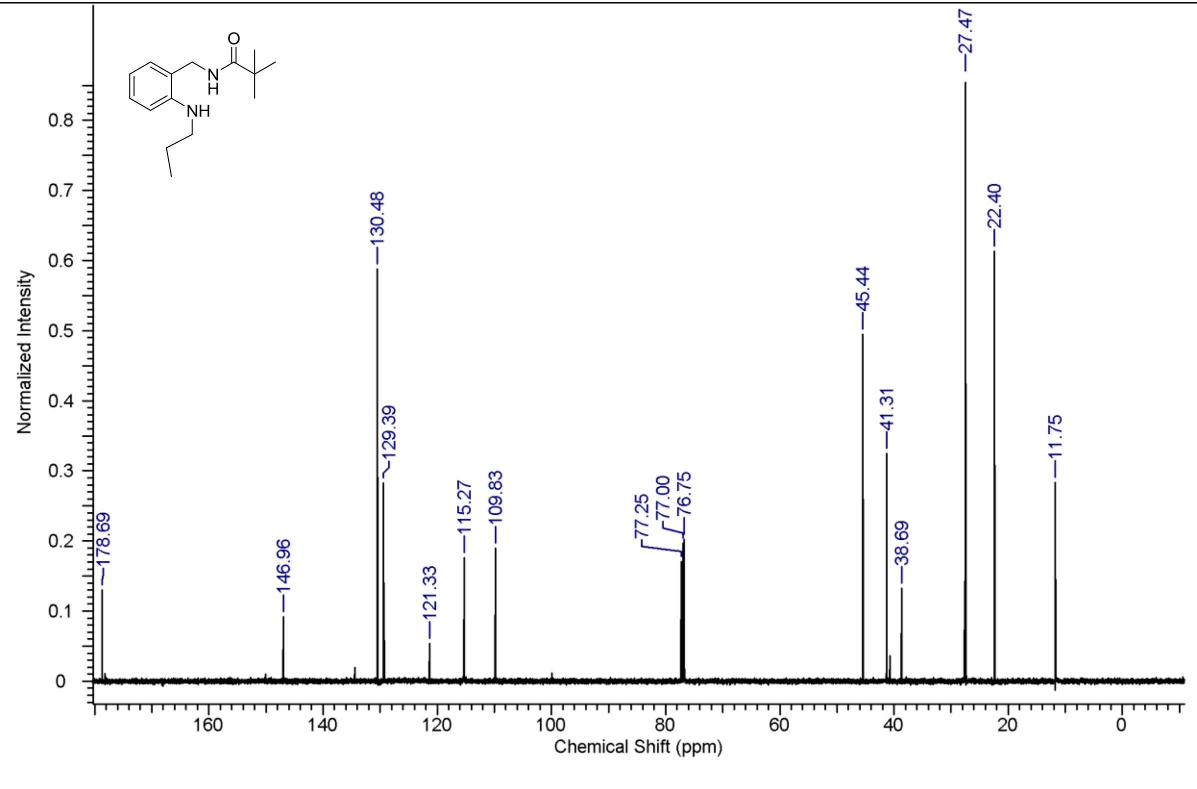

^{1}H NMR (300 MHz, $CDCl_3$) spectrum of compound **5g**

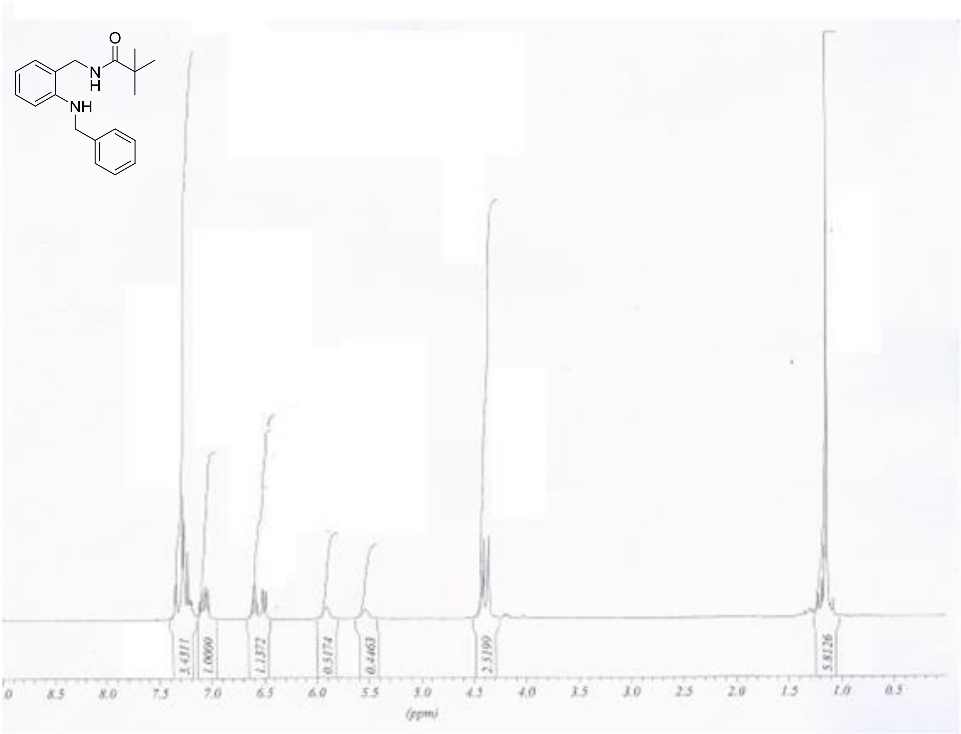

^{13}C NMR (75 MHz, $CDCl_3$) spectrum of compound **5g**

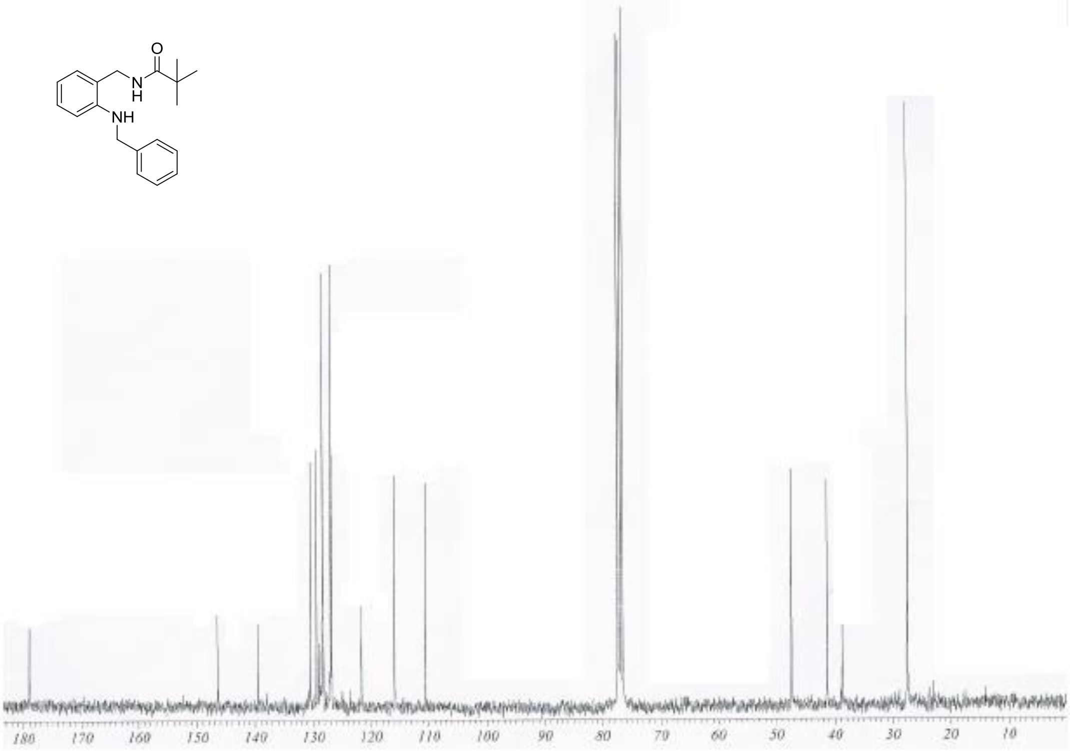

^{1}H NMR (600 MHz, $CDCl_3$) spectrum of compound **5h**

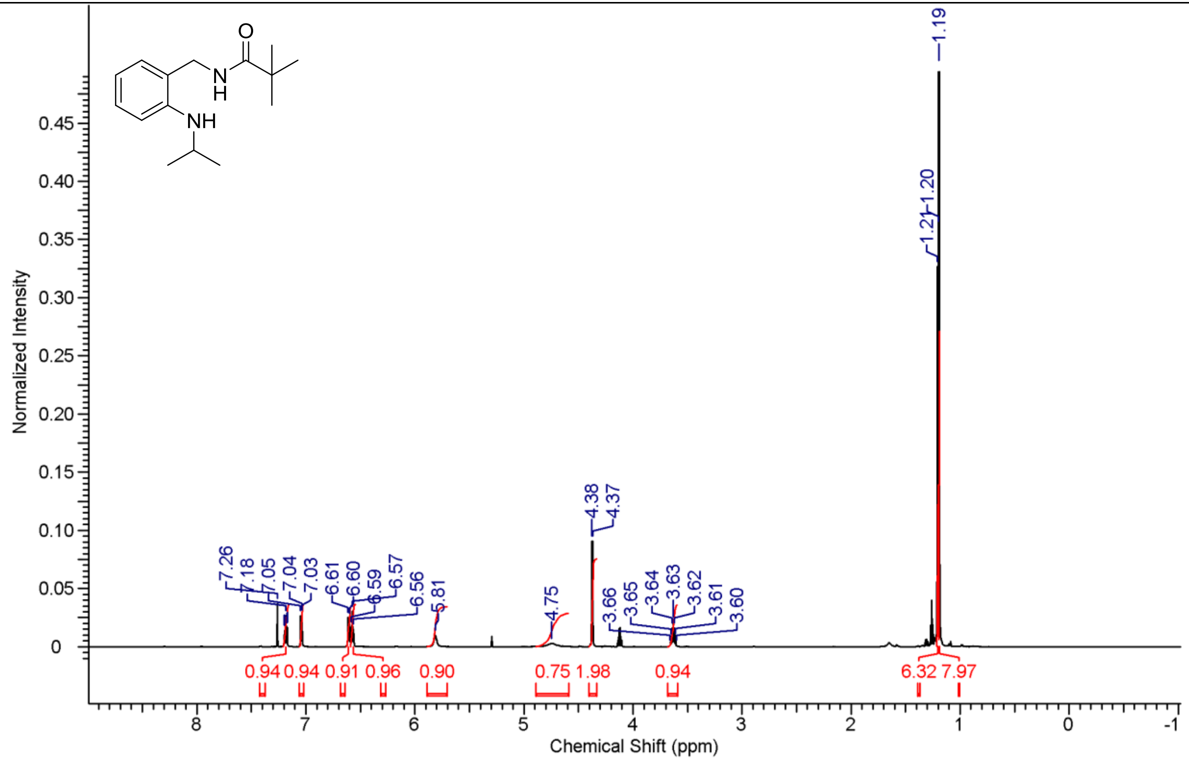

^{13}C NMR (150 MHz, $CDCl_3$) spectrum of compound **5h**

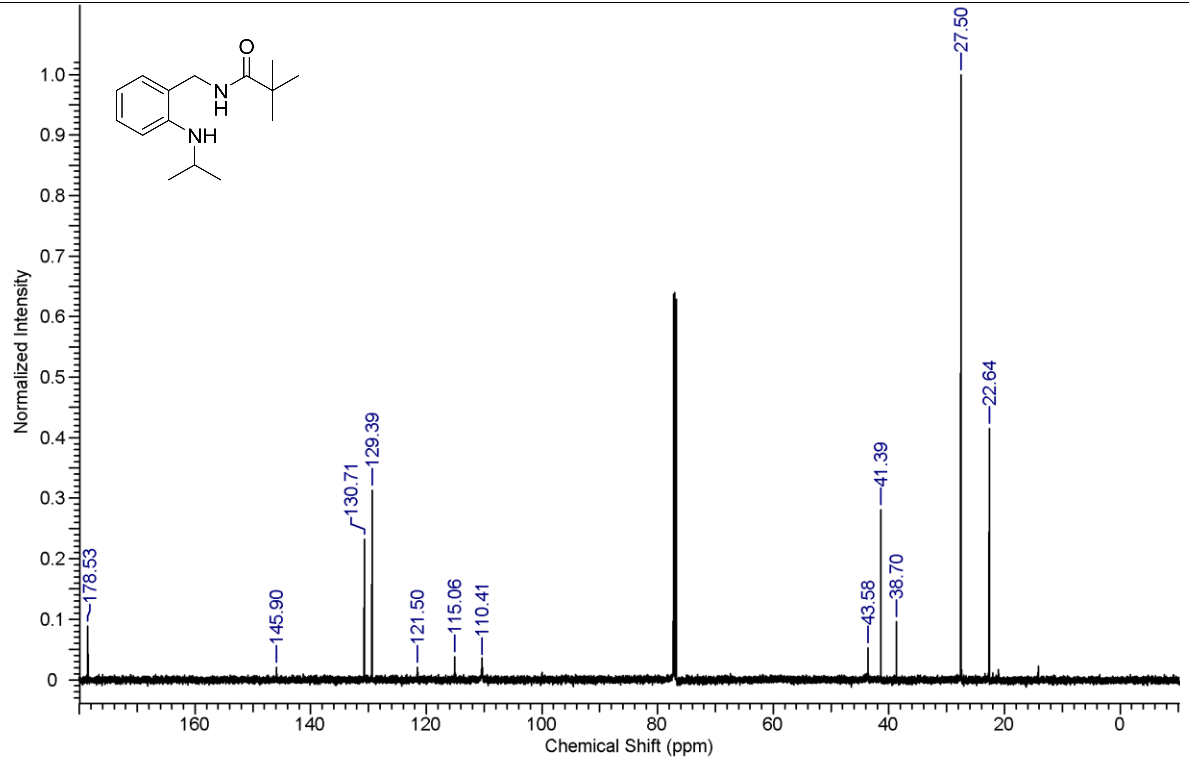

^{1}H NMR (500 MHz, $CDCl_3$) spectrum of compound **5i**

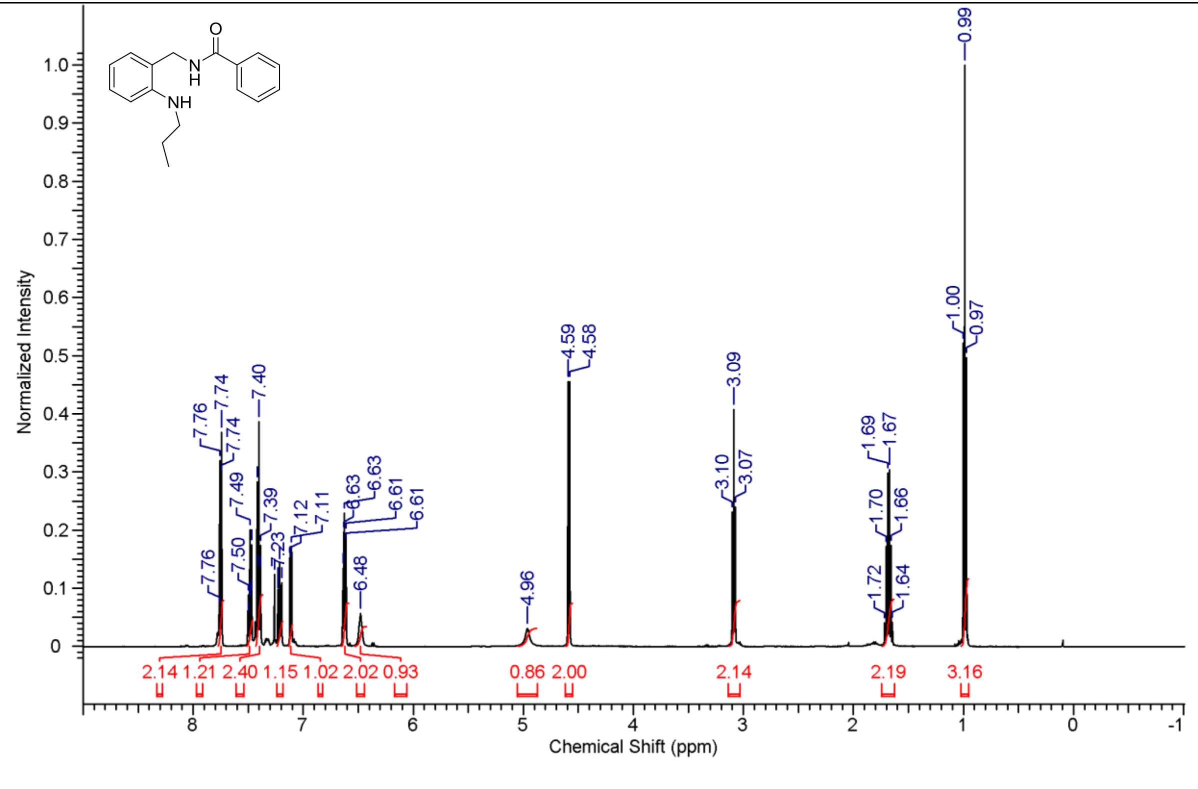

^{13}C NMR (125 MHz, $CDCl_3$) spectrum of compound **5i**

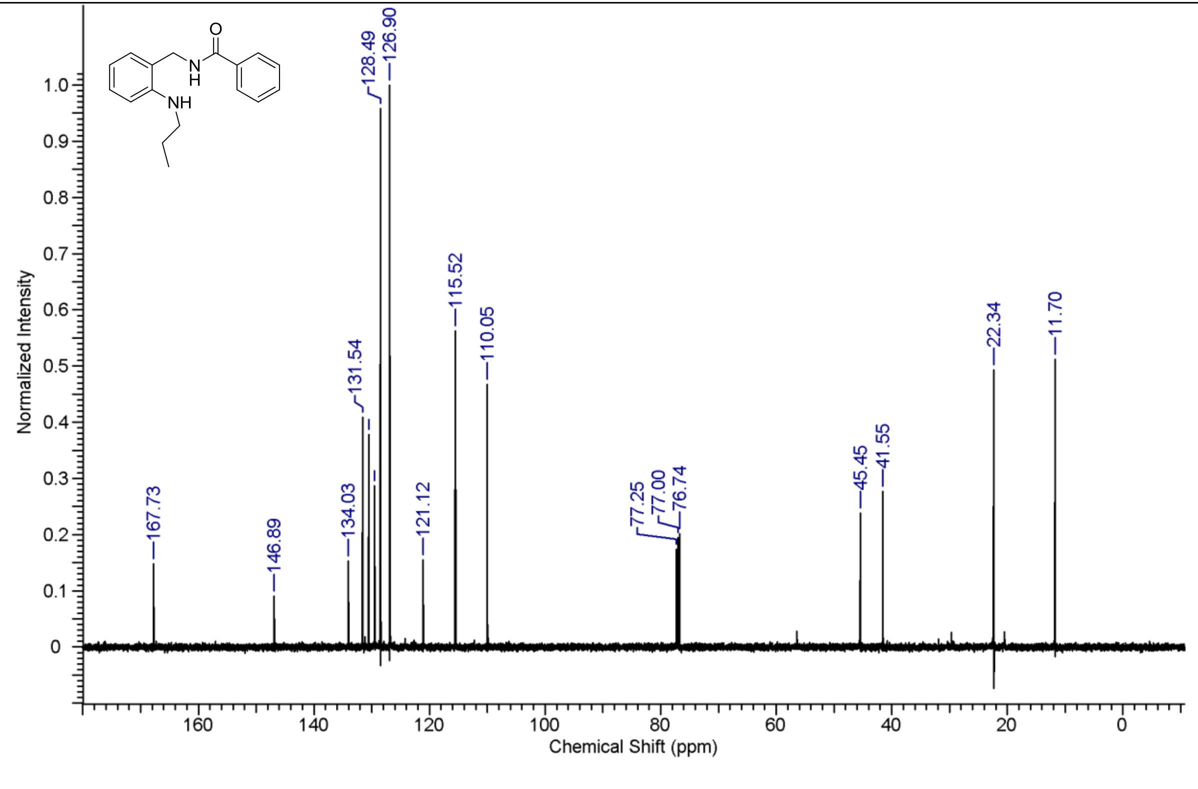

^{1}H NMR (500 MHz, $CDCl_3$) spectrum of compound **5j**

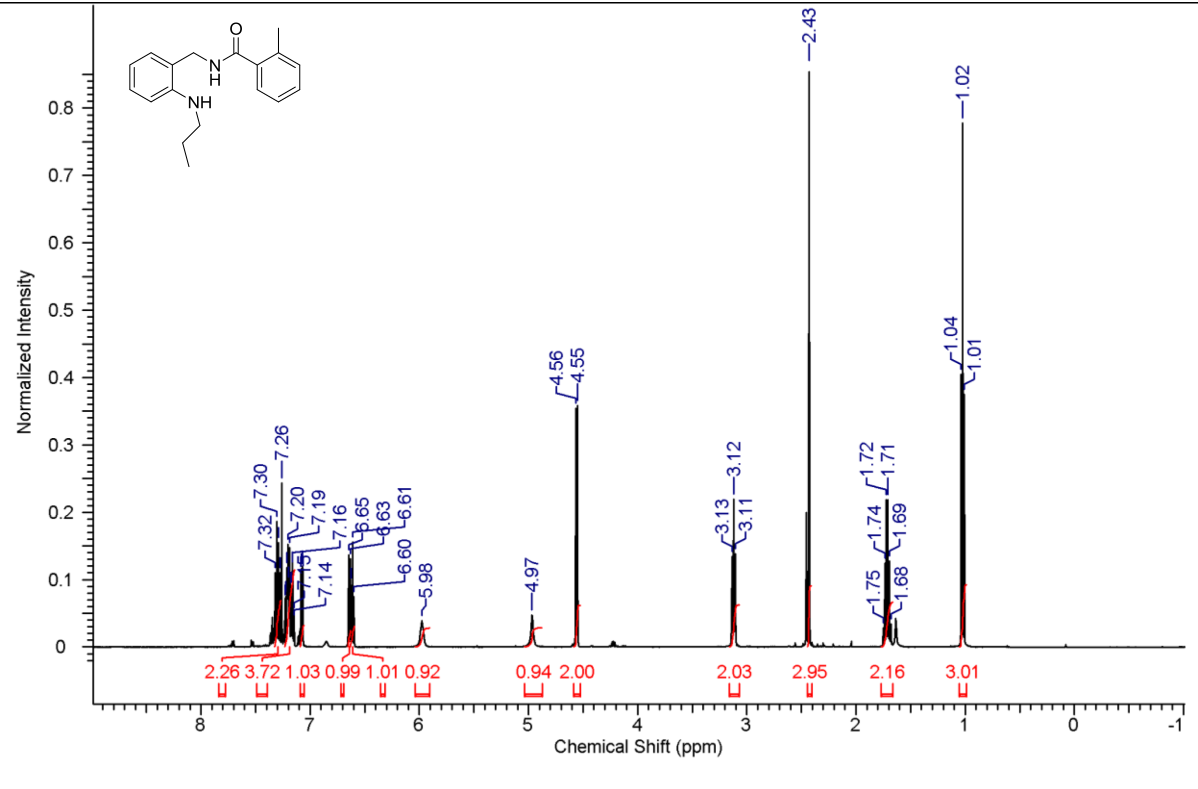

^{13}C NMR (125 MHz, $CDCl_3$) spectrum of compound **5j**

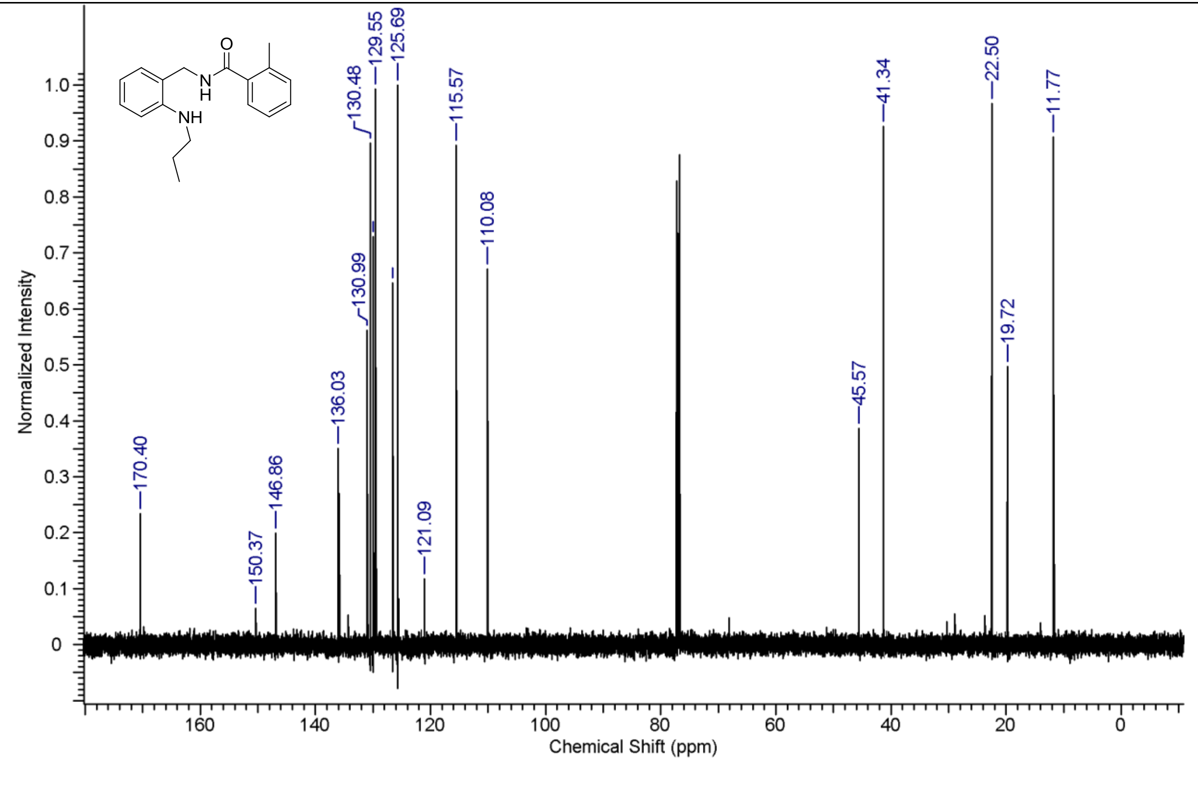

5. References

[1] Cava, M. P.; Lakshmikantham, M. V.; Mitchell, M. J., *J. Org. Chem.* **1969**, 34, 2665-2667;

[2] Vogel, A. I., Furniss, B. S.; Hannaford, A. J.; Smith, P. W. G.; Tatchell, A. R. *Vogel´s Textbook of Practical Organic Chemistry*, 5th ed.; Longman Scientific & Technical: London, 1989; p. 418.

[3] Smith, J. G; Sheepy, J. M. *J. Heterocycl. Chem.* **1975**, 12, 231-234.

[4] Sato, O.; Seshimo, M.; Tsunetsugu, J. *J. Chem. Res. S*, **1998**, 568-569.

[5] Camacho, M. E.; Chayah, M.; García, M. E.; Fernández-Sáez, N.; Arias, F. *Arch. Pharm. Chem. Life Sci.* **2016**, 349, 1–13.

[6] Pelagalli, R.; Chiarotto, I.; Feroci, M.; Vecchio, S. *Green Chem.* **2012**, 14, 2251-2255.

[7] Chakrabarty, M.; Chakravarty, A. K.; Pakrashi, S. C. *Heterocycles* **1983**, 20, 445-450.

[8] Nguyen, H. T. H.; Hoang, L. T. M.; Ngo, L. H.; Nguyen, H. L.; Nguyen, C. K.; Nguyen, B. T.; Ton, Q. T.; Nguyen, H. K. D.; Cordova, K. E.; Truong, T. *Chem. Commun.* **2015**, 51, 17132-17135.

[9] Yadav, D. K. T.; Bhanage, B. M. *Synlett* **2015**, 26, 1862-1866.

[10] Armarego, W. L. F. *J. Chem. Soc.* **1961**, 2697-2701.
